# Structural, linear, and nonlinear optical properties of *ortho*-carboranyl luminophores: insights from DFT and TD-DFT studies

**DOI:** 10.1039/d6ra00681g

**Published:** 2026-04-22

**Authors:** Djamila Samsar, Douniazed Hannachi, Meriem Zaidi, Olivier Aroule, Guillaume Hoffmann, Henry Chermette

**Affiliations:** a Institut D'Hygiène et Sécurité Industrielle, Département de Socle Commun Hygiène et Sécurité Industrielle, Université de Batna-2 Algerie; b Laboratory of Materials Chemistry and the Living: Activity & Reactivity (LCMVAR), Department of Chemistry, Faculty of Matter Sciences, University of Batna 1 Algeria; c Laboratory of Electrochemistry, Molecular Engineering and Redox Catalysis, Faculty of Technology, Setif 1 University-Ferhat Abbas Setif 19137 Algeria; d Department of Chemistry, Faculty of Sciences, Setif 1 University-Ferhat Abbas Setif 19137 Algeria; e Laboratoire de Chimie, Ingénierie Moléculaire et Nanostructures (LCIMN), Université Ferhat Abbas Sétif 1 Sétif 19000 Algerie; f Université de Lyon, Université Claude Bernard Lyon 1, Institut des Sciences Analytiques, UMR CNRS 5280 69622 Villeurbanne Cedex France

## Abstract

Three non-centrosymmetric molecular series, namely, *i*M, *i*H, and *i*C, are systematically investigated, each incorporating a donor fragment substituted with various functional groups (R = –CF_3_, –F, –H, –CH_3_, –*t*Bu, –OMe, –OH, –NH_2_, and –NMe_2_) and differing in the nature of the electron-accepting core, with a trimethylsilyl-acetylene unit in *i*M, an *o*-carborane cage in *i*H, and a trimethylsilyl-functionalized *o*-carborane cage in *i*C. The geometries of the ground and first excited states, the absorption and emission electronic transitions, and intrafragment charge transfer are fully characterized using DFT and TD-DFT methods. Furthermore, first- and second-order NLO responses are examined under both static and dynamic regimes. The results show that the *i*C derivatives exhibit slightly higher variations in dipole moment (Δ*µ*), oscillator strength (*f*), Coulomb attractive energy (*E*_CA_), net electron transfer between the substituent (*R*) and the *o*-carborane cage, Stokes shift, and NLO responses compared with the corresponding *i*H derivatives. In contrast, the *i*M molecules display consistently lower values for these parameters. For all series, the magnitude of these properties increases with the electron-donor strength of the R group, with the *i*C series showing an ∼854% rise in *β*_0_ from 1C to 9C. A strong correlation is observed between the first hyperpolarizability and both the net electron transfer between fragments (1 → 3) (*R*^2^ > 0.97) and the Coulomb attractive energy of *i*H and *i*C (*R*^2^ > 0.95). For the *i*C compounds (*i* = 2–6), an excellent linear relationship is also found between the photoluminescence quantum yield (*Φ*_em_) and the static first hyperpolarizability (*R*^2^ = 0.92). Notably, the *o*-carborane derivatives bearing an –NMe_2_ substituent demonstrate the potential to serve as highly efficient second-order NLO materials.

## Abstract 2

Relationship between the fluorescence quantum yield and first hyperpolarizability in non-centrosymmetric compounds.

## Graphical abstract

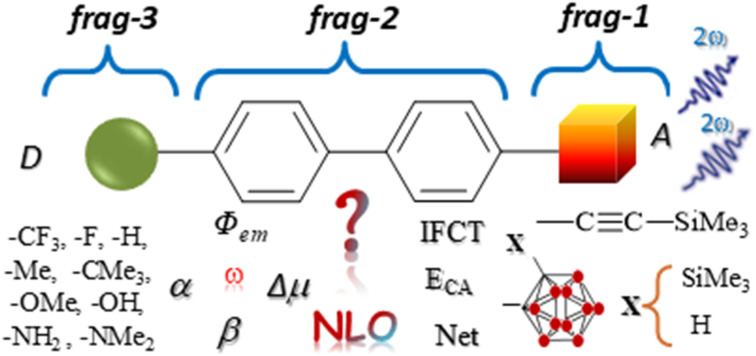

## Introduction

Organic and inorganic π-conjugated materials with tailored donor–acceptor (D–A) architectures have attracted significant research attention in recent years owing to their potential in organic photovoltaic (OPV),^1^ optoelectronic^2^ and photonic applications, including photodetectors,^3^ organic light-emitting diodes (OLEDs), dyes,^4–6^ fluorescence sensors^7^ and nonlinear optical (NLO) devices.^8–10^ Furthermore, D–A systems are known to exhibit a strong intramolecular charge transfer (ICT) process from the donor to the acceptor, which makes them promising candidates for near-infrared (NIR) NLO applications.^1,11–13^

Three-dimensional electron acceptors constitute an important class of molecular architectures for nonlinear optical (NLO) applications, among which fullerenes have been extensively studied owing to their exceptional electron affinity, highly delocalized π-conjugated spherical surface, and remarkable capability to stabilize long-lived charge-separated states.^14,15^ Since the pioneering characterization of C_60_, numerous fullerene–chromophore dyads have been developed by covalently integrating C_60_ with a wide range of electron-donating units,^16^ including porphyrins,^17,18^ tetrathiafulvalenes (TTF),^19^ ferrocenes^20^ and carbazoles.^21^ These donor–acceptor conjugates have demonstrated substantial NLO activity, highlighting their efficiency in promoting intramolecular charge-transfer (ICT) processes.^22,23^

Muhammad *et al.*^24^ systematically investigated a series of C_60_ fulleropyrrolidine and fulleropyrrolidine–tetrathiafulvalene derivatives and reported strong second-order NLO responses, with their *β* values ranging from 1.73 × 10^−30^ to 15.69 × 10^−30^ esu. The highest value was obtained for compound {2c}, which is attributed to its enhanced intramolecular charge transfer and extended π-conjugation. In parallel, Fouejio *et al.*^25^ demonstrated that the functionalization of C_60_ with dihydroartemisinin dramatically boosts its optical response, increasing its first hyperpolarizability from 1.21 × 10^−30^ to 3.47 × 10^−30^ esu. These studies confirm that the chemical modification of fullerenes significantly enhances their CT character and NLO activity, positioning them as benchmark 3D acceptors in computational NLO design.

Despite these achievements, fullerenes still face intrinsic limitations that restrict their broader technological impact. These include their high production cost, combined with the centrosymmetric nature of pristine C_60_ (which necessitates asymmetric functionalization to induce a non-zero first hyperpolarizability)^26^ and processability challenges in devices.^27^ Consequently, they have motivated the development of alternative 3D acceptors possessing improved physicochemical and electronic characteristics.

In this context, the *ortho-closo*-carborane cluster (C_2_B_10_H_10_) has emerged as one of the most promising substitutes for fullerene-based electronic acceptors. This cluster, defined by its icosahedral σ-aromatic boron cage, is frequently described as a three-dimensional analogue of benzene, but it exhibits a distinct and highly polarized electronic structure.^28–30^ Unlike fullerene derivatives, carboranes combine a substantial permanent dipole moment (4–5 Debye for *ortho*-carborane),^31^ exceptional thermal stability (>400 °C) and superior synthetic accessibility,^32,33^ thereby positioning them as attractive scaffolds for advanced photonic materials.^34^ The high polarizability of the boron cage induces a pronounced electron-withdrawing effect on carbon-bound substituents, enabling efficient charge separation within donor–acceptor frameworks.^35–39^ Recent investigations have further highlighted the versatility of carborane–chromophore conjugates, demonstrating their promising performance across a broad spectrum of functional materials, including NLO systems,^40–42^ electro-optic materials,^40–42^ light-emitting diodes^43,44^ and organic solar cells.^45^ Beyond photonic applications, they have found use in medicine,^33,46^ supramolecular and materials chemistry, and coordination and organometallic chemistry.^47,48^

In 2023, You *et al.*^49^ synthesized and characterized a series of six *o*-carboranyl luminophores functionalized with biphenyl moieties bearing systematically varied para-substituents (R = –CF_3_, –F, –H, –CH_3_, –*t*Bu, and –OCH_3_) to elucidate the influence of electronic effects on ICT behavior and radiative decay efficiency. Optical studies revealed progressive redshifts in both absorption and emission maxima with increasing electron-donating ability, accompanied by a systematic increase in photoluminescence quantum yield. The radiative decay rate constants remained essentially invariant, whereas nonradiative decay was markedly suppressed for electron-rich derivatives. Collectively, these findings demonstrate that precise modulation of the electronic environment in *o*-carborane-biphenyl systems enable control over the ICT efficiency and emission output, providing valuable design principles for next-generation *o*-carborane-based emissive materials.

**Fig. 1:**
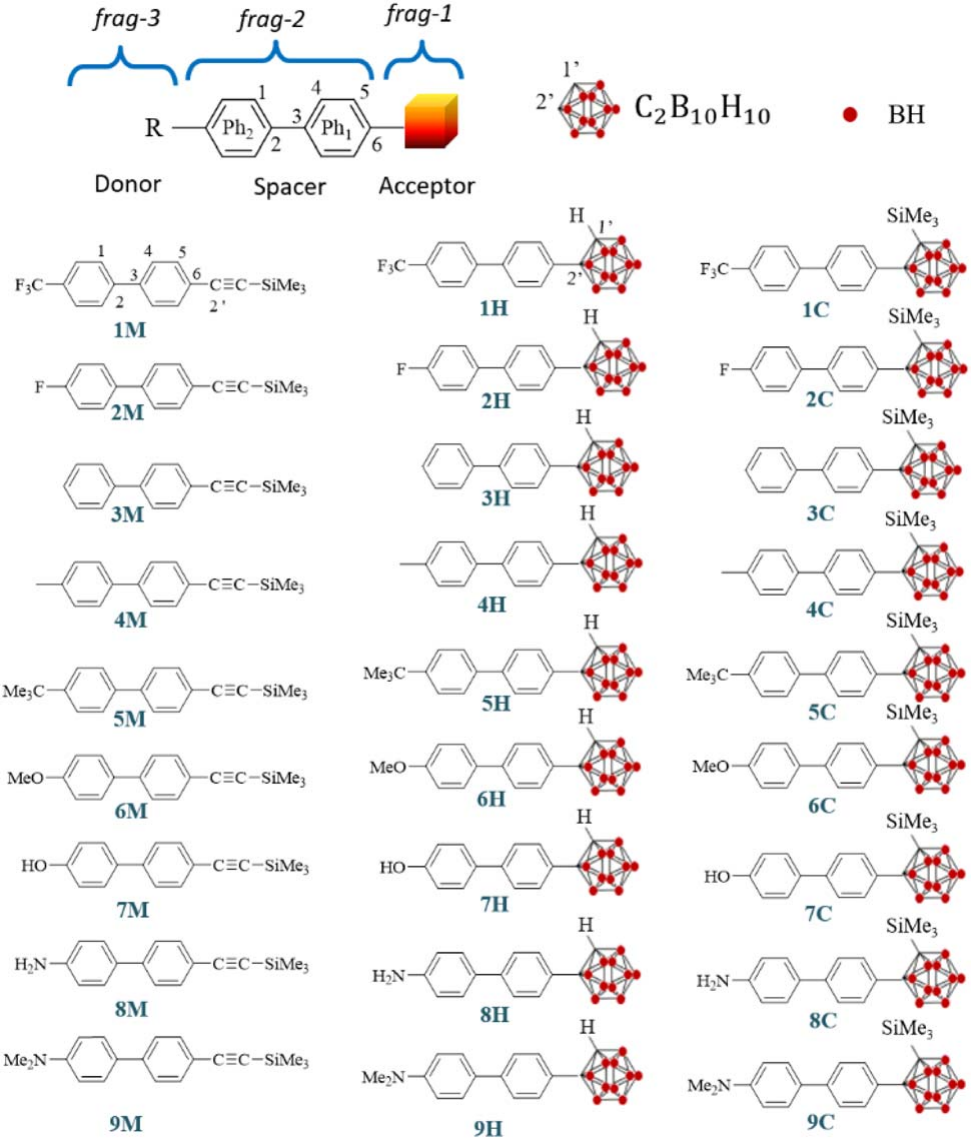
Chemical structures of the title compounds *i*M, *i*H and *i*C (*i* = 1 to 9).

The discovery of new nonlinear optical materials remains a central challenge in advanced materials science for both theoretical and experimental researchers. Quantum chemical calculations have proven to be a powerful approach, providing not only a means to screen and identify promising molecular candidates but also to reveal previously unexplored parameters that can be exploited to predict and control NLO responses with greater accuracy. In this study, DFT and TD-DFT calculations were performed on three non-centrosymmetric molecular series, *i*M, *i*H, and *i*C, of compounds (*i* = 1–9) from six *o*-carboranyl luminophores (1C to 6C) originally synthesized by You *et al.*,^49^ as depicted in Fig. 1. The primary objective of this study, which is carried out on 27 compounds, is to examine the effect of various donor substituents [R = –CF_3_, –F, –H, –Me, –CMe_3_, –OMe, –OH, –NH_2_ and –NMe_2_] on the nonlinear optical properties of the target compounds. In addition, this work aims to evaluate how substituting the *o*-carborane acceptor with a –C≡C–SiMe_3_ fragment influences the NLO response, and to determine the impact of incorporating a bulky trimethylsilyl (TMS) group into the *o*-carborane cage on both the linear and nonlinear optical properties of the *i*H and *i*C series. The principal goal of this study is to establish new parameters that can serve as reliable predictors of NLO behavior, thereby enabling the precise control and rational design of advanced materials with an enhanced NLO performance.

## Computational methods

All calculations were carried out using density functional theory (DFT) and time-dependent DFT (TD-DFT) methodologies, as implemented in the Gaussian 16 suite.^50^ The theoretical framework of this study utilized molecular structures derived from X-ray crystallography of the *o*-carboranyl compounds synthesized by You and collaborators.^49^ Calculations were executed using the CAM-B3LYP functional,^51^ a long-range corrected functional developed to handle the inaccuracies of the non-Coulomb part of the exchange functional at long distances. All calculations were conducted in a tetrahydrofuran solvent environment utilizing a polarizable continuum model (PCM), specifically through the integral equation formalism variant (IEFPCM),^52^ to obtain the geometrical structure inputs used for simulation of the static and dynamic components of the polarizability and the first- hyperpolarizability tensor within a solvent medium.

The average polarizability (*α*) and its anisotropy (Δ*α*) are defined through the following relations:^53^1
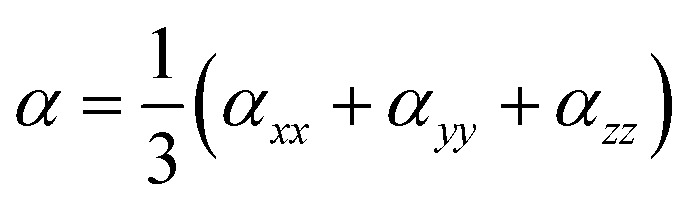
2
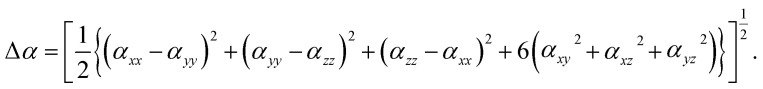


The first hyperpolarizability is outlined as follows:3
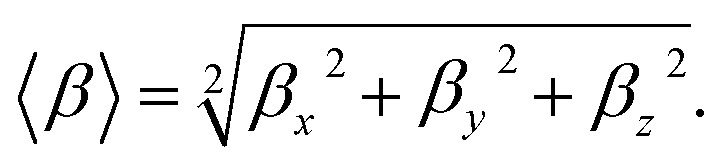


Also, *β*_vec_ may be defined as:4
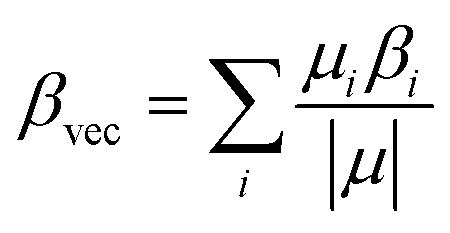
where the components *β*_*x*_, *β*_*y*_ and *β*_*z*_ are defined as:5
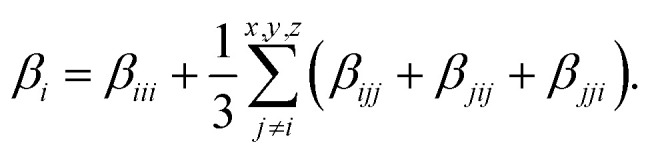


On the other hand, the hyper-Rayleigh scattering (HRS) method is employed for the detection of incoherent second harmonic generation (SHG) at frequency 2*ω*, induced by the incidence of a laser operating at frequency *ω*. This technique facilitates the determination of the first-order hyperpolarizability (*β*_HRS_) and its associated depolarization ratios (DR). These parameters can be quantified using the following analytical expressions:^54–57^6
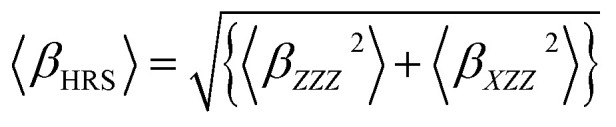
7
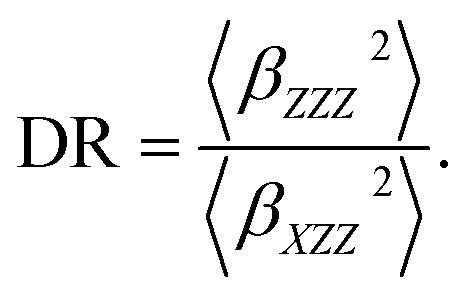
〈*β*_*ZZZ*_^2^〉 and 〈*β*_*XZZ*_^2^〉 can be decomposed into two symmetry-allowed contributions, namely, the dipolar (*J* = 1) and octupolar (*J* = 3) components of the first hyperpolarizability, respectively, which can be calculated as follows:^57^8
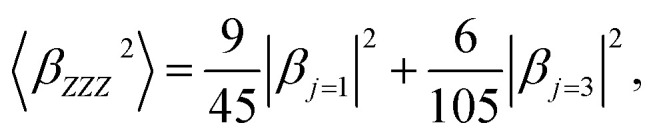
9
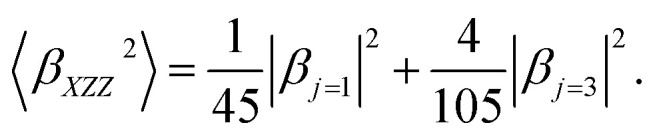


The two components can be evaluated as follows:10
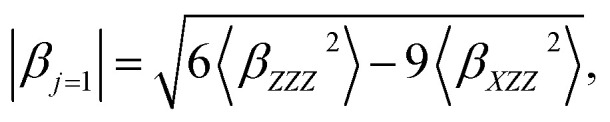
11
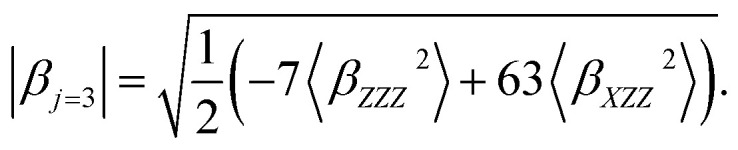


The relative dipolar and octupolar contributions to the first hyperpolarizability, denoted as *φ*_*J*=1_ and *φ*_*J*=3_, respectively, are defined as follows:12
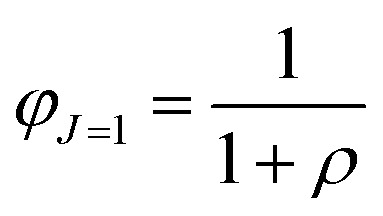
13
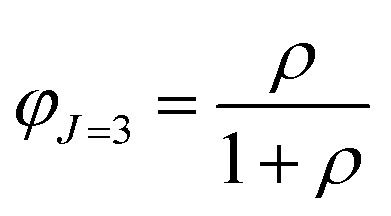
where *ρ* = |*β*_*j*=3_|/|*β*_*j*=1_|, clearly *φ*_*J*=1_ + *φ*_*J*=3_ = 1.

Additionally, the hole–electron distribution was analyzed to investigate the characteristics of electronic excitation within the compounds. The spatial distributions of holes and electrons are defined as follows:^58–60^14
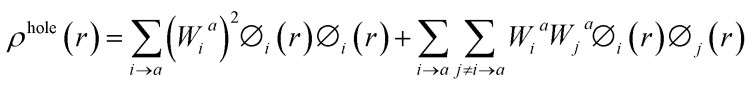
15
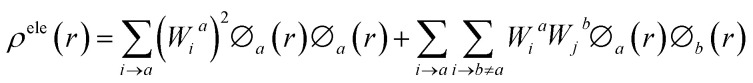
where *ϕ* represents the orbital wave function, *W* denotes the excitation coefficient, *i* and *j* refer to the indices of occupied orbitals, while *a* and *b* correspond to the indices of virtual orbitals.

The overlap distribution between the hole and electron can be mathematically represented as follows:^59,60^16
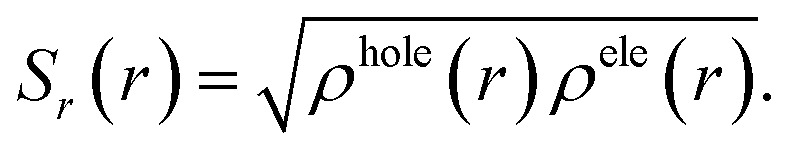


To quantify the degree of overlap between the hole and electron distributions, the *S*_*r*_ index is defined as follows:17
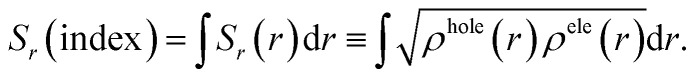


The overall charge transfer (CT) length is quantified by the *D*_index_, which is determined using the following expression:

18 *D*_index_ = [(*D*_*x*_)^2^ + (*D*_*y*_)^2^ + (*D*_*z*_)^2^]^1/2^

The charge transfer direction (*H*_CT_) can be calculated as follows:

19 *H*_CT_ = |*H*·*u*_CT_|

where *u*_CT_ is the unit vector in CT direction and *H*_index_ represents the spatial extent of the average distribution of holes and electrons.

The variations in the dipole moment of the excited state with respect to the ground state in the *X*, *Y* and *Z* directions can be simply calculated as follows:20
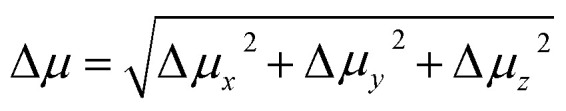
where, Δ*µ*_*x*_ = (*X*_ele_ − *X*_hole_), Δ*µ*_*y*_ = −(*Y*_ele_ − *Y*_hole_), and Δ*µ*_*Z*_ = −(*Z*_ele_ − *Z*_hole_).

## Results and discussion

### The effect of basis sets on the NLO responses

In contemporary computational chemistry research, DFT and TD-DFT have proven to be instrumental in the detailed description of the optical characteristics of compounds. Nevertheless, selecting an appropriate level of computational precision is crucial for accurately determining both linear and nonlinear optical properties. To this end, seven Pople basis sets, specifically 6-31++G(d,p), 6-31+G(d,p), 6-31++G, 6-31+G, 6-31G(d,p), 6-31G(d) and 6-311G(d), were chosen to discuss the influence of the basis sets on the electronic absorption wavelength and NLO responses by utilizing the CAM-B3LYP functional.

**Fig. 2:**
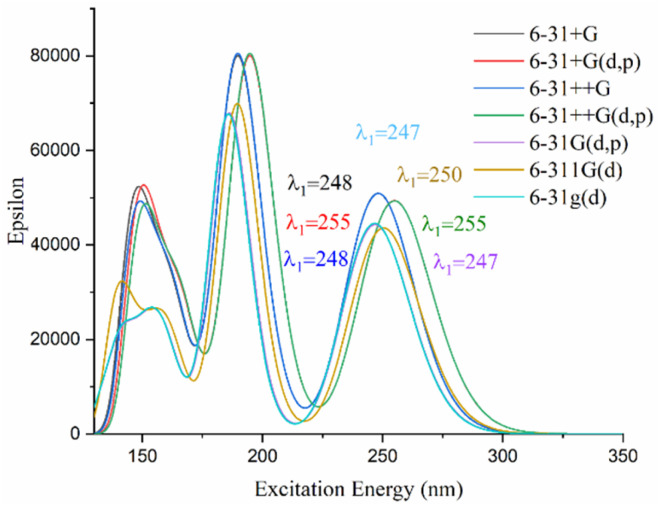
Simulated absorption spectrum of 1C.

The calculated principal absorption bands of 1C, as evaluated across different basis set levels, are illustrated in Fig. 2. Intriguingly, the variation in absorption wavelength (*λ*_1_) between the 6-31++G(d,p) and 6-31G(d,p) basis sets is approximately 8 nm. This finding indicates that the effect of the basis set size on the estimated absorption wavelength is negligible (Fig. 2).

The effect of the basis set on the NLO responses was tested for compound 1C. As can be seen in Table S2, the polarizability and anisotropy polarizability values change slightly with an increase in the number of basis functions. Overall, *α*(0,0) and Δ*α*(0,0) increase in the following order:

*α*(0,0): 6-31G(d) ≈ 6-31G(d,p) < 6-311G(d) < 6-31+G ≈ 6-31++G < 6-31+G(d,p) ≈ 6-31++G(d,p),

Δ*α*(0,0): 6-31++G ≈ 6-31+G < 6-31+G(d,p) = 6-31++G(d,p) < 6-311G(d) ≈ 6-31G(d) < 6-31G(d,p).

From this observation, it is apparent that incorporating diffuse functions leads to an increase in the *α*(0,0) value and a decrease in the Δ*α*(0,0) value, each by approximately 40 a.u. A secondary insight derived from these results is that the static first hyperpolarizabilities of 1C are significantly overestimated when the basis set excludes diffuse functions. This overestimation decreases in the following sequence: 6-31G(d,p), 6-31++G(d,p), 6-31+G(d,p) and (6-31++G) ≈(6-31+G), a trend that also holds for the anisotropy of polarizability. Going from a (+) diffuse to a (++) diffuse basis set results in a lower enhancement in both polarizability and first hyperpolarizability (Table S1). These studies show that including one or two diffuse functions results in almost unchanged values for polarizability and first hyperpolarizability. This occurs when the 6-31+G(d,p) or 6-31+G basis set is compared with 6-31++G(d,p) or 6-31++G, respectively. For instance, *β*_HRS_^∞^ (6-31++G) ≈ *β*_HRS_^∞^ (6-31+G) > *β*_HRS_^∞^ (6-31++G(d,p)) ≈ *β*_HRS_^∞^ (6-31+G(d,p)).

On the other hand, the calculated static first hyperpolarizability values obtained with polarization-type basis sets exhibit the following decreasing trend: *β*_HRS_^∞^ (6-311G(d)) > *β*_HRS_^∞^ (6-31G(d,p)) ≈ *β*_HRS_^∞^ (6-31G(d)). This observed trend indicates that the triple-split valence basis set 6-311G(d) yields the largest first hyperpolarizability, while the double-split sets 6-31G(d) and 6-31G(d,p) give smaller and nearly equivalent values.

For 1C, the 6-31G(d,p) and 6-31G(d) basis sets yield moderate first hyperpolarizability values, positioned between the higher values obtained with triple-split valence sets and the lower values from diffuse basis sets (Table S1). Previous works,^61–65^ including studies by You *et al.*,^49^ have demonstrated the effectiveness of the 6-31G(d,p) basis set in linear and nonlinear optical calculations, confirming its suitability for providing an optimal balance between accuracy and computational efficiency. Consequently, the 6-31G(d,p) basis set was chosen for this investigation, as it has proven reliable in determining both linear and nonlinear optical properties for similar compounds.

To verify that our computational approach can reproduce the experimental UV/vis behavior of the studied molecules, we compared the TD-CAM-B3LYP/6-31G(d,p) absorption (*λ*_abs_) and emission (*λ*_em_) wavelengths of compounds 1C–6C with their measured values. As shown in Table S2, the calculated *λ*_abs_ values follow the experimental trend very closely, with deviations of 6.8–9.3%, which is fully consistent with the expected accuracy of this functional. For the emission spectra, the calculated *λ*_em_ wavelengths show slightly larger deviations (11.57–12.96%), but still reproduce the ordering and relative evolution of the fluorescence bands across the series. Despite the experimental red-shift, the theoretical results capture the correct magnitude and progression of the optical transitions. Overall, these comparisons confirm that the CAM-B3LYP/6-31G(d,p) methodology provides a reliable description of the electronic excitations and excited-state properties of the targeted compounds.

### Geometrical study

The optimized geometries of the ground state (S_0_) and first excited (S_1_) states of *i*M, *i*H and *i*C (*i* = 1 to 9) in THF were obtained at the CAM-B3LYP/6-31G(d,p) level of theory. Based on the examination of the geometric parameters of the ground state (S_0_) summarized in Table 1, it is evident that there is outstanding concordance between the simulated and experimental data. In all the optimized molecular geometries (S_0_), the biphenyl entities, designated as Frag-2, manifested a uniform angular distortion of 37° (*φ*_1_), as listed in Table 1. On the other hand, the *φ*_2_ angle, which quantifies the exo–π-interaction magnitude between the cage framework, termed Frag-1, and the conjugated aromatic moiety, was observed to be perpendicular (∼90°) in the series of 1C–9C compounds. This angle approximated perpendicularity (∼82°) for the *o*-carborane compounds, with the notable exceptions of compounds 6H and 7H, wherein *φ*_2_ was recorded to be 30°. The analysis further reveals that the C1′–C2′ bond length in the trimethylsilyl-*o*-carborane compounds is elongated by approximately 0.046 Å compared to its counterparts in the H-*o*-carborane compounds (1H–9H). This elongation is attributed to the incorporation of the SiMe_3_ group within the *o*-carborane cage. Additionally, it is noteworthy that the computed C2–C3 bond length in the studied compounds is 1.418 Å, indicating that Frag-1 and Frag-3 do not influence the C2–C3 bond length within Frag-2.

**Table 1:** Calculated values from the ground- (S_0_) and first-excited-singlet- (S_1_) state optimized structures: dihedral angles between the rings Ph1 and Ph2 (*φ*_1_ = C1–C2–C3–C4) and between the C1′–C2′ bond axis in the *o*-carborane cage and Ph1 (*φ*_2_ = C1′–C2′–C6–C5), including the bond lengths for C2–C3, C6–C2′ and C1′–C2′ of the compounds 1–9. The experimental results reported for the *i*C compounds (values provided in brackets) are from ref. 49

	S_0_	S_1_	S_0_	S_1_	S_0_	S_1_	S_0_	S_1_	S_0_	S_1_
	C2–C3	C2–C3	C6–C2′	C6–C2′	C1′–C2′	C1′–C2′	*φ*_1_	*φ*_1_	*φ*_2_	*φ*_2_
1M	1.483	1.413	1.431	1.388	—	—	38	2.82	—	—
1H	1.483	1.401	1.509	1.481	1.649	1.707	38	0.094	81	91.74
1C	1.483	1.440	1.509 (1.506)	1.435	1.689 (1.709)	2.329	38 (42.6)	17.46	91 (77.2)	91.35
2M	1.483	1.414	1.431	1.387	—	—	37	3.173	—	—
2H	1.483	1.435	1.509	1.437	1.650	2.319	38	15.059	83	91.16
2C	1.483	1.436	1.509 (1.505)	1.439	1.690/1.710	2.336	38 (24.6)	16.21	92 (86)	89.28
3M	1.483	1.414	1.431	1.387	—	—	38	3.111	—	—
3H	1.483	1.437	1.509	1.437	1.649	2.319	38	15.70	81	91.328
3C	1.483	1.438	1.509 (1.506)	1.438	1.690 (1.708)	2.336	38 (19.0)	16.36	93 (87.9)	90.168
4M	1.483	1.412	1.431	1.389	—	—	36	2.60	—	—
4H	1.482	1.433	1.509	1.438	1.650	2.318	37	13.14	84	88.92
4C	1.482	1.434	1.509 (1.511)	1.439	1.690 (1.715)	2.335	37 (30.5)	14.54	92 (79.2)	89.80
5M	1.482	1.412	1.431	1.389	—	—	37	2.67	—	—
5H	1.482	1.433	1.509	1.437	1.650	2.317	37	12.43	83	88.92
5C	1.482	1.434	1.509 (1.504)	1.439	1.690 (1.697)	2.334	37 (36.9)	14.53	93 (83)	89.29
6M	1.481	1.412	1.430	1.391	—	—	36	2.47	—	—
6H	1.481	1.406	1.507	1.480	1.631	1.644	36	1.55	30	31.55
6C	1.481	1.431	1.509 (1.503)	1.441	1.691 (1.718)	2.336	36 (34.5)	13.28	92 (82.6)	90.424
7M	1.482	1.390	1.430	1.413	—	—	37	2.96	—	—
7H	1.481	1.406	1.507	1.479	1.632	1.643	37	2.29	30	30.86
7C	1.481	1.432	1.508	1.441	1.691	2.337	36	−13.69	92	90.07
8M	1.480	1.415	1.430	1.395	—	—	35	−3.071	—	—
8H	1.480	1.434	1.508	1.442	1.652	2.326	35	13.74	84	89.18
8C	1.480	1.433	1.508	1.445	1.692	2.341	35	−14.09	92	90.06
9M	1.479	1.414	1.430	1.397	—	—	34.17	2.89	—	—
9H	1.478	1.435	1.507	1.441	1.652	2.325	34.06	−13.20	84	91.12
9C	1.478	1.434	1.508	1.444	1.692	2.341	33.72	−14.81	91.78	89.90

The analysis of the optimized S_1_ excited-state structures of the title compounds reveals a slight contraction in the C2–C3 bond length compared to their corresponding ground states (S_0_). For the *i*H and *i*C compounds, this contraction is approximately 0.04 Å. Interestingly, in the 1H, 6H, and 7H derivatives, this contraction is nearly doubled, reaching approximately 0.07 Å. The *i*M compound exhibits an even more pronounced reduction in the C2–C3 bond length in the S_1_ state, measuring approximately 0.07 Å shorter than that in the ground state (Table 1). Moreover, a comparable trend is observed for the C6–C2′ bond. In the S_1_ excited state, this bond shortens by approximately 0.062–0.074 Å for the *i*H and *i*C compounds. However, the *i*M compound demonstrates a less pronounced contraction, with a reduction ranging from 0.033 to 0.044 Å. Distinct exceptions are noted in the 1H, 6H, 6M, and 7H compounds, where the C6–C2′ bond contracts by 0.028 Å, 0.027 Å, 0.017 Å, and 0.028 Å, respectively.

Additionally, the optimized structures of the *i*H and *i*C compounds demonstrate substantial differences in the C1′–C2′ bond length in the cage for the S_0_ and S_1_ optimized structures. Computational analysis reveals that in the S_1_ state, the C1′–C2′ bond length extends by approximately 0.6 Å compared to its value in the S0 state. However, distinct deviations are noted for the 1H, 6H, and 7H compounds, where the bond length increases by only 0.058 Å, 0.013 Å, and 0.011 Å, respectively.

On the other hand, the S_1_-optimized structure exhibits a noticeably distorted geometry in the biphenyl rings of the *o*-carboranyl compounds, with an observed dihedral angle (*φ*_1_) of approximately 13°–17°, except for 1H, 6H, and 7H, where *φ*_1_ = 0°, 1° and 2°, respectively. This is accompanied by an orthogonal arrangement between the C–C bond within the *o*-carborane cage and the biphenyl plane (*φ*_2_ ≈ 90°), contrasting sharply with the ground-state geometry, where *φ*_1_ measures 36°. This is with the exception of compounds 6H and 7H, which do not exhibit perpendicular structures, *φ*_2_ = 30°. According to this analysis, it can be concluded that the results obtained for the S1-optimized structures of the *o*-carboranyl compounds indicate that the C1′–C2′ bond distance and the angle *φ*_1_ are primary contributors to the emission properties.

### UV-vis and IFCT analyses (electron–hole analysis)

In this phase of our research, we focused on analyzing the UV-vis spectra of the title compounds. To accomplish this objective, we employed the TD-DFT method, a robust computational approach widely acknowledged for its effectiveness in scrutinizing the electronic properties of molecules. Furthermore, we conducted theoretical calculations for the compounds under investigation to determine their inter-fragment charge transfer (IFCT)^66^ properties. Tables S3–S7 in the SI provide comprehensive information regarding the spectroscopic attributes of the electronic transitions, encompassing absorption wavelengths, excitation energies, oscillator strengths, and significant contributions from each transition.

**Fig. 3:**
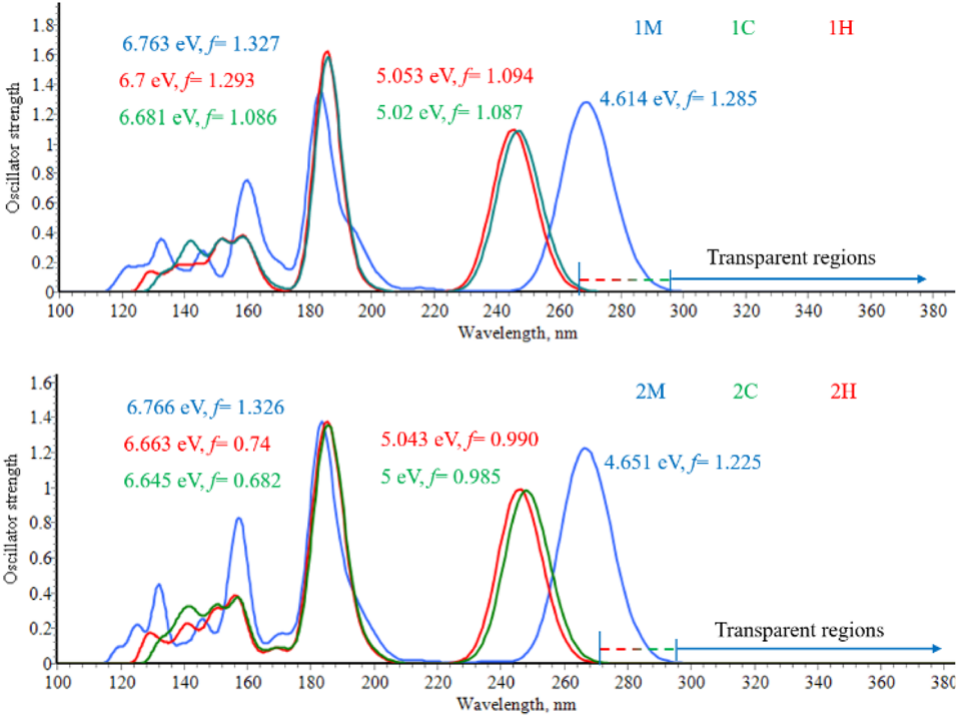
Simulated UV-vis absorption spectra in THF for the compounds 1 and 2 at the TD-CAM-B3LYP/6-31G(d,p)/IEFPCM level of theory.

The TD-DFT analysis indicates that the first-excited-state transition S_0_ → S_1_ in the *i*M molecules shifted to a longer wavelength compared to the corresponding transition in the *i*C and *i*H *o*-carboranyl compounds for *i* = 1 to 9. Furthermore, the absorption spectrum of the *i*C compounds closely resembles that of the *i*H compounds, as illustrated in Fig. 3 and S1 in the SI. All the series of compounds, *i*M, *i*H, and *i*C, exhibited two prominent absorption bands. The first band is centered within the wavelength range of 240–300 nm, while the second band is observed at approximately 185 nm (Table S4). Additionally, the lowest-energy electronic transitions (S_0_ → S_1_) of the *i*M compounds (*i* = 1–9) exhibit a systematic redshift of approximately 21 nm in their absorption maximum (*λ*_max_) relative to those of the corresponding *i*C and *i*H analogues (see Table S4). On the other hand, the title compounds show no absorption within the UV-vis range (300 nm to 700 nm) and the near-infrared (NIR) range (700 nm to 2500 nm), indicating their transparency throughout the UV-vis-NIR spectrum. This transparency, extending beyond 300 nm, positions these compounds as promising candidates for applications in UV-vis-NIR NLO materials.

By analyzing the electron–hole wave-function overlap integral (*S*_*r*_), the distance between the centroids of holes and electrons (*D*_index_), the variation in dipole moment relative to the ground state (Δ*µ*), the hole delocalization index (HDI), and the electron delocalization index (EDI) as descriptors of electron excitation types, it is possible to infer the nature of these transitions.^67–70^

**Fig. 4:**
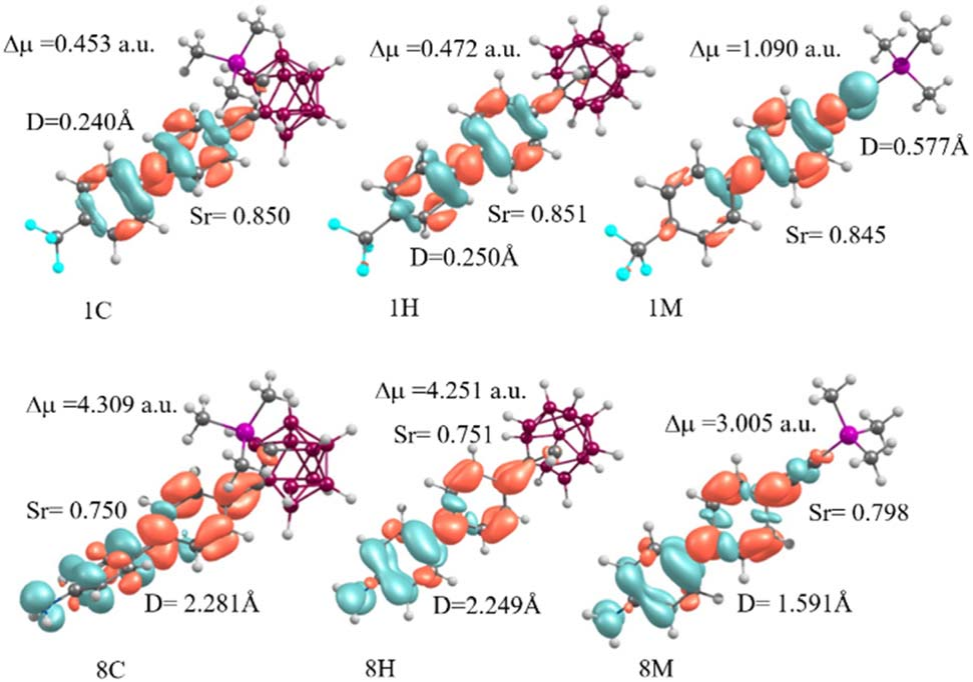
CDD analysis for the first excited states (with the largest oscillator strength) of the compounds 1 and 8. CDD was calculated as the difference between the corresponding excited state and the ground state of the considered system at the CAM-B3LYP/6-31G(d,p)/IEFPCM level of theory. Blue regions indicate negative electron density while orange regions correspond to positive electron density. The isosurface level is set to be 0.001807.

The TD-DFT calculations for the title compounds indicate that the low-energy transitions (S_0_ → S_1_) are predominantly characterized by an electronic excitation from the HOMO to the LUMO, with a 90% contribution (Table S4). As detailed in Table 2 (see also Table S5 in the SI), it is evident that the electron–hole wave-function overlap integral (*S*_r_) for all the first excited states (S_1_) is close to 1 (*S*_*r*_ > 0.7). The centroid distance between electrons and holes ranges from 0.2 to 1.2 Å for the *i*H, *i*M, and *i*C compounds (*i* = 1 to 5), while for compounds *i* = 6 to 8, *D* varies from 1.7 to 2.3 Å. The delocalization index (*H*) is approximately 3.4 Å for the *i*M molecules and 3.1 Å for the *i*H and *i*C compounds. The hole delocalization index (HDI) is slightly larger than the electron delocalization index (EDI) across all the compounds. The *t* index is negative and significantly less than zero, indicating the absence of charge separation. This suggests that the first excited state (S_1_) is a typical locally excited (LE) state. This conclusion is further supported by the analysis of the charge density difference (CDD) between the ground and excited states, as shown in Fig. 4 and S2 in the SI.^59^

**Table 2:** Calculated overlap (*S*_*r*_), *D*_index_ (Å), *H*_index_ (Å), variation in dipole moment with respect to the ground state (Δ*µ*, a.u.), hole delocalization index (HDI), electron delocalization index (EDI), oscillator strength (f), excitation energy (Δ*E*, eV), and Coulomb attractive energy (*E*_CA_, eV) for the *i*M, *i*H and *i*C compounds in the first excited state (S_0_ → S_1_) at the CAM-B3LYP/6-31G(d,p)/IEFPCM level of theory

	*S*_*r*_	*D*	*H*	Δ*µ*	HDI	EDI	Δ*E*	*f*	*E*_CA_
1M	0.845	0.577	3.417	1.090	6.84	6.28	4.613	1.557	5.274
1H	0.851	0.250	3.072	0.472	6.91	6.57	5.052	1.352	5.572
1C	0.850	0.240	3.114	0.453	6.86	6.50	5.02	1.15	5.450
2M	0.847	0.193	3.380	0.364	6.63	6.64	4.65	1.507	5.347
2H	0.830	1.091	3.021	2.061	6.96	6.95	5.043	1.225	5.540
2C	0.828	1.113	3.073	2.103	6.90	6.86	5	1.111	5.392
3M	0.849	0.048	3.352	0.091	6.68	6.57	4.655	1.233	5.368
3H	0.839	0.877	2.997	1.656	6.90	6.82	5.065	0.994	5.587
3C	0.837	0.899	3.045	1.698	6.85	6.74	5.0238	1.103	5.441
4M	0.8455	0.290	3.438	0.548	6.44	6.58	4.6006	1.608	5.274
4H	0.826	1.160	3.073	2.191	6.88	6.81	4.9501	1.299	5.458
4C	0.824	1.182	3.113	2.233	6.81	6.73	4.9093	1.1787	5.320
5M	0.846	0.291	3.465	0.549	6.42	6.56	4.5915	1.674	5.237
5H	0.827	1.173	3.095	2.217	6.83	6.80	4.9381	1.339	5.417
5C	0.8253	1.201	3.142	2.269	6.78	6.69	4.9074	1.216	5.290
6M	0.827	0.935	3.514	1.767	6.33	6.67	4.501	1.651	5.152
6H	0.794	1.750	3.147	3.306	7.30	6.87	4.791	1.431	5.235
6C	0.788	1.811	3.137	3.421	7.26	6.85	4.731	1.223	5.156
7M	0.828	0.858	3.475	1.621	6.35	6.71	4.537	1.589	5.212
7H	0.795	1.714	3.090	3.238	7.20	7.00	4.836	1.367	5.322
7C	0.790	1.754	3.096	3.314	7.21	6.92	4.774	1.178	5.224
8M	0.798	1.591	3.536	3.005	6.47	6.70	4.355	1.694	5.055
8H	0.751	2.249	3.11	4.251	7.67	6.98	4.5337	1.356	5.158
8C	0.750	2.281	3.162	4.309	7.57	6.84	4.4982	1.235	4.996
9M	0.769	2.165	3.602	4.090	7.20	6.56	4.089	1.798	4.808
9H	0.731	2.551	3.231	4.821	8.33	6.73	4.243	1.4319	4.873
9C	0.723	2.648	3.222	5.003	8.36	6.70	4.187	1.296	4.764

It is important to emphasize that the *i*M series exhibits the highest *S*_*r*_ values for weak donors (3M: 0.849) but converges toward values comparable to the *i*H and *i*C series for strong donors (9M: 0.769, 9H: 0.731, and 9C: 0.723). TMS functionalization (*i*C) slightly reduces *S*_*r*_ compared to *i*H, with a maximum difference of −1.1% for compounds 9. This decrease in *S*_*r*_ with increasing donor strength confirms the reduced hole–electron overlap, which is favorable for charge separation. The *D*_index_ increases drastically with donor strength, establishing the hierarchy *i*C > *i*H > *i*M, except for the –CF_3_ substituent, where the order is the opposite (1M: 0.577 Å > 1H: 0.250 Å > 1C: 0.240 Å). This atypical behavior arises from the strongly electron-withdrawing character of –CF_3_, which reverses the electron flow and alters the charge distribution. In the *i*M series, the *D*_index_ varies from 0.048 Å (3M, –H) to 2.165 Å (9M, –NMe_2_), corresponding to a ∼45-fold increase. The *i*H and *i*C series show higher values for donor substituents, reaching 2.551 Å for 9H and 2.648 Å for 9C (+3.8% *vs.* 9H and +22% *vs.* 9M). Therefore, TMS functionalization enhances spatial separation, with the effect being maximized for strong donors. The dipole moment variation (Δ*µ*) follows the same trend as *D*_index_, confirming the proportional relationship between spatial separation and dipole moment variation. In the *i*C series, Δ*µ* increases from 1.698 a.u. (3C) to 5.003 a.u. (9C), corresponding to a ∼3-fold enhancement. The hierarchy *i*C > *i*H > *i*M is maintained, as follows: 9C (5.003 a.u.) > 9H (4.821 a.u.) > 9M (4.090 a.u.). TMS functionalization further amplifies Δ*µ* by +3.8% (9C *vs.* 9H) and +22% (9C *vs.* 9M), demonstrating the synergistic effect between the *o*-carborane cage and TMS substitution.

Generally, the *H*_index_ follows the reverse trend (*i*M > *i*C > *i*H) with the exception of compounds 6 and 9, where *i*H > *i*C. The values remain within a moderate range (from 2.997 (3H) to 3.602 (9M)) corresponding to an increase of roughly 20%. This trend suggests that the *i*H and *i*C series confine the electronic distributions more strongly while maximizing vectoral charge separation.

The coulombic energy (*E*_CA_), which quantifies the electrostatic attraction between the hole and the electron, systematically decreases with donor strength and follows the hierarchy *i*C < *i*H < *i*M. For weak donors, the *i*M series presents moderate values (3M: 5.368 eV), while the *i*H and *i*C series display higher values (3H: 5.587 eV and 3C: 5.441 eV). However, with strong donors, a remarkable inversion occurs, as follows: 9C (4.764 eV) < 9M (4.808 eV) < 9H (4.873 eV). TMS functionalization consistently reduces the E_CA_ (9C *vs.* 9H: −2.2%), confirming its role in optimizing charge separation.

Our results show that the integration of the *o*-carborane cage (*i*H and *i*C) significantly enhances the *D*_index_ and Δ*µ*, while concurrently reducing the *S*_*r*_ overlap and the *E*_CA_ relative to the *i*M series (*i* = 2–9). These effects are further amplified when strong electron-donating groups, such as –NH_2_ and –NMe_2_, are incorporated. This observation indicates that the combination of *o*-carborane with TMS substitution constitutes a synergistic strategy for enhancing spatial charge separation and promoting dipolar asymmetry. The atypical behavior of compounds 1M, 1H, and 1C can be attributed to the presence of the strongly electron-withdrawing –CF_3_ group,^71^ which reverses the charge-transfer direction typically observed with donor substituents. This highlights the critical role of substituent electronic character in modulating charge-transfer properties.

The hole–electron descriptors (Sr, HDI, and EDI) for the second absorption band at around 180 nm, as presented in Table S5, show no significant difference from those of the S_1_ transition, with the *D*_index_ values following the same trend. The complementary CDD analysis (see Fig. S2) confirms that all the excitations at around 180 nm correspond to local charge transfer within the biphenyl fragment.

**Fig. 5:**
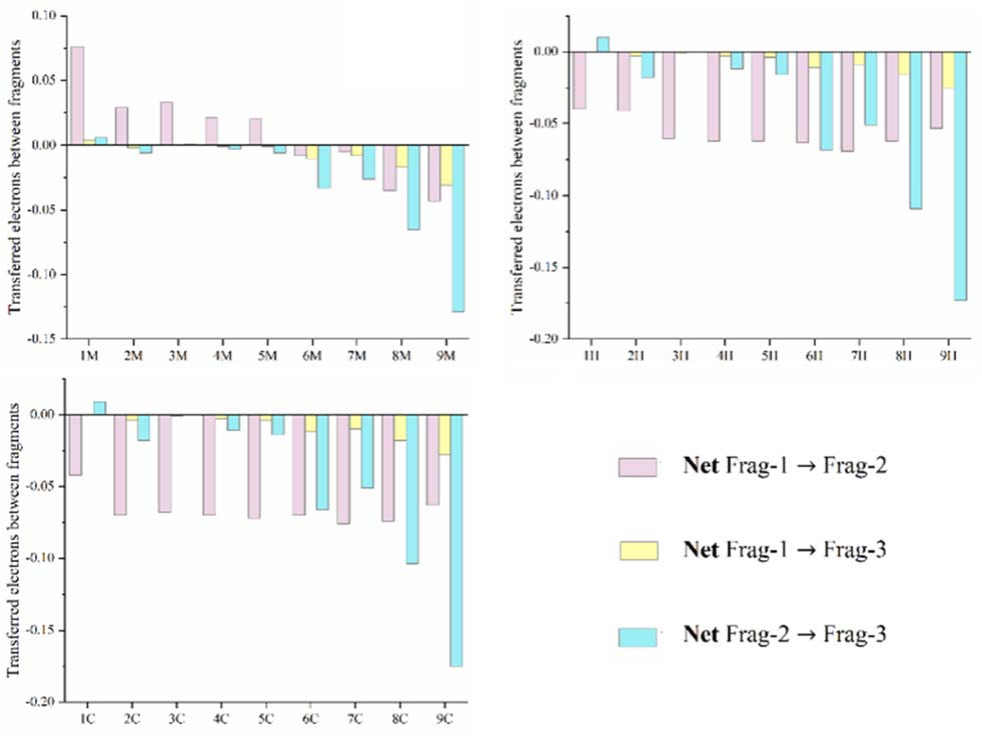
Transferred electrons between fragments, calculated for the first excited states of the *i*M, *i*H and *i*C compounds at the CAM-B3LYP/6-31G(d,p)/IEFPCM level of theory in THF.

In this study, the IFCT approach was employed to compute the net electron transfer between various segments during electronic excitation.^58,59,72^ This is achieved by analyzing the difference between electron-donating and electron-accepting contributions in each segment. Additionally, calculations were performed to determine the charge transfer percentage (CT%) and its complement, the local excitation percentage (LE%). The results of these calculations are presented in Fig. 5 and Table S6.

The analysis of the S_0_ → S_1_ excitation reveals that the percentages of local excitation (LE) significantly exceed those of charge transfer (CT) across the *i*M, *i*H, and *i*C compounds, indicating that LE predominantly characterizes the transitions. The contribution from CT follows the trend *i*M > *i*H ≈ *i*C, with the *i*M compounds exhibiting relatively consistent values (∼40% CT and ∼60% LE) independent of the R group. In contrast, the R substituent has a more pronounced effect on the CT character in the *i*H and *i*C compounds. Among the substituents, –NMe_2_ and –NH_2_ lead to the highest CT percentages, followed by –OMe, –OH, –CMe_3_, –Me, and –H. Taking the *i*H compounds as an example, CT (%) increases in the following order: 9H (37%) > 8H (29%) > 6H (26%) > 7H (24%) ≈ 2H (24%) > 5H (20%) > 4H (19%) > 3H (17%) > 1H (9%), highlighting the significant influence of the R substituent on the CT characteristics.

The interfragment analysis (Table S6) reveals that intrafragment electron redistribution on the biphenyl is moderate for the *i*M series (0.52–0.59) and more pronounced for the *i*H and *i*C series (0.60–0.83), with the trend of *i*H > *i*C > *i*M. Notably, these values decrease with an increasing donor strength of the substituents. The variation in population numbers supports these trends, as follows: in the *i*M series, biphenyl gains electrons, while acetylene shows variable behavior. In contrast, the *i*H and *i*C series consistently exhibit electron gain by the *o*-carborane, with strong donors such as –NMe_2_ efficiently donating electrons, as seen in 9H (−0.198). The fragment contributions further support these findings, as follows: Frag-2 (biphenyl) contributes 68–75% to the hole distribution in *i*M *versus* 71–91% in *i*H and *i*C, and 75–78% to the electron distribution in *i*M compared to ∼85% in *i*H and *i*C. These results highlight the optimization of charge transfer (CT) in the *o*-carborane-based architectures by minimizing local electronic reorganization, emphasizing the role of both the *o*-carborane cage and the R group in controlling electronic distribution.

In the 1H[1C], 2H[2C], 3H[3C], 4H[4C] and 5H[5C] compounds, Frag-2 donates 0.039[0.042], 0.041[0.069], 0.059[0.068], 0.062[0.070] and 0.061[0.071] electrons to Frag-1, respectively. For the *i*M, *i*H, and *i*C non-centrosymmetric molecular series (*i* = 1 to 5), the S_0_ → S_1_ excitation results in minimal net electron transfer between fragments 1 and 3, and between fragments 2 and 3 in the *i*H series. The net electron transfer in the *i*C compounds is slightly larger than that in the *i*H compounds, likely due to the influence of the SiMe_3_ substituent on the *o*-carborane cage. In the *i*M compounds (*i* = 6–9), electron transfer from Frag-2 to Frag-1 varies, as follows: 6M (0.007), 7M (0.005), 8M (0.035), and 9M (0.042). The Frag-3 → Frag-2 transfer also varies with the R group, as follows: –OH (0.033), –OMe (0.026), –NH_2_ (0.064), and –NMe_2_ (0.129), with –NMe_2_ showing the largest transfer due to its strong electron-donating nature (Table S7). For the *i*H[*i*C] compounds (*i* = 6–9), electron transfer occurs *via* dual pathways, as follows: Frag-2 → Frag-1 and Frag-3 → Frag-2. The Frag-2 → Frag-1 transfers are 0.063[0.069], 0.068[0.075], 0.061[0.073] and 0.053[0.062] for 6H[6C], 7H[7C], 8H[8C], and 9H[9C], respectively. The Frag-3 → Frag-2 donations increase with donor strength, as follows: 0.068[0.066] for –OMe, 0.050[0.051] for –OH, 0.109[0.103] for –NH_2_, and 0.173[0.175] for –NMe_2_. This dual electron flow toward Frag-1 highlights a cooperative mechanism where strong donors enhance charge transfer through the biphenyl spacer to the *o*-carborane acceptor.

### Stokes shift

**Fig. 6:**
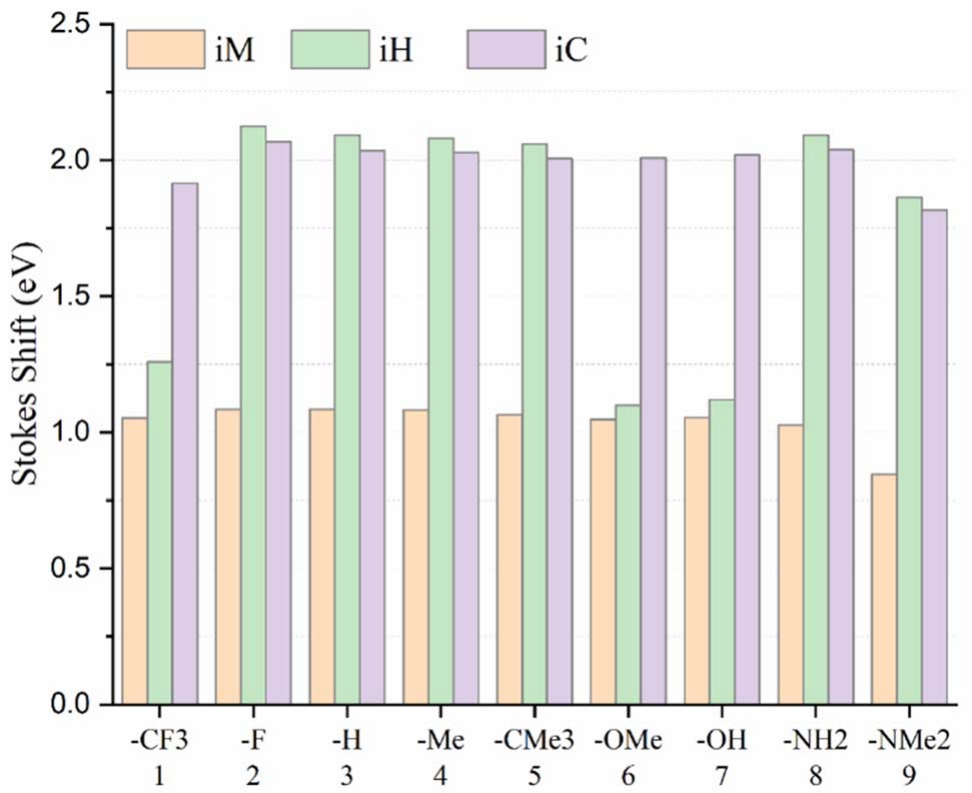
Variation in Stokes shift (eV) as a function of substituent nature for the *i*M, *i*H, and *i*C compounds.

The analysis of the Stokes shift variations across the *i*M, *i*H, and *i*C compounds, as depicted in Fig. 6, reveals distinct structure–property relationships governed by both the geometry and the electronic nature of the substituents. The *i*M compounds consistently display the smallest Stokes shifts (typically <1.09 eV), indicating limited excited-state relaxation and a weak ICT character. In contrast, the *i*H and *i*C series exhibit significantly larger Stokes shifts (ranging from ∼1.1 eV to 2.1 eV), reflecting enhanced ICT and substantial excited-state reorganization. However, an exception is observed for 6H (R = –OMe) and 7H (R = –OMe), which exhibit unusually low Stokes shifts, which are even comparable to or lower than those of the *i*M series. This deviation in 6H and 7H can be ascribed to the dihedral angle *φ*_2_ and the ground-state π-conjugation specific to the *i*H compounds bearing oxygen donors. In these molecules, the oxygen lone pair (n) engages in conjugation with the π system of the biphenyl rings (*φ*_1_ ≈ 30°), which in turn reduces the dihedral angle between the *o*-carborane cage and the biphenyl to *φ*_2_ ≈ 30°. This partial coplanarity enhances S_0_ conjugation, thereby minimizing the structural and electronic reorganization required upon excitation, resulting in a smaller geometry relaxation and a markedly reduced Stokes shift. This deviation is absent in the *i*C analogues because the bulky TMS group on the cage enforces orthogonality (*φ*_2_ ≈ 90°) through steric hindrance, suppressing n(O)-induced planarization, maintaining donor–acceptor decoupling in S_0_, and enabling a larger ICT and geometry relaxation, thus restoring the large Stokes shifts for –OMe and –OH. In the *i*M compounds, the absence of a strong cage-mediated ICT pathway leads to uniformly small and relatively constant shifts across the substituents. On the other hand, nitrogen donors (–NH_2_ and –NMe_2_), despite possessing lone pairs, do not induce the same *φ*_2_ collapse in *i*H due to differences in their lone-pair energy, orbital orientation, and steric effects; they preserve greater donor–acceptor separation in S_0_ and thus undergo more pronounced ICT and relaxation upon excitation, yielding large Stokes shifts.

### Linear and nonlinear optical parameters

**Fig. 7:**
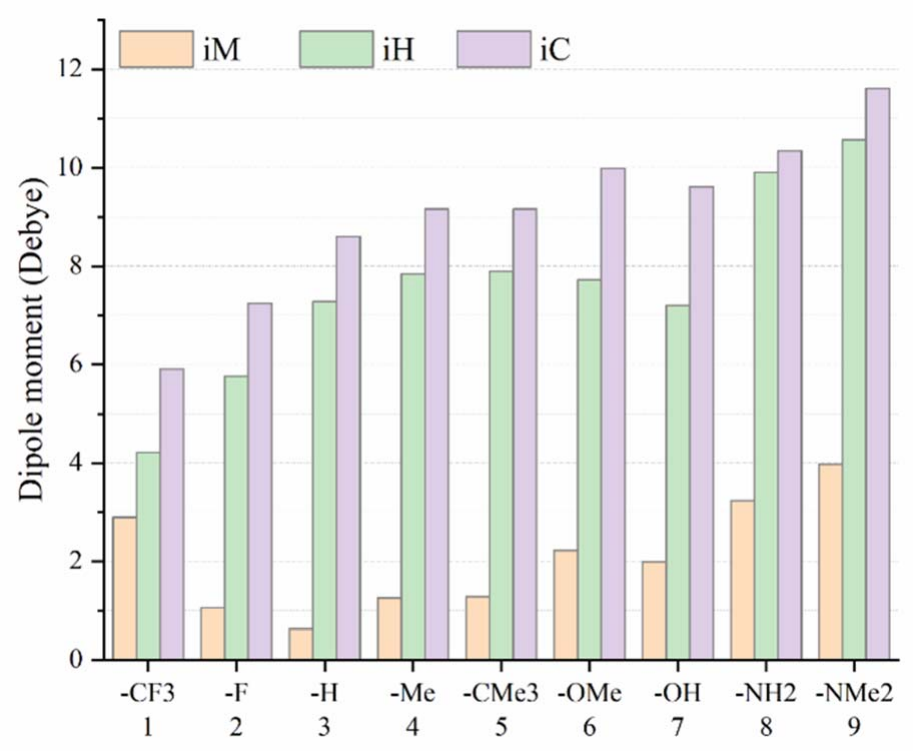
Variation in molecular dipole moment as a function of substituents (*i* = 1–9) for the *i*M, *i*H, and *i*C compounds.

The calculated static isotropic polarizability (*α*), polarizability anisotropy (Δ*α*), first hyperpolarizability (*β*_vec_ and *β*_0_), hyper-Rayleigh scattering (*β*_HRS_), depolarization ratio (DR), dipolar contribution to the beta (*φ*_*J*=1_) values and octupolar contribution to the beta (*φ*_*J*=3_) values of the title compounds (Fig. 1) are presented in Table 3 and Fig. 7.

**Table 3:** Static isotropic polarizability (*α*), polarizability anisotropy (Δ*α*), first hyperpolarizability (*β*_vec_ and *β*_0_), hyper-Rayleigh scattering (*β*_HRS_), depolarization ratio (DR), dipolar contribution to beta (*φ*_*J*=1_) and octupolar contribution to beta (*φ*_*J*=3_) calculated at the CAM-B3LYP/6-31G(d,p) level of theory in THF solvent (values in a.u.)

	*α*	Δ*α*	*β*_0_	*β*_vec_	*β*_HRS_	DR	*ϕ*_*J*=1_	*ϕ*_*J*=3_
1M	273	272	415	409	166	5.685	0.595	0.405
1H	327	217	850	637	329	6.546	0.652	0.348
1C	394	203	828	545	322	6.341	0.639	0.361
2M	258	260	1255	−1254	590	3.555	0.446	0.554
2H	313	208	2005	1740	830	4.985	0.550	0.450
2C	381	194	2041	1561	842	5.069	0.555	0.445
3M	258	260	673	672	341	3.090	0.404	0.596
3H	313	208	1482	1355	603	5.299	0.570	0.430
3C	381	194	1493	1252	605	5.388	0.576	0.424
4M	276	280	1444	1444	680	3.556	0.446	0.554
4H	331	227	2188	2033	915	4.832	0.539	0.461
4C	398	213	2236	1918	930	4.919	0.545	0.455
5M	318	295	1378	1377	617	3.991	0.480	0.520
5H	372	240	2172	2021	884	5.318	0.571	0.429
5C	440	227	2200	1887	892	5.367	0.575	0.425
6M	283	294	3085	2205	1364	4.116	0.489	0.511
6H	337	238	3332	3299	1394	4.824	0.539	0.461
6C	406	228	3744	3090	1564	4.855	0.541	0.459
7M	267	280	2836	1522	1254	4.121	0.490	0.510
7H	321	224	3080	3056	1283	4.899	0.544	0.456
7C	390	214	3471	2758	1443	4.928	0.546	0.454
8M	278	306	5094	4675	2202	4.380	0.508	0.492
8H	333	253	5440	4958	2278	4.816	0.538	0.462
8C	401	240	5627	5260	2349	4.869	0.542	0.458
9M	314	339	7364	7335	3179	4.395	0.509	0.491
9H	369	286	7666	7336	3235	4.700	0.530	0.470
9C	437	274	7902	7316	3326	4.741	0.533	0.467

### Dipole moments and polarizability

Fig. 7 presents the ground-state dipole moments for the three molecular families, *i*M, *i*H, and *i*C, as a function of substituent (*i* = 1–9). For each substituent, the dipole moment increases systematically in the order of *i*M < *i*H < *i*C, indicating that the *i*C compounds produce the strongest permanent polarization, the *i*H family shows intermediate values, and the *i*M series displays the weakest dipolar character. Across the substituent series, the dipole moment follows a clear monotonic trend consistent with donor strength. Electron-withdrawing groups (–CF_3_ and –F) yield the lowest dipole moments, followed by –H, –Me, –CMe_3_, –OMe, and –OH, while the strongest electron-donating groups (–NH_2_ and –NMe_2_) produce the largest dipole moments, reaching values up to ∼12 debye for 9C. This ordering reflects the progressive enhancement in intramolecular charge separation as the donor ability increases. Furthermore, analysis of the dipole-moment components shows that *µ*_*y*_ ≈ *µ*_*z*_ ≈ 0 for all the molecules, whereas *µ*_*x*_ is distinctly non-zero, demonstrating that the permanent dipole is strictly aligned along the longitudinal molecular axis. This confirms that the substituent-induced charge redistribution is strongly oriented along the *x*-direction of these donor–acceptor dyads.

For the *i*M series of compounds (*i* = 1 to 9), which incorporate a trimethylsilylacetylene acceptor, the calculated average polarizability (*α*) values range from 258 to 318 a.u. Upon substitution with the *o*-carborane unit in the *i*H and *i*C series, a consistent enhancement in polarizability is observed. In particular, the *i*C compounds exhibit significantly higher (*α*) values compared to both their *i*H and *i*M analogs, reflecting the strong electron-accepting nature and high electronic delocalization capacity of the *o*-carborane cage in combination with the extended π-conjugation. The highest polarizability is found in compound 5C, reaching 440 a.u., followed closely by 9C (437 a.u.), 7C (406 a.u.), and 8C (401 a.u.). The corresponding *i*H derivatives show intermediate values (369 a.u. for 9H, 333 a.u. for 8H, and 321 a.u. for 7H), whereas their *i*M counterparts yield the lowest polarizabilities of 314 a.u. (9M), 278 a.u. (8M), and 267 a.u. (7M), respectively. This trend highlights the synergistic role of both π-extension and electron-rich donor groups in enhancing the electronic cloud responsiveness. Notably, the increase in isotropic polarizability (*α*) closely parallels the trend observed in the computed dipole moments, underscoring the role of molecular asymmetry and charge-transfer character in governing the overall electronic response. In contrast, the polarizability anisotropy (Δ*α*) exhibits inverse behavior, where the *i*M derivatives display the largest anisotropy, while the *i*C derivatives show the smallest. This opposite trend indicates that, although the *i*C family possesses both higher polarizability and larger dipole moments, its electronic density is redistributed more uniformly, resulting in a comparatively isotropic response to an external electric field.

### The static first hyperpolarizability

**Fig. 8:**
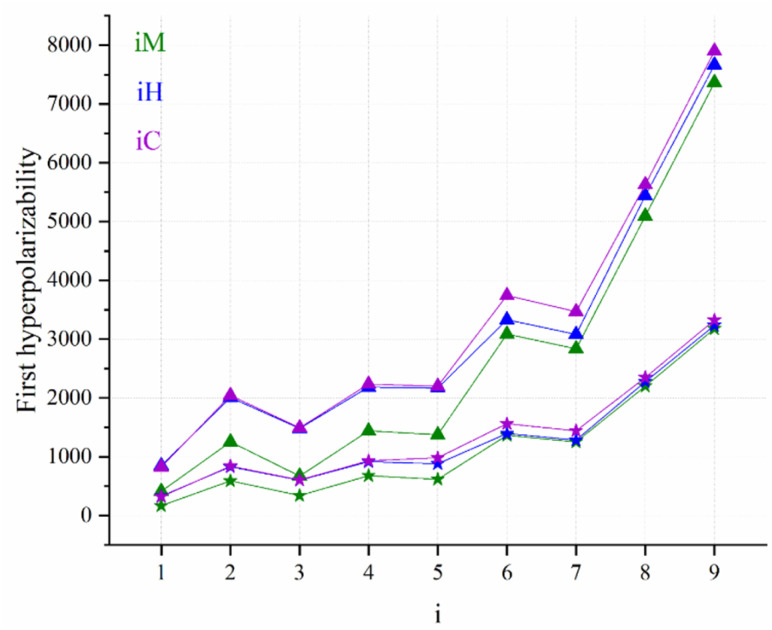
Static first hyperpolarizability (triangles: *β*(0;0,0) and stars: *β*^0^_HRS_) calculated for the compounds *i*M, *i*H and *i*C (*i* = 0 to 9) at the CAM-B3LYP/6-31G(d,p)/IEFPCM level of theory in THF.

Regarding the static first hyperpolarizability, the title compounds exhibit a consistent and parallel trend for both *β*(0;0,0) and *β*^0^_HRS_, with a near-perfect correlation between these parameters (*R*^2^ = 0.999; see Fig. S3). Notably, the *i*C compounds display the highest values for both *β*(0;0,0) and *β*^0^_HRS_ compared to their *i*H and *i*M counterparts (see Fig. 8). For example, in the 1C–5C series, the static first hyperpolarizabilities are elevated by approximately 100%, 63%, 122%, 55%, and 60% relative to their corresponding *i*M counterparts (1M–5M), respectively. A more moderate increase is observed for compounds 6C and 7C, with *β*(0;0,0) values approximately 21% higher than those of 6M and 7M, respectively. In the case of 8C and 9C, the enhancement is more modest at around 9% over 8M and 9M.

These results clearly indicate that substitution of the –C≡C–SiMe_3_ fragment by the *o*-carborane cage significantly enhances the second-order NLO response of the investigated molecules. Furthermore, a comparison between the *i*C and *i*H series reveals that *β*(0;0,0) for the *i*C compounds is generally ∼2% higher than that of their *i*H analogues, suggesting that the additional trimethylsilyl (TMS) group on the *o*-carborane cage exerts only a marginal influence on the first hyperpolarizability. However, notable exceptions are observed for compounds 6C and 7C, where their *β*(0;0,0) values increase by approximately 12% relative to that of 6H and 7H, respectively. This deviation can be attributed to geometric factors, particularly the *φ*_2_ angle, which adopts a value of ∼30° for R = −OMe and –OH substituents. This analysis indicates that the incorporation of a bulky trimethylsilyl (TMS) group into the cage structure has a minimal effect on the molecular hyperpolarizability (*β*(0;0,0) and *β*^0^_HRS_) of the *i*C compounds (6C and 7C are exceptions).

An in-depth analysis of the static first hyperpolarizability parameters (*β*(0;0,0) and *β*^0^_HRS_) across the *i*M, *i*H, and *i*C non-centrosymmetric molecular series reveals a pronounced dependence on the electronic nature of the R substituent at the donor site. Among the studied molecules, compound 1M, featuring a strong electron-withdrawing –CF_3_ group, exhibits the lowest first hyperpolarizability values (*β*(0;0,0) = 828 a.u. and *β*^0^_HRS_ = 322 a.u.). This trend persists consistently in the *i*H and *i*C analogues, suggesting that the –CF_3_ substituent severely limits the efficiency of intramolecular charge transfer (ICT), thereby diminishing the overall NLO response.

Progressive substitution of –CF_3_ with less electron-withdrawing or electron-donating groups results in substantial enhancements in first hyperpolarizability, following the trend of –H (3) < –F (2) < –Me (4) < –CMe_3_ (5) < –OH (7) < –OMe (6) < –NH_2_ (8) < –NMe_2_ (9). Across the *i*M, *i*H, and *i*C families, the compounds with R = –OMe, –OH, –NH_2_, and –NMe_2_ exhibit markedly higher *β* values, reflecting the superior electron-donating capability of these substituents, which facilitates stronger ICT from the donor to acceptor moieties through the π-conjugated bridge.

Quantitatively, the *β*(0;0,0) values for compounds *i* = 2–7 are typically 2–5-times larger than those of compound 1 (R = –CF_3_), while 8M and 9M show enhancements by factors of ∼12 and ∼18, respectively, relative to those of 1M. Similar enhancements are observed in compounds 8-9C and 8-9H, which exhibit first hyperpolarizabilities approximately 7- and 10-times greater than those of their corresponding 1C and 1H analogs, respectively. These results clearly demonstrate that electron-donating substituents significantly enhance the NLO response by increasing the electron density at the donor end, thereby promoting polarization across the D–A framework.

The diminished *β* values in the –CF_3_-substituted compounds can be ascribed to the strong electron-withdrawing character of the –CF_3_ group, which depletes electron density from the donor fragment and suppresses ICT in the A–A configuration. Conversely, electron-donating groups such as –NH_2_ and –NMe_2_ enable efficient push–pull electronic interactions in the D–A configuration, resulting in enhanced charge delocalization and elevated first hyperpolarizability. This substitution-driven modulation of electronic distribution highlights a powerful design strategy for tuning the NLO properties of conjugated molecular systems.

On the other hand, the *β*_vec_ analysis reveals a hierarchy of *i*H > *i*C > *i*M, with maximum values of 7316–7336 a.u. for the NMe_2_ series. The *o*-carborane architectures significantly enhance the vectoral first hyperpolarizability compared to the linear acetylene fragment, while the naked *o*-carborane (*i*H) slightly outperforms the TMS analogue (*i*C), suggesting that the TMS group partially attenuates the directional charge transfer response. This parameter represents the projection of the first hyperpolarizability tensor onto the dipole moment *µ*, where positive values indicate alignment with the permanent dipole (angle <90°) and negative values reflect the antiparallel orientation typical of inverted donor–acceptor polarity.

Furthermore, the analysis of the depolarization ratio combined with the dipolar–octupolar decomposition of first hyperpolarizability (Table 3) shows that only the first members of each series (1M, 1H, and 1C) display a pronounced dipolar nonlinear response, characterized by high DR values (5.685–6.546) and dominant dipolar contributions (*φ*_*J*=1_ > 0.59). In contrast, derivatives 2M–5M exhibit lower DR values (3.090–3.991) together with *φ*_*J*=3_ > *φ*_*J*=1_, indicating an octupolar-dominated response, with 3M being the most octupolar compound in the dataset (DR = 3.090). For the *i*H and *i*C families with *i* ≥ 2, the NLO behavior shifts toward a dipolar configuration, as reflected by their DR values close to 5 and moderately dominant dipolar contributions (*φ*_*J*=1_ ≈ 0.53–0.58). It is also noteworthy that 2H (4.985) and 2C (5.069) exhibit DR values essentially coincident with the dipolar reference (DR ≈ 5), a feature typically associated with ideal dipolar systems.

Urea is widely regarded as a prototypical reference compound in the study of second-order nonlinear optical responses and is frequently utilized as a benchmark for comparative analysis.^73^ In the present work, the first hyperpolarizabilities (*β*) of the *i*M, *i*H, and *i*C series were compared with that of urea (*β*_HRS_^∞^ = 38 a.u.).^74^ The comparison reveals that all the investigated derivatives exhibit *β*_HRS_^∞^ values that are significantly larger than that of urea. For instance, the first hyperpolarizabilities of 5M, 5H, and 5C are approximately 16-, 23-, and 24-times greater, respectively, than that of urea. This highlights the markedly enhanced NLO responses of the *i*M, *i*H, and *i*C derivatives relative to the standard urea molecule, emphasizing the superior nonlinear optical characteristics of the title compounds. On the other hand, a quantitative comparison with the fullerene-based benchmarks reported by Muhammad *et al.*^24^ and Fouejio *et al.*^25^ reveals the pronounced superiority of the *o*-carborane derivatives investigated in this study. For instance, the first hyperpolarizability of title compound 9C surpasses those of compounds {2 and 2c}^24^ and {fC_60_ }^25^ by multiplicative factors of 4, 37, and 20, respectively. These substantial enhancements demonstrate that the *o*-carborane cage furnishes a far more efficient three-dimensional electronic environment for intramolecular charge redistribution than the fullerene frameworks typically employed in nonlinear-optical design.

### Frequency dependence

**Fig. 9:**
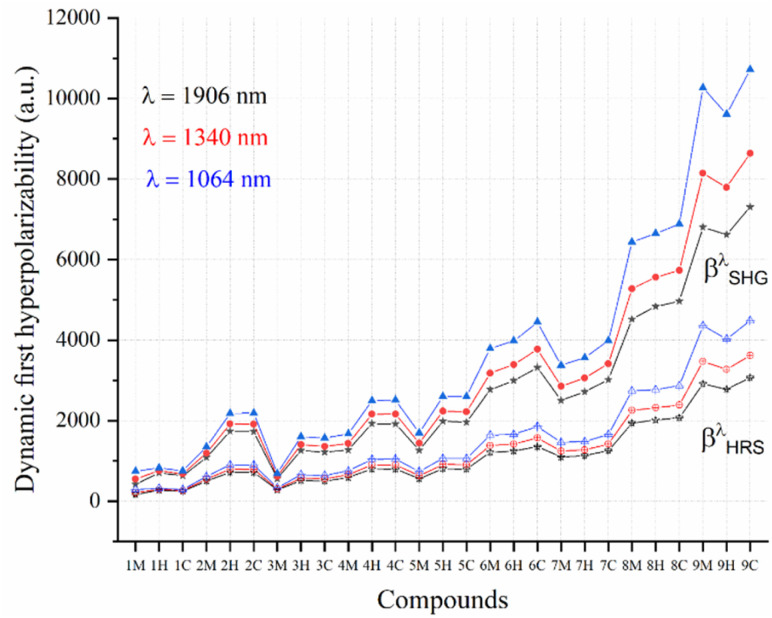
Dynamic first hyperpolarizabilities (*β*_SHG_^*λ*^ and *β*_HRS_^*λ*^) at 1064, 1340 and 1906 nm for the *i*M, *i*H, and *i*C (*i* = 1–9) series at the CAM-B3LYP/6-31G(d,p) level of theory.

The absorption spectra of the investigated compounds, calculated in THF at the TD-CAM-B3LYP/6-31G(d,p) level of theory, are presented in Fig. 9 and S1 (in the SI). These spectra confirm the absence of an electronic absorption at 532 nm, indicating that two-photon absorption (2PA) is negligible when the molecules are excited at 1064 nm. Additionally, all the compounds exhibit a broad optical transparency window beginning at around 350 nm, ensuring that no significant linear absorption interferes with the high-resolution hyper-Rayleigh scattering measurements in the visible and near-infrared regions. Our calculations show that the maximum absorption wavelength (*λ*_max_) corresponds to the S_0_ → S_1_ transition, predominantly of ICT character (Table 2). Based on these results, the frequency-dependent NLO properties, including dynamic polarizabilities and first hyperpolarizabilities, were computed at both non-resonant wavelengths (1064, 1340 and 1906 nm) and near-resonant conditions (556 nm, approximately twice the *λ*_Max_).

The calculated dynamic polarizability (*α*) and polarizability anisotropy (Δ*α*) values for the *i*M, *i*H, and *i*C compounds exhibit a consistent trend of *i*C > *i*H > *i*M across all wavelengths (1906–556 nm), reflecting the increasing electronic polarizability induced by the *o*-carborane substitution (Table S8). For all series, both (*α*) and Δ*α* show a modest increase with decreasing wavelength, which is attributed to mild dispersion effects under non-resonant conditions. The relatively small variation in values confirms that their optical response remains stable across the infrared-to-visible range and is primarily governed by their ground-state electronic structure rather than frequency-dependent effects.

On the other hand, frequency-dependent NLO calculations were carried out to examine three important first hyperpolarizability coefficients: the electro-optical Pockels effect (*β*_EOPE_*λ*) *β*(−*ω*;*ω*,0), second harmonic generation (*β*_SHG_^*λ*^) *β*(−2*ω*;*ω*,*ω*), and hyper-Rayleigh scattering (*β*_HRS_^*λ*^). Our findings demonstrate a strong correlation between *β*_HRS_^*λ*^ and *β*_*S*HG_^*λ*^; additionally, we observe a robust linear relationship between the static (*λ* = ∞) and dynamic first hyperpolarizability (*β*_SHG_^*λ*^ ↔ *β*_SHG_^∞^ and *β*_HRS_^*λ*^ ↔ *β*_HRS_^∞^, where *R*^2^ = 0.995) at *λ* = 1064, 1340, 1906 nm, as shown in Fig. S4 in the SI.

The dynamic first hyperpolarizability (*β*_SHG_^*λ*^ and *β*_HRS_^*λ*^) exhibits a pronounced increase as the incident wavelength is shortened, with a gradual increase observed from 1906 to 556 nm (Fig. 9). Furthermore, in the static regime, the first hyperpolarizability displays comparatively lower values than those recorded in the dynamic regime. Notably, a significant enhancement in the *β*_SHG_^*λ*^ and *β*_HRS_^*λ*^ values is observed at the incident wavelength of 556 nm, where the sharp increase is attributed to the resonant two-photon absorption process. This resonance occurs at the characteristic wavelength (*λ*_max_) leading to substantial amplification of the nonlinear optical response (Table 4).

**Table 4:** Calculated dynamic first hyperpolarizabilities (*β*_EOPE_^*λ*^, *β*_SHG_^*λ*^ and *β*_HRS_^*λ*^) at 1064 and 556 nm for the *i*M, *i*H, and *i*C (*i* = 1–9) non-centrosymmetric molecular series at the CAM-B3LYP/6-31G(d,p) level of theory

	*λ* (nm)	*β*_EOPE_^*λ*^	*β*_SHG_^*λ*^	*β*_HRS_^*λ*^
1M	1064	462	744	293
	556	959	26 937	11 144
1H	1064	773	827	322
	556	956	2803	1129
1C	1064	716	750	293
	556	892	2823	1138
2M	1064	1181	1347	616
	556	1575	12 940	5475
2H	1064	1895	2183	904
	556	2558	10 723	4458
2C	1064	1898	2185	904
	556	2587	11 717	4870
3M	1064	617	690	337
	556	811	5488	2348
3H	1064	1384	1602	655
	556	1898	8076	3331
3C	1064	1358	1568	641
	556	1886	8680	3581
4M	1064	1387	1670	756
	556	1991	28 245	11 808
4H	1064	2098	2495	1040
	556	2949	15 575	6463
4C	1064	2110	2512	1046
	556	3000	17 350	7197
5M	1064	1368	1698	742
	556	2012	32 418	13 514
5H	1064	2139	2604	1064
	556	3059	17 349	7174
5C	1064	2132	2598	1062
	556	3082	18 842	7792
6M	1064	3023	3796	1642
	556	4590	209 096	86 890
6H	1064	3251	3982	1660
	556	4739	36 712	15 252
6C	1064	3614	4446	1854
	556	5323	48 832	20 280
7M	1064	2739	3374	1461
	556	4074	116 244	48 356
7H	1064	2964	3565	1482
	556	4241	28 399	11 798
7C	1064	3310	3988	1658
	556	3046	37 084	15 403
8M	1064	4978	6436	2739
	556	7963	180 941	74 927
8H	1064	5296	6647	2771
	556	8066	252 257	104 624
8C	1064	5457	6884	2866
	556	8399	503 536	208 779
9M	1064	7460	10 271	4360
	556	12 894	114 154	47 146
9H	1064	7209	9613	4027
	556	11 818	201 648	83 520
9C	1064	7972	10 721	4486
	556	13 245	154 910	64 118

**Fig. 10:**
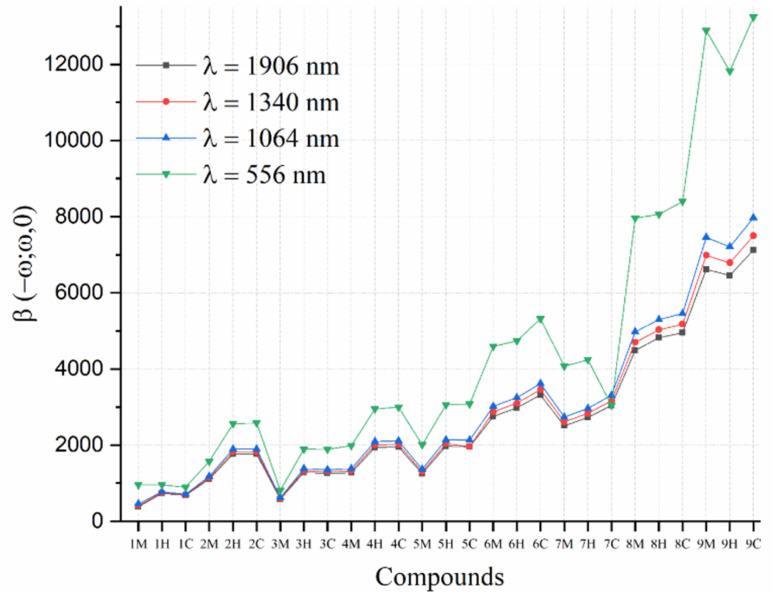
Dynamic first hyperpolarizabilities (*β*_EOPE_^*λ*^) at 556, 1064, 1340 and 1906 nm for the *i*M, *i*H, and *i*C (*i* = 1–9) series at the CAM-B3LYP/6-31G(d,p)/IEFPCM level of theory in THF.

On the other hand, DFT calculations indicate that the magnitude of *β*(−*ω*; *ω*, 0) slightly increases as the incident light wavelength decreases, following the trend of *β*_EOPE_^556^ > *β*_EOPE_^1064^ > *β*_EOPE_^1340^*β* > *β*_EOPE_^1907^ (Fig. 10). Additionally, the *β*(−*ω*; *ω*, 0) values show a sequential increase from the *i*M to *i*H to *i*C compounds, except for compound 9, where *β*(−*ω*; *ω*, 0) [9C] > *β*(−*ω*; *ω*, 0) [9M] > *β*(−*ω*; *ω*, 0) [9H]. This trend mirrors the order observed in the dynamic first hyperpolarizability. Overall, stronger NLO responses are seen at shorter wavelengths, with the *i*C compounds outperforming both the *i*H and *i*M compounds in NLO responses.

### Frequency dispersion factor

**Fig. 11:**
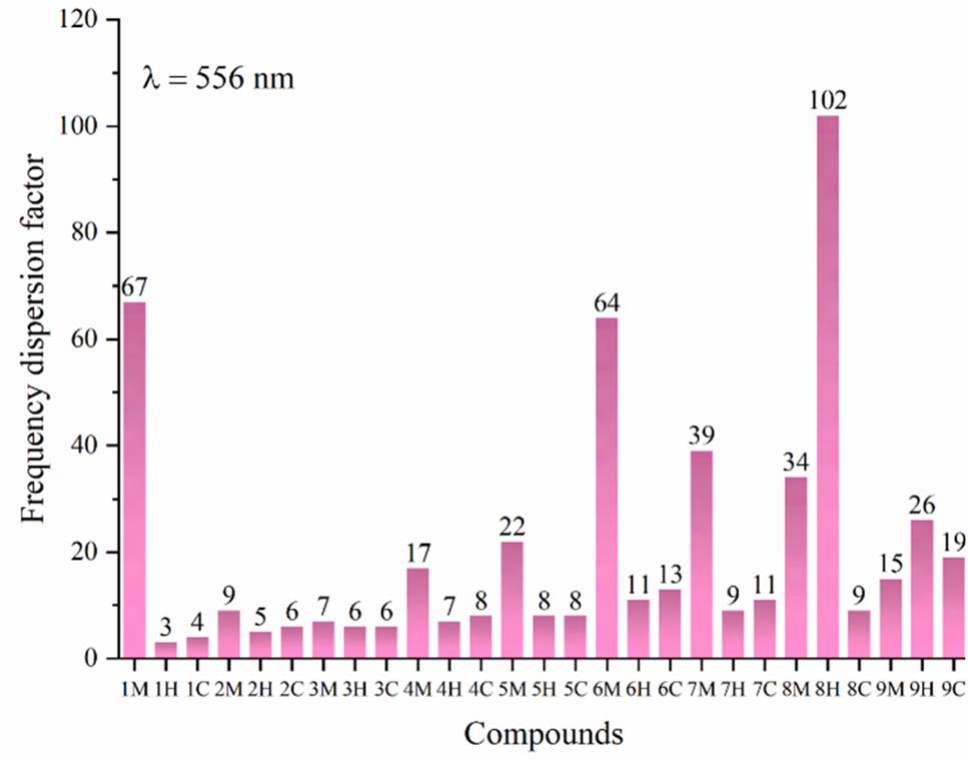
Variation in the frequency dispersion factor of *i*M, *i*H and *i*C (*i* = 1 to 9) calculated at the CAM-B3LYP/6-31G(d,p)/IEFPCM level of theory in THF.

The frequency dispersion factor for hyper-Rayleigh scattering (*β*_HRS_) in tetrahydrofuran was systematically calculated and presented in Fig. 11. At wavelengths of 1064, 1340, and 1906 nm, the frequency dispersion factor (FDF) is approximately unity. This value indicates that the intensity of the incident light has no significant impact on the NLO response of the title compounds. This phenomenon arises from the mismatch between the excitation frequencies and the intrinsic electronic transitions of the molecular system.

In contrast, as illustrated in Fig. 11, compounds 8H, 1M, 6M, 7M, and 8M display elevated FDF values at 556 nm (2.229 eV), corresponding to 102, 67, 64, 39, and 34, respectively. These values signify strong proximity to resonance conditions. The first resonance energy, defined as half of the first excitation energy, was determined for each compound, as follows: 8H (4.534 eV), 1M (4.614 eV), 6M (4.510 eV), 7M (4.537 eV), and 8M (4.355 eV). On the other hand, compounds 1H, 1C, 2H, 2C, 3H, and 3C exhibit minimal FDF^556^ values, ranging between 3 and 6, which indicates their positioning within the off-resonance regime, where dispersion effects are negligible. Additionally, compounds *i*C and *i*H (where *i* = 1 to 7) display moderate FDF values, generally below 12. These results reflect the modest influence of incident wavelength on the NLO response, suggesting that these compounds are approaching resonance conditions at 556 nm. Overall, it can be observed that the *i*H and *i*C compounds exhibit FDF^556^ values that are similar in magnitude and significantly smaller compared to those of the *i*M molecules.

### Two-level model analysis of first hyperpolarizability

The sum-over-states (SOS) method represents one of the prevailing approaches for theoretically estimating first hyperpolarizability.^75–80^ The comprehensive expression for the SOS formula pertaining to the component denoted as ABC of first hyperpolarizability is precisely defined as follows:21
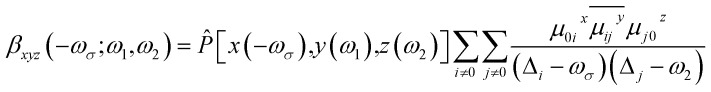
where 
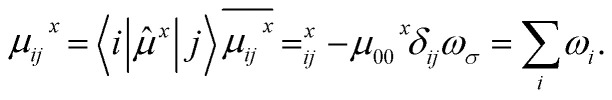


The symbol *ω* represents the energy associated with external fields. *Δ*_*i*_ signifies the excitation energy of state *i* in relation to the ground state (0). *P̂* represents the permutation operator, which is responsible for the manipulation of the *xyz* indices within the *β*-components. Furthermore, *µ*_*ij*_^*x*^ denotes the *x*-component of the transition dipole moment, characterizing the transition between states *i* and *j*.

The molecular NLO responses of the compounds exhibit a profound connection to their electronic absorption characteristics. This intricate relationship was elucidated through the application of the well-established two-level model developed by Oudar and Chemla. Within this theoretical framework, one can articulate the static hyperpolarizabilities with precision and relevance.^81,82^22
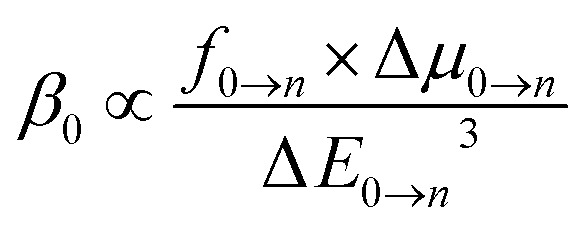
In the context of the specified variables, where Δ*E* represents the excitation energy (S_0_ → S_*n*_), *f* stands for the oscillator strength, and Δ*µ* signifies the difference in dipole moment between the ground state (S_0_) and the excited state (S_*n*_), a critical relationship emerges. According to this relationship, a clear and noteworthy trend is observed that the static first hyperpolarizability (*β*_0_) displays inverse proportionality to Δ*E*^3^. This observation underscores the pivotal role played by lower excitation energies (*ΔE*_0→_*_n_*_*n*_), larger dipole moments (Δ*µ*_0→_*_n_*), and higher oscillator strengths (*f*_0→_*_n_*) in generating the highest values of *β*_0_ for materials.

It is well established that the molecular NLO response, as evaluated *via* the SOS method, is significantly influenced by the number of electronically excited states incorporated into the calculation. In this context, we conducted a systematic investigation into the dependence of the first hyperpolarizability (*β*_SOS_) on the number of excited states considered, with particular emphasis on the inclusion of up to 120 excited states, as illustrated in Fig. S5. The resulting data clearly demonstrate that the *β*_SOS_ values exhibit pronounced convergence behavior upon the incorporation of 120 excited states, thereby validating the adequacy of this level of state inclusion for accurate and reliable first hyperpolarizability estimations.

**Fig. 12:**
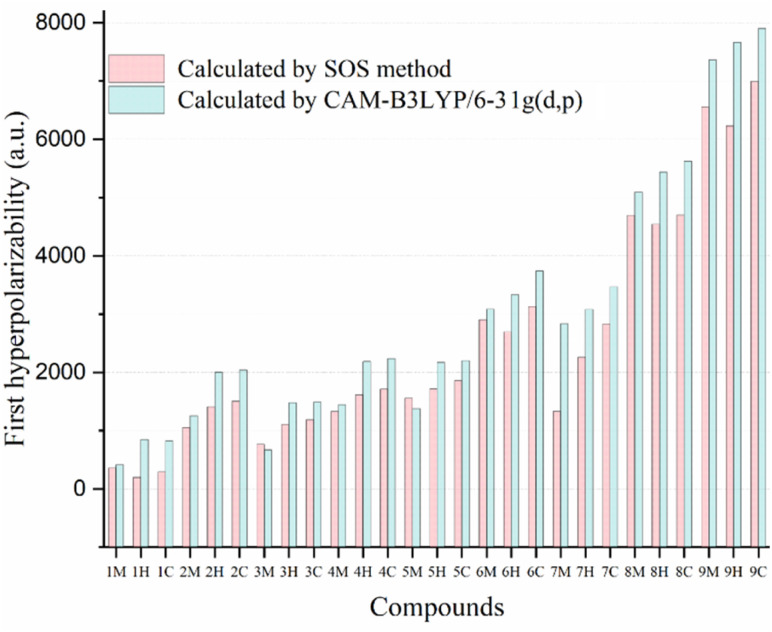
Computed static first hyperpolarizabilities of the title compounds using various computational methods.

As illustrated in Fig. 12, the *β*_SOS_ values exhibit qualitative agreement with the overall trends observed for the *β*_0_ values obtained at the CAM-B3LYP level of theory. However, notable discrepancies are observed, which are primarily attributed to the inherent approximations and limitations of the SOS methodology. Specifically, the SOS approach consistently underestimates the first hyperpolarizability magnitudes relative to those calculated *via* the CAM-B3LYP functional.^56,83^ Additionally, a two-level model analysis reveals that the first excited state exerts (S_0_ → S_1_) a predominant influence on the first hyperpolarizability values of the studied compounds *i*M, *i*H, and *i*C (*i* = 1 to 9). This predominant excited state is distinguished by a pronounced dipole moment variation relative to the ground state (Δ*µ*) and a markedly elevated oscillator strength (*f*), parameters that significantly enhance its contribution to the overall NLO response.

**Fig. 13:**
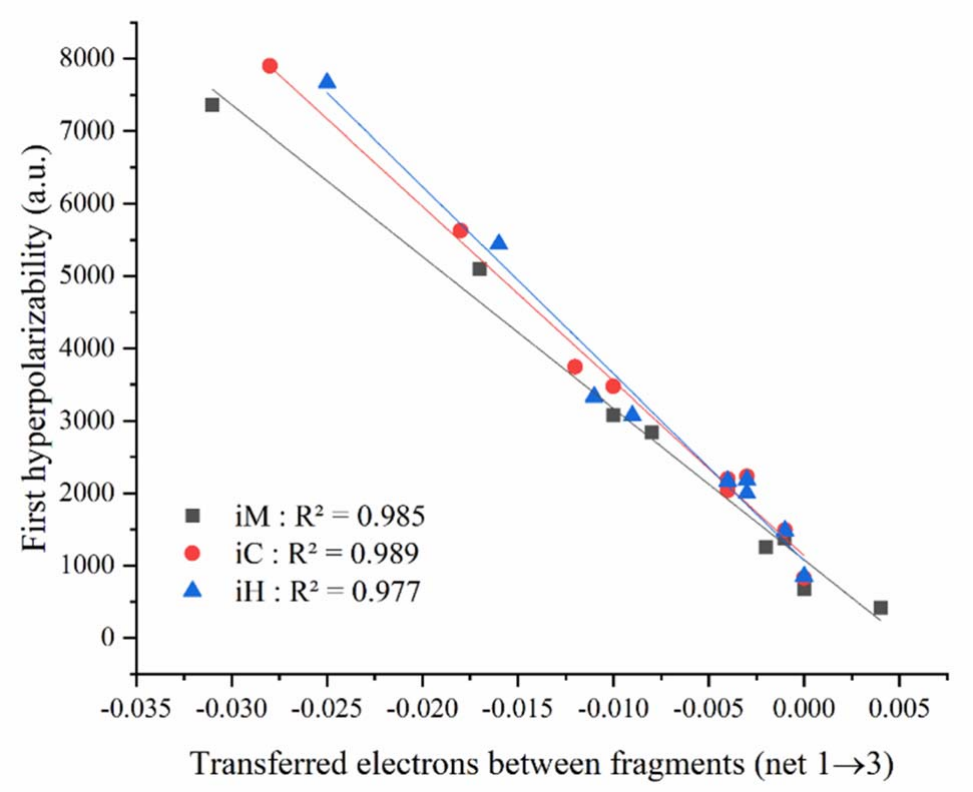
Correlation between first hyperpolarizability and net electron transfer between fragments (1 → 3) for the *i*M, *i*H and *i*C compounds (*i* = 1 to 9) of the first excited state (S_0_ → S_1_).

The analysis of the parameters associated with the first excited state reveals insightful trends related to the electronic behavior of the studied molecules. Notably, a strong inverse linear relationship is observed between the first hyperpolarizability and the net number of electrons transferred from fragment 1 to fragment 3 (net 1 → 3) across the *i*M, *i*H, and *i*C series (Fig. 13). The high regression coefficients (*R*^2^ = 0.985, 0.977 and 0.989 for *i*M, *i*H and *i*C, respectively) confirm the robustness of this correlation within each molecular class. This trend suggests that as the extent of electron transfer toward Frag-3 increases (more negative net 1 → 3), the first hyperpolarizability value significantly increases, implying enhanced ICT. This enhancement in ICT contributes to a greater asymmetry in electron distribution, a key factor in boosting the second-order NLO response. Notably, the *i*C compounds display the highest net electron transfer values (net 1 → 3), followed by *i*H and *i*M. This consistent trend across all the series highlights the key role of fragment-based charge redistribution in tuning NLO properties. Thus, the direction and magnitude of electron flow emerge as effective predictors for designing efficient D–A-type *o*-carborane-based NLO chromophores.

**Fig. 14:**
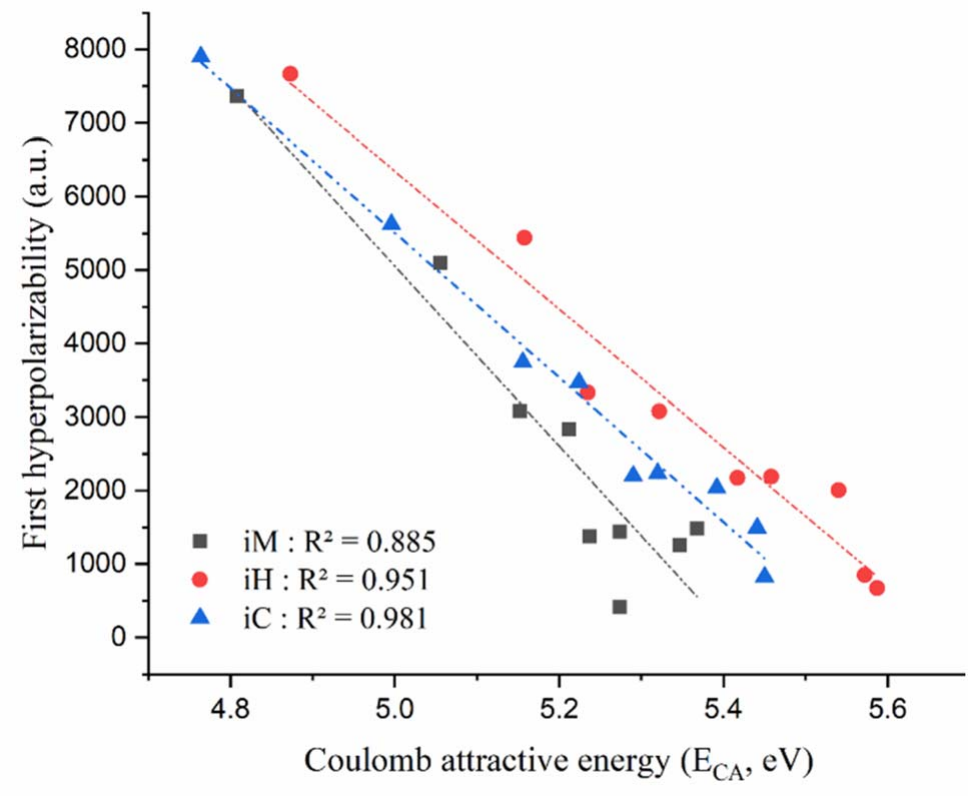
Correlation between the Coulomb attractive energy (*E*_CA_) of first excited state and the first hyperpolarizability for the *i*M, *i*H, and *i*C non-centrosymmetric molecular series.

On the other hand, in all the investigated compounds, the Coulomb attractive energy (*E*_CA_) associated with the first excited state consistently exceeds the corresponding vertical excitation energy (see Tables 2 and S4), emphasizing the critical role of electron–hole Coulomb interactions in stabilizing the excited-state manifold.^84,85^ The magnitude of *E*_CA_ provides a direct measure of electron–hole binding strength and the degree of charge localization within the excited state. As depicted in Fig. 14, an unambiguous inverse correlation is observed between *E*_CA_ and the computed first hyperpolarizability, indicating that enhanced coulombic attraction tends to suppress charge delocalization and intramolecular polarization, thereby diminishing the NLO response. These observations underscore the fundamental interplay between electronic structure and NLO properties and highlight the importance of modulating excitonic interactions to optimize molecular first hyperpolarizability in the design of advanced optoelectronic materials. Furthermore, a clear linear relationship was observed between the first hyperpolarizability and the dipole moment variation relative to the ground state upon first excitation (Fig. S6). These results are in good agreement with previous reports, which also identified Δ*µ* as a key electronic descriptor for predicting first hyperpolarizability in compounds.^56,86^

Our results confirm the expected trend that the first hyperpolarizability of the *i*M, *i*H, and *i*C compounds reaches its highest values when Δ*µ*_0–1_ and *f*_0–1_ are simultaneously maximized, while the transition energy (Δ*E*_0–1_) and the Coulomb attractive energy (*E*_CA_) of the first excited state are minimized. This combined electronic profile promotes efficient intramolecular charge transfer and enhances the overall nonlinear optical response of the systems under study.

To elucidate the electronic factors influencing NLO activity, compounds 1C and 9C were selected as representative case studies. Compound 9C exhibits a significantly higher static first hyperpolarizability (*β*_0_ = 7902 a.u. [*β*_HRS_ = 3326 a.u.]) compared to compound 1C ((*β*_0_ = 828 a.u.) [(*β*_HRS_ = 322 a.u.)]), corresponding to a nearly nine-fold enhancement in *β*_0_. This pronounced increase in NLO response for 9C is attributed to its larger dipole moment (Δ*µ*_0–1_ = 5.003 a.u.), lower vertical excitation energy (Δ*E*_0–1_ = 4.187 eV), and reduced Coulomb attractive energy (*E*_CA_ = 4.764 eV). In contrast, compound 1C is characterized by a markedly smaller dipole moment change (Δ*µ*_0–1_ = 0.453 a.u.) and higher values of Δ*E*_0–1_ (5.02 eV) and *E*_CA_ (5.450 eV). The results indicate that excellent nonlinear optical materials are characterized by lower excitation energies, significant dipole moment variations in the first excited state, and reduced Coulomb attractive energy values (Fig. 14). These observations highlight *E*_CA_ as a novel and promising parameter for predicting and optimizing the NLO properties of materials.

### Photoluminescence and second-order NLO response

**Fig. 15:**
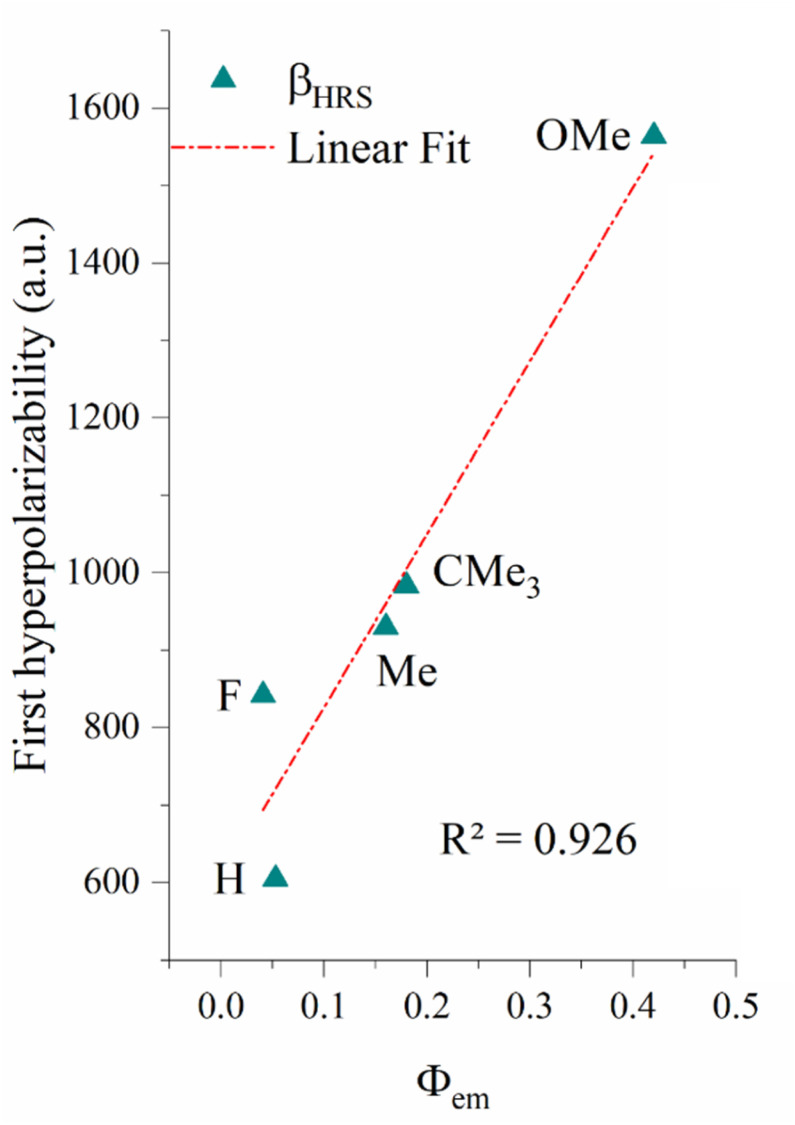
Linear correlation between the photoluminescence quantum yield *Φ*_em_ (*Φ*_em_ value from ref. 49) and static first hyperpolarizability (*β*_HRS_) for the *i*C non-centrosymmetric compounds (*i* = 2 to 6).

To predict experimentally accessible parameters that correlate with first hyperpolarizability, we investigated the relationship between the photoluminescence quantum yield (*Φ*_em_)^49^ and the calculated first hyperpolarizability for *i*C non-centrosymmetric compounds (*i* = 2 to 6). As shown in Fig. 15, a strong linear correlation (*R*^2^ = 0.926) is observed, revealing that *Φ*_em_ can serve as a predictive indicator for first hyperpolarizability in D–A-type *o*-carboranyl luminophores. The resulting regression equation, *β*_HRS_ = 603 + 2238*Φ*_em_, quantitatively describes this correlation and can be employed to estimate second-order NLO responses based on experimental emission efficiencies.

The unsubstituted compound 3H (R = –H) exhibits the lowest photoluminescence quantum yield (*Φ*_em_)^49^ and first hyperpolarizability (*β*) values, indicating its inefficient intramolecular charge transfer (ICT) behavior and minimal charge redistribution upon excitation. As shown in Fig. 5 and Table S6, the net transferred electron density between molecular fragments in this derivative is nearly negligible, consistent with its weak NLO response.

Substitution with a fluorine atom (R = –F) induces a slight increase in both (*Φ*_em_) and *β*_HRS_. This limited enhancement can be attributed to the strong inductive electron-withdrawing effect of fluorine, which mildly perturbs the electronic distribution and stabilizes the excited state (see Fig. 5 and Table 2). However, the absence of a mesomeric effect and the inherently low polarizability of –F restrict its ability to promote effective ICT.

On the other hand, the introduction of electron-donating alkyl groups (–Me and –CMe_3_) leads to moderate improvements in both (*Φ*_em_) and *β*_HRS_ (Fig. 15). These substituents increase the electronic density within the π-system through hyperconjugative and inductive effects, facilitating partial ICT. The corresponding increase in ICT, particularly between fragments 1 → 3 and 2 → 3 (as evidenced in Fig. 6 and Table S6-7), supports a greater degree of polarization in the excited state.

Notably, the methoxy-substituted derivative (R = –OMe) displays the highest (*Φ*_em_) and *β*_HRS_ (0.45 and 1564 a.u., respectively) among the studied compounds (Fig. 15). This pronounced enhancement is attributed to the strong +M mesomeric effect of the –OMe group, which significantly increases the extent of ICT. Quantitative analysis reveals the highest net electron transfer between the donor and acceptor fragments for this compound, substantially exceeding those observed for –H, –F, –Me, and –CM_3_. The resulting increase in Δ*µ* (variation in dipole moment with respect to the ground state) reinforces both the photoluminescence quantum yield (*Φ*_em_) and first hyperpolarizability.

Collectively, these findings confirm that increasing the electron-donating strength of the R substituent enhances the ICT efficiency, which in turn amplifies both the photoluminescence and 2nd-order NLO responses of non-centrosymmetric compounds. The strong linear correlation (Fig. 15) established between *Φ*_em_ and *β*_HRS_ underscores the feasibility of using photoluminescence parameters as predictive descriptors of the NLO performance of π-conjugated non-centrosymmetric molecules.

## Conclusions

A comprehensive computational investigation was carried out in three non-centrosymmetric molecular series, *i*M, *i*H, and *i*C, at the CAM-B3LYP/6-31G(d,p) level of theory using the IEFPCM solvation model in THF. These series differ in the electron-accepting core, with trimethylsilyl-acetylene in *i*M, an *o*-carborane cage in *i*H, and a trimethylsilyl-functionalized *o*-carborane cage in *i*C, and incorporate various donor substituents (R = –CF_3_, –F, –H, –Me, –*t*Bu, –OMe, –OH, –NH_2_, and –NMe_2_). Their ground- and excited-state geometries, absorption and emission characteristics, intrafragment charge transfer, and first- and second-order NLO responses were systematically analyzed. The *i*C series consistently showed an enhanced dipole moment variation, oscillator strength (*f*), Coulomb attractive energy (*E*_CA_), net electron transfer between the substituent and the *o*-carborane cage, Stokes shift, and NLO activity compared to the *i*H analogues, while the *i*M series exhibited the lowest values. Across all series, these properties increased with the electron-donor strength of the substituent, with the exception of 6H (–OMe) and 7H (–OH) in the *i*H series, which showed a marked decrease in Stokes shift due to their ground-state conjugation induced by the oxygen lone pair and reduced dihedral angle, *φ*_2_. Strong correlations were found between the first hyperpolarizability and both the net electron transfer between fragments (1 → 3) and *E*_CA_, as well as between the photoluminescence quantum yield (*Φ*_em_) and *β* for the *i*C derivatives (*i* = 2–6). The *o*-carborane derivatives bearing –NMe_2_ substituents exhibit the largest first hyperpolarizabilities in this study and thus appear suitable for future development as second-order NLO materials. These results validate that enhancing the electron-donating ability of the R substituent improves the efficiency of intramolecular charge transfer, thus boosting both the photoluminescence and second-order nonlinear optical responses in *o*-carborane-based non-centrosymmetric molecules.

## Author contributions

Conceptualization and methodology: D. H. and H. C.; investigation: D. S., M. Z., D. H., O. A. and G. H.; writing – original draft preparation: D. H. and H. C.; writing – review and editing: D. S., M. Z., D. H., G. H. and H. C.; and data curation: D. S., M. Z., D. H., A. O., G. H. and H. C. All authors have read and agreed to the published version of the manuscript.

## Conflicts of interest

There are no conflicts to declare.

## Supplementary Material

RA-016-D6RA00681G-s001

## Data Availability

Supplementary information (SI): calculated static polarizability and its anisotropy polarizability and first hyperpolarizability of 1C at CAM-B3LYP/basis sets/IEFPCM level in THF; TD-CAM-B3LYP/6-31G(d,p)) absorption (abs), emission (em) wavelengths and transition dipole moment (*µ*_0→1_) for compounds *i*M, *i*H and *i*C; experimental values are given in parentheses from the work of Dong Kyun You *et al.*; total energy (*E*, a.u.), HOMO and LUMO energies (eV) of the ground state (S_0_), first excited state (S_1_) wavelength (nm), and oscillator strength of the emission transition S_1_ → S_0_ for compounds *i*M, *i*H, and *i*C, calculated at the CAM-B3LYP/6-31G(d,p)/IEFPCM level in THF; TD-DFT calculated excited-state (S_*n*_) parameters of the title compounds: transition energies (eV), wavelengths (nm), oscillator strengths, and electronic configurations, calculated at the CAM-B3LYP/6-31G(d,p)/IEFPCM level in THF; calculated overlap (*S*_r_), *D* index (Å), *H* index (Å), variation of dipole moment with respect to ground state (Δ*µ*, a.u.), hole delocalization index (HDI), electron delocalization index (EDI), ghost-hunter index 1st and 2nd, Coulomb attractive energy (ECA) for *i*M, *i*H and *i*C compounds in the first excited state at the CAM-B3LYP/6-31G(d,p)/IEFPCM level; contribution of each fragment to hole and electron (I), variation of population number of fragment (II) and intrafragment electron redistribution of fragment (III) calculated in tetrahydrofuranat the CAM-B3LYP/6-31G(d,p)/IEFPCM level; intrinsic charge transfer percentage, CT (%), intrinsic local excitation percentage, LE (%) and transferred electrons between fragments, calculated for the electronic transition S_0→*i*_ of the *i*M, *i*H and *i*C compounds at the CAM-B3LYP/6-31G(d,p)/IEFPCM level in tetrahydrofuran; calculated dynamic polarizability 〈*α*〉 and polarizability anisotropy (Δ*α*); simulated absorption spectrum of the title compounds; CDD for different excited states of the title compounds. CDD was calculated as a difference between the corresponding excited state and the ground state of the considered system using the CAM-B3LYP/6-31G(d,p)/IEFPCM level of theory; correlation between static first hyperpolarizability β_HRS and (0;0,0) for *i*M, *i*H and *i*C compounds (*i* = 1 to 9), calculated at the CAM-B3LYP/6-31G(d,p)/IEFPCM level in THF; correlation between dynamic and static first hyperpolarizability β_HRS for *i*M, *i*H and *i*C compounds (*i* = 1 to 9), calculated at the CAM-B3LYP/6-31G(d,p)/IEFPCM level in THF; plots of static first hyperpolarizability values as computed in the SOS formalism as a function of the number of excited states for for *i*M, *i*H and *i*C compounds (*i* = 1 to 9), calculated at the CAM-B3LYP/6-31G(d,p)/IEFPCM level in THF; linear correlation between first hyperpolarizability and dipole Moment Variation (Δ*µ*) in the first excited state for *i*M, *i*H and *i*C (*i* = 1 to 9) compounds. See DOI: https://doi.org/10.1039/d6ra00681g.
