# Supplementary material for: Structural, linear, and nonlinear optical properties of ortho-carboranyl luminophores: insights from DFT and TD-DFT studies
Source: RSC Adv. 2026 Apr 22;16(23):21220–39. doi: 10.1039/d6ra00681g (PMC13100881; doi:10.1039/d6ra00681g)
Supplement: RA-016-D6RA00681G-s001 [file RA-016-D6RA00681G-s001.pdf]

Supplementary Information (SI) for RSC Advances.
This journal is © The Royal Society of Chemistry 2026

Structural, Linear, and Nonlinear Optical Properties of ortho-Carboranyl Luminophores: Insights from DFT and TD-DFT Studies

Djamila Samsar,[1,2] Douniazed Hannachi,[3,4] Meriem Zaidi, [4, 5] Guillaume Hoffmann,[6] Olivier Aroule, [6] Henry Chermette [6]

[1] Institut D'Hygiène et Sécurité Industrielle, Département de Socle commun Hygiène et Sécurité Industrielle, Université de Batna-2, Algérie

[2] Department of Chemistry, Faculty of Matter Sciences, Laboratory of Materials Chemistry and the Living: Activity & Reactivity (LCMVAR), University of Batna-1, Algeria

[3] Laboratoire d'Électrochimie, d'Ingénierie Moléculaire et de Catalyse Redox (LEIMCR), Faculté de Technologie, Université Ferhat Abbas, Sétif-1, Algérie.

[4] Département de Chimie, Faculté des Sciences, Université de Setif-1, Algérie

[5] Laboratoire de Chimie, Ingénierie Moléculaire et Nanostructures (LCIMN), Université Ferhat Abbas Sétif 1, Sétif 19000, Algérie.

[6] Université de Lyon, Université Claude Bernard Lyon 1, Institut des Sciences Analytiques, UMR CNRS 5280, 69622 Villeurbanne Cedex, France

List of table and figures

Table S1: Calculated static polarizability and its anisotropy polarizability and first hyperpolarizability of 1C at CAM-B3LYP/basis sets/IEFPCM level in THF	S3
Table S2: TD-CAM-B3LYP/6-31G(d,p)) absorption (λ_{abs}), emission (λem) wavelengths and transition dipole moment ($\mu_{0\rightarrow1}$) for compounds iM, iH and iC; experimental values are given in parentheses from the work of Dong Kyun You et al.[1]	S3
Table S3: Total energy (E, a.u.), HOMO and LUMO energies (eV) of the ground state (S0), first excited state (S1) wavelength (nm), and oscillator strength of the emission transition S1→S0 for compounds iM, iH, and iC, calculated at the CAM-B3LYP/6-31G(d,p)/IEFPCM level in THF	S5
Table S4: TD-DFT calculated excited-state (Sn) parameters of the title compounds: transition energies (eV), wavelengths (nm), oscillator strengths, and electronic configurations, calculated at the CAM-B3LYP/6-31G(d,p)/IEFPCM level in THF.	S6
Table S5. Calculated Overlap (Sr), D Index (Å), H index(Å), Variation of dipole moment	S8

with respect to ground state (Δμ, a.u.), Hole delocalization index (HDI), Electron delocalization index (EDI), Ghost-hunter index 1st and 2en, Coulomb attractive energy (E_{CA}) for iM, iH and iC compounds in the first excited state at the CAM-B3LYP/6-31G(d,p)/IEFPCM Level	
Table S6. Contribution of each fragment to hole and electron (I), Variation of population number of fragment (II) and intrafragment electron redistribution of fragment (III) calculated in tetrahydrofuranat the CAM-B3LYP/6-31G(d,p)/IEFPCM Level	S10
Table S7. Intrinsic charge transfer percentage, CT(%), Intrinsic local excitation percentage, LE(%) and Transferred electrons between fragments, calculated for the electronic transition $S_{0\rightarrow i}$of the iM, iH and iC compounds at the CAM-B3LYP/6-31G(d,p)/IEFPCM Level in tetrahydrofuran	S14
Table S8. Calculated dynamic polarizability (⟨α⟩) and polarizability anisotropy (Δα)	S18
Figure S1. Simulated absorption spectrum of the title compounds	S20
Figure S2. CDD for different excited states of the title compounds. CDD was calculated as a difference between the corresponding excited state and the ground state of the considered system using the CAM-B3LYP/6-31G(d,p)/IEFPCM level of theory. Blue regions indicate negative electron density, whereas orange regions correspond to positive electron density. The isosurface level is set to be 0.001807.	S26
Figure S3 : Correlation Between static First Hyperpolarizability β_{HRS} and β(0;0,0) for iM, iH and iC compounds (i=1 to 9), calculated at the CAM-B3LYP/6-31G(d,p)/IEFPCM level in THF.	S27
Figure S4. Correlation Between dynamic and static First Hyperpolarizability β_{HRS} for iM, iH and iC compounds (i=1 to 9), calculated at the CAM-B3LYP/6-31G(d,p)/IEFPCM level in THF	S28
Figure S5. Plots of static first hyperpolarizability values as computed in the SOS formalism as a function of the number of excited states for foriM, iH and iC compounds (i=1 to 9), calculated at the CAM-B3LYP/6-31G(d,p)/IEFPCM level in THF.	S29
Figure S6. Linear Correlation Between First Hyperpolarizability and Dipole Moment Variation (Δμ) in the First Excited State for iM, iH and iC (i=1 to 9) compounds	S30

Table S1. Calculated static polarizability and its anisotropy polarizability and first hyperpolarizability of 1C at CAM-B3LYP/basis sets/IEFPCM level in THF

		6-31++G(d,p)	6-31++G	6-31+G(d,p)	6-31+G	6-31G(d,p)	6-311G(d)	6-31G(d)
Number of basis functions		774	501	747	474	619	665	538
α	1C	438	423	437	422	394	418	391
$\Delta\alpha$	1C	190	180	190	181	253	200	203
β_{HRS}^{∞}/ DR	1C	220/6.112	178/6.517	213/6.155	174/6.599	322/6.341	422/7.913	324/6.350

Table S2: TD-CAM-B3LYP/6-31G(d,p)) absorption (λ_{abs}), emission (λem) wavelengths and transition dipole moment ($\mu_{0\rightarrow1}$) for compounds iM, iH and iC; experimental values are given in parentheses from the work of Dong Kyun You et al.[1]

Mi	λ_{abs} (nm)	λ_{em} (nm)	$\mu_{0\rightarrow1}$ *(a.u.)*
1M	270	350.27	3.372
1H	245	326.22	2.973
1C	247 (265)	399.36 (454)	2.967
2M	266	346.59	3.279
2H	245	422.24	2.829
2C	247 (266)	420.04 (475)	2.828
3M	266	346.65	3.273
3H	245	417.74	2.830
3C	247 (267)	415.59 (476)	2.823
4M	269	351.48	3.435
4H	250	430.79	3.009
4C	252 (274)	428.84 (491)	3.006
5M	270	351.49	3.538
5H	251	430.46	3.129
5C	253 (275)	428.23 (492)	3.120
6M	275	358.23	3.508
6H	259	336.25	3.064
6C	262 (289)	455.32 (516)	3.060
7M	273	355.51	3.409
7H	256	332.95	2.954
7C	260	451.03	2.955
8M	285	373	3.543
8H	273	506.18	3.118

8C	276	505.16	3.124
9M	303	381.88	3.956
9H	292	520.38	3.550
9C	296	522.67	3.351

Table S3: Total energy (E, a.u.), HOMO and LUMO energies (eV) of the ground state (S0), first excited state (S1) and oscillator strength of the emission transition S1→S0 for compounds iM, iH, and iC, calculated at the TD-CAM-B3LYP/6-31G(d,p)/IEFPCM level in THF

		E	HOMO	LUMO	f
1M	GS	-1284.728	-7.483	-0.364	/
	S1	-1284.581	-6.993	-1.053	1.557
1H	GS	1130.667	-8.046	-0.383	/
	S1	-1130.509	-7.456	-1.143	1,352
1C	GS	1539.264	-8.033	-0.410	/
	S1	-1539.115	-7.619	2.304	1,15
2M	GS	-1046.980	-7.293	-0.119	/
	S1	1046.980	-6.754	-0.762	1,507
**2H**	GS	-892.920	-7.712	-0.118	/
	S1	-892.772	-7.320	-2.286	1,225
**2C**	GS	-1301.517	-7.701	-0.160	/
	S1	-1301.373	-7.293	-2.231	1,111
3M	GS	-947.766	-7.312	-0.116	
	S1	-947.618	-6.785	-0.777	1,504
**3H**	GS	-793.705	-7.771	-0.118	/
	S1	-793.557	-7.412	-2.284	1,217
**3C**	GS	-1202.302	-7.760	-0.157	/
	S1	-1202.157	-7.381	-2.254	1,103
**4M**	GS	-987.061	-7.196	-0.083	/
	S1	-986.615	-6.661	-0.720	1,608
**4H**	GS	-833.001	-7.570	-0.081	/
	S1	-832.856	-7.226	-2.253	1,299
**4C**	GS	-1241.598	-7.561	-0.123	/
	S1	-1241.457	-7.195	-2.230	1.1787
**5M**	GS	-1104.933	-7.197	-0.090	/
	S1	-1104.787	-6.667	-0.726	1,674
**5H**	GS	-950.873	-7.572	-0.088	/
	S1	-950.728	-7.211	-2.259	1,339
**5C**	GS	-1359.470	-7.568	-0.125	/
	S1	-1359.328	-7.200	-2.231	1,216
6M	GS	-1062.247	-6.997	-0.027	/

	S1	-1062.104	-6.449	-0.601	1,651
6H	GS	-908.187	-7.199	-0.002	/
	S1	-908.036	-6.696	-0.555	1,431
6C	GS	-1316.784	-7.210	-0.066	/
	S1	1316.649	-6.859	-2.194	1,223
7M	GS	-1022.969	-6.997	-0.022	/
	S1	-1022.825	-6.478	-0.604	1,589
7H	GS	-868.909	-7.248	-0.002	/
	S1	-868.756	-6.737	-0.557	1,367
7C	GS	-1277.506	-7.258	-0.061	/
	S1	1277.370	-6.868	-2.201	1,178
8M	GS	-1003.105	-6.627	0.037	/
	S1	-1002.968	-6.061	-0.411	1,694
8H	GS	-849.045	-6.788	0.046	/
	S1	-848.917	-6.406	-2.151	1,356
8C	GS	-1257.642	-6.787	0.000	/
	S1	-1257.517	-6.327	-2.138	1,235
9M	GS	-1081.669	-7.255	-0.123	/
	S1	-1081.536	-5.963	-0.419	1,798
9H	GS	-927.609	10.569	0.059	/
	S1	-927.484	-6.271	-2.146	1,4319
9C	GS	1336.206	-6.434	0.018	/
	S1	-1336.086	-6.240	-2.154	1,296

Table S4. TD-DFT calculated excited-state (Sn) parameters of the title compounds: transition energies (ΔE(eV)), absorption wavelengths (λ_{abs} (nm)), oscillator strengths (*f*) and electronic transition, calculated at the CAM-B3LYP/6-31G(d,p)/IEFPCM level in THF.

Compounds	Sn	λ (nm)	E (eV)	*f*	Electronic Transition
1M	S1	270	4.614	1.285	H→L 92 %
	S11	183	6.763	1.327	H-1→L+2 46 %, H-2→L+1 30%
1H	S1	245	5.053	1.094	H →L 95%
	S7	185	6.7	1.293	H-1→L+1 54%, H-2→L+2 32%
1C	S1	247	5.020	1.087	H→L 95%
	S7	185	6.681	1.086	H-1→L+1 50%, H-2→ L+2 27%
2M	S1	266	4.651	1.225	H →L 92%
	S11	183	6.766	1.326	H-1→L+2 44%, H-3→L+1 28%
2H	S1	245	5.043	0.990	H →L 92%
	S7	186	6.663	0.740	H-1→L+1 23%, H-1→L+2 18%
	S8	182	6.808	0.598	H-3→L 30%, H →L+3 14%
2C	S1	247	5.000	0.985	H→L 92%
	S7	186	6.645	0.682	H-1→L+1 36%, H-2→L+2 28%
	S8	183	6.779	0.676	H-3 -> L 33%, H-1→ L+1 24%
3M	S1	266	4.656	1.222	H → L 92%
	S11	183	6.759	1.288	H-3→ L+1 54%, H-1 →L+2 32%
	S14	170	7.282	0.147	H-4→ L+4 15%, H-3 → L+1 14%

	S23	159	7.803	0.042	H-6→ L 48%, H-11→L 12%
3H	S1	245	5.064	0.994	H → L 95%
	S7	186	6.670	0.937	H-2 →L+1 42%, H-1→ L+3 33%
3C	S1	247	5.024	0.987	H → L 95%
	S7	186	6.651	0.865	H-2→ L+1 40%, H-1 → L+3 32%
	S8	183	6.783	0.496	H-3→ L 36%, H-2 -> L+121%
4M	S1	269	4.601	1.331	H → L 92%
	S11	183	6.748	1.354	H-3 → L+1 50%, H-1 → L+2 32%
4H	S1	250	4.950	1.099	H → L 92%
	S7	187	6.615	0.437	H-1→ L+3 27%, H-2 → L +1 23%
	S8	183	6.757	0.747	H → L+2 27%, H-2 → L+1 24%
	S9	183	6.778	0.29	H → L+2 46%, H-3 → L+2 12%
4C	S1	252	4.909	1.039	H → L 92%
	S7	188	6.593	0.36	H-1→ L+3 24%, H-2 → L+1 20%
	S8	184	6.734	1.052	H-2→ L+1 38% , H-3→ L+2 23%
5M	S1	270	4.592	1.409	H→ L 92%
	S11	184	6.736	1.431	H-3→L+1 50%, H-1→ L+2 20%
	S24	158	7.819	0.321	H -6→ L 25%,H-2→ L+524%
5H	S1	251	4.938	1.185	H-1→ L 92%
	S7	187	6.611	0.541	H-2→ L+3 28%, H-2→ L+1 24%
	S8	184	6.753	0.832	H-2→L+1 24%, H→ L+2 20%
	S9	183	6.779	0.188	H→L+2 52%, H-3→L+214%
5C	S1	253	4.907	1.175	H→L 92%
	S7	188	6.590	0.431	H-1→L+3 27%, H-2→ L+1 20%
	S8	184	6.730	1.093	H-1→L+1 36%, H-3→L 24%
6M	S1	275	4.510	1.361	H→L 92%
	S9	195	6.366	0.161	H-2 → L 32%. H →L+3 32%
	S11	183	6.765	1.431	H-3→ L+126%. H-2→L+126%
	S21	160	7.729	0.404	H-2 → L+3 28%. H -1→L+320%
6H	S1	259	4.792	1.103	H → L 92%
	S8	184	6.732	1.367	H-2 → L+1 51%, H-3 →L 16%
6C	S1	262	4.731	1.093	H → L 92%
	S9	184	6.724	1.467	H-2 → L+1 56%, H-3 →L 13%
7M	S1	273	4.537	1.293	H→L 92%
	S11	183	6.776	1.434	H-1→L+1 32%,H-3→L+2 14%
	S22	159	7.781	0.607	H-1→L+3 46%, H-4→ L+4 14%
7H	S1	256	4.836	1.035	H→L 89%
	S7	190	6.522	0.063	H → L+3 23%, H-3 → L 20%
	S8	183	6.745	1.270	H-2 → L+1 52%, H-3 →L 20%
7C	S1	260	4.774	1.028	H→L 92%
	S9	184	6.735	1.386	H-2→L+156%, H-3→ L 16%
8M	S1	285	4.355	1.339	H → L 89%
	S12	183	6.774	1.505	H-3 → L+1 35% , H-2 → L+2 25%
	S23	161	7.691	0.313	H-3 → L+2 40%, H-1 → L+3 13%
8H	S1	273	4.534	1.080	H →L 89%

	S9	184	6.733	1.586	H-3 → L+1 48%, H-1 → L+3 23%
8C	S1	276	4.498	1.082	H → L 89%
	S9	185	6.705	1.328	H-3 → L+1 58%, H-1 → L+3 11%
9M	S1	303	4.089	1.568	H→L 88%
	S12	186	6.662	1.510	H-2→L+2 40%, H-3→L+1 24%, H-2→L+1 22%
9H	S1	292	4.243	1.311	H→L 85%
	S9	187	6.608	1.575	H-3→L+1 58%, H-2→L+3 24%
9C	S1	296	4.187	1.309	H→L 88%
	S10	187	6.622	1.270	H-3→L+1 38% , H-1 → L+3 31%, H→L+4 11%

Table S5. Calculated Overlap (Sr), D Index (Å), H index(Å), Variation of dipole moment with respect to ground state (Δμ, a.u.), Hole delocalization index (HDI), Electron delocalization index (EDI), Ghost-hunter index 1st and 2en, Coulomb attractive energy (E_{CA}) for iM, iH and iC compounds in the first excited state at the CAM-B3LYP/6-31G(d,p)/IEFPCM Level

	Sn	Sr	D (A)	Δμ	H	HDI	EDI	1st	E_{CA}
1M	S1	0.845	0.577	1.090	3.417	6.84	6.28	7.467	5.274
	S11	0.924	0.059	0.112	2.804	7.11	7.00	9.98	5.538
1H	S1	0.851	0.250	0.472	3.072	6.91	6.57	7.953	5.572
	S7	0.919	0.158	0.297	2.937	6.93	6.59	9.869	5.343
1C	S1	0.850	0.240	0.453	3.114	6.86	6.50	7.915	5.450
	S7	0.919	0.175	0.330	2.955	6.90	6.59	9.850	5.235
2M	S1	0.847	0.193	0.364	3.380	6.63	6.64	7.517	5.347
	S11	0.921	0.202	0.384	2.856	6.95	6.80	9.953	5.478
2H	S1	0.830	1.091	2.061	3.021	6.96	6.95	7.9	5.540
	S7	0.926	0.404	0.763	2.994	6.53	6.20	9.834	5.277
	S8	0.919	0.714	1.349	3.147	6.05	5.96	9.489	5.355
2C	S1	0.828	1.113	2.103	3.073	6.90	6.86	7.84	5.392
	S7	0.927	0.446	0.842	3.039	6.42	6.08	9.805	5.143
	S8	0.927	0.550	1.038	3.144	5.89	5.97	9.471	5.252
3M	S1	0.849	0.048	0.091	3.352	6.68	6.57	7.35	5.368
	S11	0.925	0.121	0.228	2.738	7.32	7.19	9.968	5.630
	S14	0.853	0.591	1.116	3.284	5.80	6.04	9.758	5.166
	S23	0.653	0.975	1.841	4.457	5.92	5.94	10.97	4.241
3H	S1	0.839	0.877	1.656	2.997	6.90	6.82	7.953	5.587
	S7	0.921	0.329	0.621	2.963	6.67	6.35	9.844	5.315
3C	S1	0.837	0.899	1.698	3.045	6.85	6.74	7.906	5.441
	S7	0.922	0.359	0.678	2.998	6.56	6.25	9.819	5.182
	S8	0.920	0.504	0.953	3.110	5.94	6.00	9.491	5.317
4M	S1	0.845	0.290	0.548	3.438	6.44	6.58	7.45	5.274
	S11	0.926	0.148	0.278	2.798	7.14	7.00	9.947	5.551
	S24	0.479	3.509	6.633	3.925	9.14	6.14	10.397	4.048
4H	S1	0.826	1.160	2.191	3.073	6.88	6.81	7.799	5.458

	S7	0.932	0.573	1.081	3.020	6.22	5.89	9.755	5.264
	S8	0.926	0.639	1.206	3.194	5.86	5.69	9.429	5.273
	S9	0.462	4.141	7.824	2.950	6.76	6.78	9.168	3.962
4C	S1	0.824	1.182	2.233	3.113	6.81	6.73	7.750	5.320
	S7	0.936	0.646	1.221	3.069	6.03	5.67	9.700	5.128
	S8	0.937	0.432	0.817	3.161	5.73	5.73	9.434	5.203
5M	S1	0.846	0.291	0.549	3.465	6.42	6.56	7.447	5.237
	S11	0.927	0.136	0.256	2.816	7.15	7.02	9.941	5.524
	S24	0.447	2.429	4.590	2.786	9.46	7.66	10.277	4.961
5H	S1	0.827	1.173	2.217	3.095	6.83	6.80	7.798	5.417
	S7	0.930	0.558	1.055	3.045	6.27	5.91	9.748	5.224
	S8	0.922	0.718	1.355	3.234	5.81	5.68	9.424	5.213
	S9	0.4702	4.117	7.780	2.978	6.75	6.72	9.177	3.968
5C	S1	0.8253	1.201	2.269	3.142	6.78	6.69	7.76	5.290
	S7	0.934	0.642	1.213	3.094	6.07	5.70	9.697	5.096
	S8	0.935	0.505	0.954	3.176	5.71	5.75	9.436	5.184
6M	S1	0.827	0.935	1.767	3.514	6.33	6.67	7.288	5.152
	S9	0.902	1.037	1.958	3.393	6.56	6.39	9.001	5.150
	S11	0.922	0.173	0.326	3.006	6.56	6.47	9.937	5.298
	S21	0.852	0.995	1.880	3.899	6.22	6.17	10.614	4.685
6H	S1	0.794	1.750	3.306	3.147	7.30	6.87	7.549	5.235
	S8	0.948	0.342	0.647	3.044	6.32	5.92	9.581	5.293
6C	S1	0.788	1.811	3.421	3.137	7.26	6.85	7.481	5.156
	S9	0.927	0.717	1.354	3.162	6.27	5.64	9.555	5.016
7M	S1	0.828	0.858	1.621	3.475	6.35	6.71	7.319	5.212
	S11	0.923	0.175	0.330	2.995	6.58	6.45	9.950	5.320
	S22	0.852	0.472	0.891	4.242	5.67	6.20	10.699	4.577
7H	S1	0.795	1.714	3.238	3.090	7.20	7.00	7.59	5.322
	S7	0.916	1.545	2.9193	3.312	5.85	5.01	9.351	4.923
	S8	0.950	0.460	0.869	3.049	6.17	5.84	9.527	5.323
7C	S1	0.790	1.754	3.314	3.096	7.21	6.92	7.522	5.224
	S9	0.924	0.872	1.647	3.172	6.17	5.51	9.503	5.003
8M	S1	0.798	1.591	3.005	3.536	6.47	6.70	7.017	5.055
	S12	0.921	0.079	0.150	3.034	6.56	6.42	9.962	5.272
	S23	0.232	4.043	7.640	2.119	9.56	9.11	9.862	3.787
8H	S1	0.751	2.249	4.251	3.11	7.67	6.98	7.176	5.158
	S9	0.930	0.183	0.3448	2.967	6.79	6.38	9.760	5.330
8C	S1	0.750	2.281	4.309	3.162	7.57	6.84	7.132	4.996
	S9	0.926	0.194	0.367	2.944	6.97	6.52	9.739	5.259
9M	S1	0.769	2.165	4.090	3.602	7.20	6.56	6.755	4.808
9H	S1	0.731	2.551	4.821	3.231	8.33	6.73	6.896	4.873
	S9	0.914	0.354	0.668	3.019	6.95	6.40	9.750	5.233

9C	S1	0.723	2.648	5.003	3.222	8.36	6.70	6.824	4.764
	S10	0.876	1.281	2.420	3.375	6.15	5.48	9.562	4.663

Table S6. Contribution of each fragment to hole and electron (I), Variation of population number of fragment (II) and intrafragment electron redistribution of fragment (III) calculated in tetrahydrofuranat the CAM-B3LYP/6-31G(d,p)/IEFPCM Level

Molecule	Sn	Frag	I		II	III
			Hole %	Electron%		
1M	S1	Frag1<br>Frag2<br>Frag3	30.15<br>68.70<br>1.15	22.16<br>75.63<br>2.21	-0.07988<br>0.06929<br>0.01059	0.06682<br>0.51953<br>0.00025
	S11	Frag1<br>Frag2<br>Frag3	1.38<br>98.13<br>0.48	2.04<br>97.45<br>0.51	0.00658<br>-0.00680<br>0.00022	0.00028<br>0.95637<br>0.00002
1H	S1	Frag1<br>Frag2<br>Frag3	5.32<br>92.3<br>2.38	9.35<br>87.35<br>3.30	0.04033<br>-0.04955<br>0.00922	0.00497<br>0.80624<br>0.00079
	S7	Frag1<br>Frag2<br>Frag3	1.88<br>97.65<br>0.47	5.29<br>94.12<br>0.59	0.03414<br>-0.03533<br>0.00119	0.00100<br>0.91910<br>0.00003
1C	S1	Frag1<br>Frag2<br>Frag3	6.30<br>91.31<br>2.38	10.59<br>86.16<br>3.25	0.04290<br>-0.05153<br>0.00864	0.00668<br>0.78678<br>0.00077
	S7	Frag1<br>Frag2<br>Frag3	3.41<br>96.12<br>0.47	6.84<br>92.62<br>0.54	0.03431<br>-0.03497<br>0.00066	0.00233<br>0.89030<br>0.00003
2M	S1	Frag1<br>Frag2<br>Frag3	27.77<br>70.39<br>1.84	25.01<br>73.95<br>1.04	-0.02766<br>0.03560<br>-0.00794	0.06954<br>0.52054<br>0.00019
	S11	Frag1<br>Frag2<br>Frag3	3.34<br>97.75<br>0.16	2.54<br>98.13<br>0.35	-0.00808<br>0.00610<br>0.00198	0.00085<br>0.95920<br>0.00001
2H	S1	Frag1<br>Frag2<br>Frag3	8.67<br>91.37<br>3.71	12.77<br>87.23<br>1.55	0.04485<br>-0.02324<br>-0.02160	0.01102<br>0.79698<br>0.00057
	S7	Frag1<br>Frag2<br>Frag3	1.62<br>97.63<br>0.75	6.36<br>93.35<br>0.29	0.04742<br>-0.04283<br>-0.00459	-0.04699<br>-0.00043<br>-0.00416
	S8	Frag1<br>Frag2<br>Frag3	6.00<br>90.75<br>3.25	13.87<br>84.64<br>1.19	0.07868<br>-0.06109<br>-0.01760	0.00833<br>0.76811<br>0.00048
2C	S1	Frag1<br>Frag2<br>Frag3	5.82<br>90.48<br>3.71	13.20<br>85.27<br>1.53	0.07385<br>-0.05209<br>-0.02176	0.00768<br>0.77147<br>0.00057
	S7	Frag1<br>Frag2<br>Frag3	3.85<br>96.25<br>0.88	8.57<br>91.43<br>0.32	0.04878<br>-0.04317<br>-0.00560	0.00321<br>0.88005<br>0.00003
	S8	Frag1<br>Frag2<br>Frag3	7.87<br>89.02<br>3.12	13.05<br>85.53<br>1.42	0.05185<br>-0.03490<br>-0.01695	0.01027<br>0.76136<br>0.00044

3M	S1	Frag1<br>Frag2<br>Frag3	28.19<br>71.33<br>0.48	24.80<br>74.62<br>0.58	-0.03390<br>0.03285<br>0.00105	0.06990<br>0.53229<br>0.00003
	S11	Frag1<br>Frag2<br>Frag3	2.23<br>97.68<br>0.08	1.91<br>97.86<br>0.23	-0.00325<br>0.00180<br>0.00145	0.00043<br>0.95597<br>0.00000
	S14	Frag1<br>Frag2<br>Frag3	17.31<br>82.13<br>0.56	6.07<br>93.52<br>0.41	-0.11239<br>0.11382<br>-0.00142	0.01051<br>0.76808<br>0.00002
	S23	Frag1<br>Frag2<br>Frag3	35.01<br>64.53<br>0.46	22.64<br>76.02<br>1.33	-0.12354<br>0.11485<br>0.00869	0.07930<br>0.49059<br>0.00006
3H	S1	Frag1<br>Frag2<br>Frag3	5.12<br>93.95<br>0.92	11.14<br>88.00<br>0.86	0.06013<br>-0.05948<br>-0.00065	0.00571<br>0.82682<br>0.00008
	S7	Frag1<br>Frag2<br>Frag3	1.59<br>98.23<br>0.19	5.85<br>93.92<br>0.23	0.04269<br>-0.04310<br>0.00041	0.00093<br>0.92255<br>0.00000
3C	S1	Frag1<br>Frag2<br>Frag3	6.09<br>93.09<br>0.91	12.96<br>86.40<br>0.86	0.06873<br>-0.06815<br>-0.00058	0.00789<br>0.80434<br>0.00008
	S7	Frag1<br>Frag2<br>Frag3	2.85<br>96.95<br>0.20	7.56<br>92.21<br>0.23	0.04711<br>-0.04738<br>0.00027	0.00215<br>0.89393<br>0.00000
	S8	Frag1<br>Frag2<br>Frag3	7.01<br>92.25<br>0.74	12.55<br>86.49<br>0.95	0.05545<br>-0.05760<br>0.00214	0.00880<br>0.79789<br>0.00007
4M	S1	Frag1<br>Frag2<br>Frag3	25.03<br>73.02<br>1.92	23.01<br>75.43<br>1.56	-0.02018<br>0.02403<br>-0.00385	0.05759<br>0.55078<br>0.00030
	S11	Frag1<br>Frag2<br>Frag3	2.30<br>97.41<br>0.29	1.89<br>97.23<br>0.89	-0.00419<br>-0.00177<br>0.00596	0.00043<br>0.94707<br>0.00003
	S24	Frag1<br>Frag2<br>Frag3	82.40<br>16.67<br>0.93	41.34<br>57.20<br>1.46	-0.41058<br>0.40529<br>0.00529	0.34064<br>0.09537<br>0.00014
4H	S1	Frag1<br>Frag2<br>Frag3	4.41<br>91.94<br>3.65	10.96<br>86.86<br>2.18	0.06549<br>-0.05081<br>-0.01467	0.00483<br>0.79863<br>0.00080
	S7	Frag1<br>Frag2<br>Frag3	1.62<br>97.20<br>1.18	6.81<br>92.13<br>1.06	0.05187<br>-0.05070<br>-0.00117	0.00110<br>0.89551<br>0.00013
	S8	Frag1<br>Frag2<br>Frag3	5.28<br>92.09<br>2.64	14.08<br>83.59<br>2.32	0.08806<br>-0.08494<br>-0.00311	0.00743<br>0.76980<br>0.00061
	S9	Frag1<br>Frag2<br>Frag3	17.63<br>80.78<br>1.59	91.39<br>8.5<br>0.11	0.73760<br>-0.72283<br>-0.01477	0.16115<br>0.06865<br>0.00002
4C	S1	Frag1<br>Frag2<br>Frag3	5.31<br>91.11<br>3.58	12.67<br>85.22<br>2.11	0.07367<br>-0.05891<br>-0.01477	0.00673<br>0.77642<br>0.00076
	S7	Frag1<br>Frag2<br>Frag3	2.80<br>95.85<br>1.36	8.66<br>90.15<br>1.19	0.05861<br>-0.05691<br>-0.00170	0.00242<br>0.86409<br>0.00016

	S8	Frag1<br>Frag2<br>Frag3	7.01<br>90.51<br>2.47	12.61<br>85.22<br>2.17	0.05600<br>-0.05297<br>-0.00303	0.00884<br>0.77132<br>0.00054
5M	S1	Frag1<br>Frag2<br>Frag3	24.8<br>72.71<br>2.47	22.92<br>75.30<br>1.77	-0.01898<br>0.02594<br>-0.00696	0.05690<br>0.54754<br>0.00044
	S11	Frag1<br>Frag2<br>Frag3	2.38<br>96.79<br>0.82	1.96<br>96.59<br>1.51	-0.00487<br>0.00201<br>0.00689	0.00045<br>0.93495<br>0.00012
	S24	Frag1<br>Frag2<br>Frag3	1.32<br>97.83<br>0.84	12.86<br>82.58<br>4.56	0.11540<br>-0.15256<br>0.03716	0.00170<br>0.80799<br>0.00039
5H	S1	Frag1<br>Frag2<br>Frag3	4.91<br>91.32<br>4.5	10.74<br>86.71<br>2.56	0.06552<br>-0.04611<br>-0.01941	0.00450<br>0.79175<br>0.00115
	S7	Frag1<br>Frag2<br>Frag3	1.52<br>96.72<br>1.76	6.59<br>91.49<br>1.92	0.05071<br>-0.05235<br>0.00164	0.00100<br>0.88493<br>0.00034
	S8	Frag1<br>Frag2<br>Frag3	5.46<br>91.19<br>3.35	14.62<br>82.88<br>2.5	0.09168<br>-0.08310<br>-0.00857	0.00789<br>0.75580<br>0.00084
	S9	Frag1<br>Frag2<br>Frag3	17.47<br>80.72<br>1.81	90.83<br>9.00<br>0.17	0.73365<br>-0.71728<br>-0.01637	0.15866<br>0.07263<br>0.00003
5C	S1	Frag1<br>Frag2<br>Frag3	5.13<br>90.58<br>4.28	12.71<br>84.79<br>2.50	0.07577<br>-0.05789<br>-0.01788	0.00653<br>0.76807<br>0.00107
	S7	Frag1<br>Frag2<br>Frag3	2.61<br>95.35<br>2.04	8.36<br>89.58<br>2.06	0.05754<br>-0.05772<br>0.00018	0.00218<br>0.85410<br>0.00042
	S8	Frag1<br>Frag2<br>Frag3	6.58<br>90.10<br>3.31	12.43<br>85.06<br>2.51	0.05848<br>-0.05043<br>-0.00805	0.00819<br>0.76642<br>0.00083
6M	S1	Frag1<br>Frag2<br>Frag3	19.99<br>73.71<br>6.29	21.73<br>76.24<br>2.03	0.01738<br>0.02527<br>-0.04266	0.04345<br>0.56197<br>0.00128
	S9	Frag1<br>Frag2<br>Frag3	6.95<br>83.87<br>9.18	13.22<br>82.96<br>3.82	0.06276<br>-0.00918<br>-0.05359	0.00918<br>0.69578<br>0.00351
	S11	Frag1<br>Frag2<br>Frag3	4.23<br>95.07<br>0.70	3.38<br>95.62<br>1.00	-0.00850<br>0.00547<br>0.00303	0.00143<br>0.90909<br>0.00007
	S21	Frag1<br>Frag2<br>Frag3	14.88<br>76.43<br>8.69	20.88<br>75.14<br>3.98	0.05998<br>-0.01286<br>-0.04712	0.03108<br>0.57425<br>0.00346
6H	S1	Frag1<br>Frag2<br>Frag3	3.90<br>85.31<br>10.79	11.32<br>85.83<br>2.85	0.07424<br>0.00520<br>-0.07944	0.00442<br>0.73219<br>0.00307
	S8	Frag1<br>Frag2<br>Frag3	4.72<br>91.86<br>3.42	10.41<br>88.17<br>1.42	0.05686<br>-0.03685<br>-0.02001	0.00491<br>0.80991<br>0.00049
6C	S1	Frag1<br>Frag2	4.21<br>85.25	12.39<br>84.88	0.08185<br>-0.00371	0.00521<br>0.72366

		Frag3	10.54	2.73	-0.07813	0.00287
	S9	Frag1	6.72	18.78	0.12054	0.01263
		Frag2	90.37	80.10	-0.10269	0.72385
		Frag3	2.91	1.12	-0.01785	0.00033
7 M	S1	Frag1	21.34	22.67	0.01323	0.04838
		Frag2	73.63	75.70	0.02075	0.55740
		Frag3	5.03	1.63	-0.03398	0.00082
	S11	Frag1	4.67	4.01	-0.00664	0.00187
		Frag2	94.99	95.37	0.00348	0.90590
		Frag3	0.34	0.62	0.00280	0.00002
	S22	Frag1	28.06	25.59	-0.02470	0.07181
		Frag2	63.35	70.06	0.06708	0.44381
		Frag3	8.59	4.35	-0.04237	0.00374
7H	S1	Frag1	3.98	11.70	0.07724	0.00466
		Frag2	87.81	86.01	-0.01800	0.75528
		Frag3	8.21	2.28	-0.05924	0.00187
	S7	Frag1	3.05	18.64	0.15591	0.00569
		Frag2	91.34	79.95	-0.11385	0.73029
		Frag3	5.61	1.4	-0.04206	0.00079
	S8	Frag1	5.09	11.36	0.06277	0.00578
		Frag2	91.39	87.30	-0.04085	0.79788
		Frag3	3.52	1.33	-0.02193	0.00047
7C	S1	Frag1	4.28	12.82	0.08534	0.00549
		Frag2	87.33	84.91	-0.02418	0.74154
		Frag3	8.39	2.27	-0.06116	0.00190
	S9	Frag1	7.60	21.48	0.13879	0.01633
		Frag2	89.42	77.52	-0.11892	0.69317
		Frag3	2.98	1.00	-0.01987	0.00030
8M	S1	Frag1	14.03	19.26	0.05235	0.02702
		Frag2	75.10	78.04	0.02942	0.58613
		Frag3	10.87	2 .69	-0.08177	0.00293
	S12	Frag1	3.91	3.66	-0.00260	0.00143
		Frag2	95.98	95.95	-0.00214	0.92090
		Frag3	0.50	0.97	0.00474	0.00005
	S23	Frag1	2.06	1.70	-0.00361	0.00035
		Frag2	97.63	97.11	-0.00525	0.94803
		Frag3	0.31	1.19	0.00886	0.00004
8H	S1	Frag1	2.78	10.54	0.07761	0.00293
		Frag2	81.25	85.99	0.04733	0.69867
		Frag3	15.97	3.47	-0.12499	0.00554
	S9	Frag1	3.05	7.37	0.04315	0.00225
		Frag2	95.50	91.76	-0.03739	0.87637
		Frag3	1.45	0.87	-0.00576	0.00013
8C	S1	Frag1	3.30	12.52	0.09222	0.00413
		Frag2	81.02	84.04	0.03019	0.68094
		Frag3	15.68	3.44	-0.12241	0.00539
	S9	Frag1	5.25	10.34	0.05188	0.00533
		Frag2	93.56	88.91	-0.04650	0.83179
		Frag3	1.29	0.75	-0.00538	0.00100
9M	S1	Frag1	9.87	17.23	0.07360	0.01700
		Frag2	69.74	78.40	0.08659	0.54671

		Frag3	20.39	4.38	-0.16019	0.00892
9H	S1	Frag1<br>Frag2<br>Frag3	2.35<br>72.19<br>25.46	10.16<br>84.17<br>5.67	0.07809<br>0.11983<br>-0.19792	0.00239<br>0.60767<br>0.01443
	S9	Frag1<br>Frag2<br>Frag3	2.64<br>95.80<br>1.56	9.51<br>89.45<br>1.03	0.06870<br>-0.06345<br>-0.00525	0.00251<br>0.85698<br>0.00016
9C	S1	Frag1<br>Frag2<br>Frag3	2.42<br>71.96<br>25.62	11.52<br>83.19<br>5.30	0.09098<br>0.11226<br>-0.20323	0.00279<br>0.59863<br>0.01357
	S10	Frag1<br>Frag2<br>Frag3	3.22<br>92.18<br>4.61	19.12<br>79.75<br>1.13	0.15903<br>-0.12423<br>-0.03479	0.00615<br>0.73514<br>0.00052

Table S7. Intrinsic charge transfer percentage, CT(%), Intrinsic local excitation percentage, LE(%) and Transferred electrons between fragments, calculated for the electronic transition $S_{0\rightarrow i}$ of the iM, iH and iC compounds at the CAM-B3LYP/6-31G(d,p)/IEFPCM Level in tetrahydrofuran

	Sn	CT(%)	LE(%):	Transferred electrons between fragments		
1M	S1	41.339	58.661	1 -> 2: 0.22802<br>1 -> 3: 0.00667<br>2-> 3: 0.01519	1<- 2: 0.15225<br>1 <- 3: 0.00255<br>2 <- 3: 0.00871	Net 1->2: 0.07577<br>Net 1->3: 0.00411<br>Net 2->3: 0.00648
	S11	4.333	95.667	1-> 2: 0.01347<br>1 -> 3: 0.00007<br>2 -> 3: 0.00496	1<- 2: 0.02001<br>1 <- 3: 0.00010<br>2 <- 3: 0.00471	Net 1->2: -0.00655<br>Net 1->3: -0.00003<br>Net 2 ->3: 0.00025
1H	S1	18.80	81.199	1 -> 2: 0.04645<br>1 -> 3: 0.00176<br>2 -> 3: 0.03048	1<- 2: 0.08631<br>1 <- 3: 0.00223<br>2 <- 3: 0.02079	Net 1->2: -0.03986<br>Net 1->3: -0.00047<br>Net 2->3: 0.00969
	S7	7.988	92.012	1 -> 2: 0.01770<br>1 -> 3: 0.00011<br>2 -> 3: 0.00572	1<- 2: 0.05170<br>1 <- 3: 0.00025<br>2 <- 3: 0.00440	Net 1->2: -0.03400<br>Net 1->3: -0.00014<br>Net 2->3: 0.00133
1C	S1	20.577	79.423	1 -> 2: 0.05431<br>1 -> 3: 0.00205<br>2 -> 3: 0.02964	1<- 2: 0.09672<br>1 <- 3: 0.00252<br>2 <- 3: 0.02053	Net 1->2: -0.04242<br>Net 1->3: -0.00048<br>Net 2->3: 0.00911
	S7	10.73	89.266	1 -> 2: 0.03156<br>1 -> 3: 0.00018<br>2 -> 3: 0.00518	1<- 2: 0.06572<br>1 <- 3: 0.00032<br>2 <- 3: 0.00438	Net 1->2: -0.03417<br>Net 1->3: -0.00014<br>Net 2->3: 0.00080
	S1	40.98	59.019	1 -> 2: 0.20538<br>1 -> 3: 0.00290<br>2 -> 3: 0.00734	1<- 2: 0.17603<br>1 <- 3: 0.00459<br>2 <- 3: 0.01358	Net 1->2: 0.02935<br>Net 1->3: -0.00170<br>Net 2->3: -0.00624
	S11	6.274	96.006	1 -> 2: 0.03280<br>1 -> 3: 0.00012<br>2 -> 3: 0.00344	1<- 2: 0.02480<br>1 <- 3: 0.00004<br>2 <- 3: 0.00154	Net 1->2: 0.00800<br>Net 1->3: 0.00008<br>Net 2->3: 0.00190
2H	S1	24.459	80.858	1 -> 2: 0.07528<br>1 -> 3: 0.00134<br>2 -> 3: 0.01416	1<- 2: 0.11672<br>1 <- 3: 0.00474<br>2 <- 3: 0.03236	Net 1->2: -0.04144<br>Net 1->3: -0.00340<br>Net 2->3: -0.01820
	S7	8.757	91.243	1 -> 2: 0.01514<br>1 -> 3: 0.00005<br>2 -> 3: 0.00281	1<- 2: 0.06213<br>1 <- 3: 0.00048<br>2 <- 3: 0.00697	Net 1->2: -0.04699<br>Net 1->3: -0.00043<br>Net 2->3: -0.00416
	S8	22.308	77.692	1 -> 2: 0.05080<br>1 -> 3: 0.00089<br>2 -> 3: 0.01351	1<- 2: 0.12587<br>1 <- 3: 0.00451<br>2 <- 3: 0.02749	Net 1-> 2: -0.07507<br>Net 1->3: -0.00361<br>Net 2->3: -0.01398
2C	S1	22.02	77.972	1 -> 2: 0.04959<br>1 -> 3: 0.00089<br>2 -> 3: 0.01306	1<- 2: 0.11943<br>1 <- 3: 0.00489<br>2 <- 3: 0.03162	Net 1->2: -0.06984<br>Net 1->3: -0.00400<br>Net 2->3: -0.01776
	S7	12.872	88.329	1 -> 2: 0.03430<br>1 -> 3: 0.00012<br>2 -> 3: 0.00307	1<- 2: 0.08244<br>1 <- 3: 0.00075<br>2<- 3: 0.00804	Net 1->2: -0.04814<br>Net 1->3: -0.00063<br>Net 2->3: -0.00497
	S8	22.794	77.206	1 -> 2: 0.06727<br>1 -> 3: 0.00112<br>2 -> 3: 0.01265	1 <- 2: 0.11617<br>1 <- 3: 0.00407<br>2 <- 3: 0.02665	Net 1->2: -0.04890<br>Net 1->3: -0.00295<br>Net 2->3: -0.01400
3M	S1	39.778	60.222	1 -> 2: 0.21034<br>1 -> 3: 0.00164<br>2 -> 3: 0.00415	1<- 2: 0.17690<br>1 <- 3: 0.00118<br>2 <- 3: 0.00356	Net 1->2: 0.03344<br>Net 1->3: 0.00046<br>Net 2->3: 0.00059
	S11	4.361	95.639	1 -> 2: 0.02185	1 <- 2: 0.01863	Net 1->2: 0.00322

				1 -> 3: 0.00005<br>2 -> 3: 0.00224	1 <- 3: 0.00002<br>2 <- 3: 0.00082	Net 1->3: 0.00004<br>Net 2->3: 0.00142
	S14	22.139	77.861	1 -> 2: 0.16187<br>1 -> 3: 0.00072<br>2 -> 3: 0.00340	1 <- 2: 0.04986<br>1 <- 3: 0.00034<br>2 <- 3: 0.00521	Net 1->2: 0.11202<br>Net 1->3: 0.00038<br>Net 2->3: -0.00180
	S23	43.005	56.995	1 -> 2: 0.26611<br>1 -> 3: 0.00465<br>2 -> 3: 0.00857	1<- 2: 0.14619<br>1 <- 3: 0.00104<br>2 <- 3: 0.00349	Net 1->2: 0.11993<br>Net 1->3: 0.00361<br>Net 2->3: 0.00508
3H	S1	16.740	83.260	1 -> 2: 0.04510<br>1 -> 3: 0.00044<br>2 -> 3: 0.00807	1<- 2: 0.10463<br>1<- 3: 0.00103<br>2<- 3: 0.00813	Net 1->2: -0.05954<br>Net 1->3: -0.00059<br>Net 2->3: -0.00006
	S7	7.652	92.348	1 -> 2: 0.01489<br>1 -> 3: 0.00004<br>2 -> 3: 0.00223	1<- 2: 0.05751<br>1<- 3: 0.00011<br>2<-3: 0.00175	Net 1->2: -0.04262<br>Net 1->3: -0.00007<br>Net 2->3: 0.00048
3C	S1	19.081	81.231	1 -> 2: 0.05260<br>1 -> 3: 0.00052<br>2 -> 3: 0.00796	1 <- 2: 0.12067<br>1 <- 3: 0.00118<br>2 <- 3: 0.00788	Net 1->2: -0.06807<br>Net 1->3: -0.00066<br>Net 2->3: 0.00008
	S7	10.391	89.609	1 -> 2: 0.02627<br>1 -> 3: 0.00007<br>2 -> 3: 0.00225	1<- 2: 0.07329<br>1 <- 3: 0.00015<br>2 <- 3: 0.00189	Net 1->2: -0.04702<br>Net 1->3: -0.00009<br>Net 2->3: 0.00036
	S8	19.324	80.676	1 -> 2: 0.06062<br>1 -> 3: 0.00067<br>2 -> 3: 0.00880	1 <- 2: 0.11581<br>1 <- 3: 0.00093<br>2 <- 3: 0.00640	Net 1->2: -0.05519<br>Net 1->3: -0.00026<br>Net 2->3: 0.00240
4M	S1	39.132	60.868	1 -> 2: 0.18878<br>1 -> 3: 0.00391<br>2 -> 3: 0.01141	1 <- 2: 0.16803<br>1 <- 3: 0.00448<br>2 <- 3: 0.01469	Net 1->2: 0.02075<br>Net 1->3: -0.00057<br>Net 2->3: -0.00328
	S11	5.247	94.753	1 -> 2: 0.02241<br>1 -> 3: 0.00020<br>2 -> 3: 0.00862	1 <- 2: 0.01837<br>1 <- 3: 0.00005<br>2 <- 3: 0.00281	Net 1->2: 0.00404<br>Net 1->3: 0.00015<br>Net 2->3: 0.00581
	S24	56.385	43.615	1 -> 2: 0.47134<br>1 -> 3: 0.01201<br>2 -> 3: 0.00243	1 <- 2: 0.06893<br>1 <- 3: 0.00384<br>2 <- 3: 0.00531	Net 1->2: 0.40241<br>Net 1->3: 0.00817<br>Net 2->3: -0.00288
4H	S1	19.574	80.426	1 -> 2: 0.03828<br>1 -> 3: 0.00096<br>2 -> 3: 0.02006	1 <- 2: 0.10073<br>1 <- 3: 0.00400<br>2 <- 3: 0.03170	Net 1->2: -0.06245<br>Net 1->3: -0.00304<br>Net 2->3: -0.01164
	S7	10.326	89.674	1 -> 2: 0.01492<br>1 -> 3: 0.00017<br>2 -> 3: 0.01033	1 <- 2: 0.06616<br>1 <- 3: 0.00080<br>2 <- 3: 0.01087	Net 1->2: -0.05124<br>Net 1->3: -0.00063<br>Net 2->3: -0.00054
	S8	22.215	77.785	1 -> 2: 0.04411<br>1 -> 3: 0.00123<br>2 -> 3: 0.02140	1 <- 2: 0.12968<br>1 <- 3: 0.00371<br>2 <- 3: 0.02203	Net 1->2: -0.08557<br>Net 1->3: -0.00248<br>Net 2->3: -0.00063
	S9	77.019	22.981	1 -> 2: 0.01498<br>1 -> 3: 0.00019<br>2 -> 3: 0.00089	1 <- 2: 0.73828<br>1 <- 3: 0.01450<br>2 <- 3: 0.00135	Net 1->2: -0.72329<br>Net 1->3: -0.01431<br>Net 2->3: -0.00046
4C	S1	21.610	78.390	1 -> 2: 0.04522<br>1 -> 3: 0.00112<br>2 -> 3: 0.01920	1 <- 2: 0.11547<br>1 <- 3: 0.00454<br>2 <- 3: 0.03054	Net 1->2: -0.07025<br>Net 1->3: -0.00342<br>Net 2->3: -0.01134
	S7	13.333	86.667	1 -> 2: 0.02521<br>1 -> 3: 0.00033<br>2 -> 3: 0.01139	1 <- 2: 0.08267<br>1 <- 3: 0.00118<br>2 <- 3: 0.01225	Net 1->2: -0.05776<br>Net 1->3: -0.00084<br>Net 2->3: -0.00085
	S8	21.930	78.070	1 -> 2:0.05975<br>1 -> 3: 0.00152<br>2 -> 3: 0.01966	1<- 2: 0.11415<br>1<- 3: 0.00312<br>2<- 3: 0.02109	Net 1->2: -0.05440<br>Net 1->3: -0.00160<br>Net 2->3: -0.00143
5M	S1	39.512	60.488	1 -> 2: 0.18691	1 <- 2: 0.16667	Net 1->2: 0.02024

				1 -> 3: 0.00440<br>2 -> 3: 0.01289	1 <- 3: 0.00566<br>2 <- 3: 0.01859	Net 1->3: -0.00126<br>Net 2->3: -0.00570
	S11	6.447	93.553	1 -> 2: 0.02303<br>1 -> 3: 0.00036<br>2 -> 3: 0.01462	1 <- 2: 0.01836<br>1 <- 3: 0.00016<br>2 <- 3: 0.00794	Net 1->2: 0.00467<br>Net 1->3: 0.00020<br>Net 2->3: 0.00668
	S24	19.002	80.998	1 -> 2: 0.01091<br>1 -> 3: 0.00060<br>2 -> 3: 0.04462	1 <- 2: 0.12583<br>1 <- 3: 0.00109<br>2 <- 3: 0.00697	Net 1->2: -0.11492<br>Net 1->3: -0.00048<br>Net 2->3: 0.03764
5H	S1	20.260	79.740	1 -> 2: 0.03631<br>1 -> 3: 0.00107<br>2 -> 3: 0.02334	1 <- 2: 0.09807<br>1 <- 3: 0.00483<br>2 <- 3: 0.03899	Net 1->2: -0.06176<br>Net 1->3: -0.00376<br>Net 2->3: -0.01565
	S7	11.373	88.627	1 -> 2: 0.01388<br>1 -> 3: 0.00029<br>2 -> 3: 0.01858	1 <- 2: 0.06373<br>1 <- 3: 0.00116<br>2 <- 3: 0.01608	Net 1->2: -0.04985<br>Net 1->3: -0.00087<br>Net 2->3: -0.00250
	S8	23.538	76.462	1 -> 2: 0.04522<br>1 -> 3: 0.00136<br>2 -> 3: 0.02275	1 <- 2: 0.13336<br>1 <- 3: 0.00490<br>2 <- 3: 0.02779	Net1-> 2: -0.08813<br>Net 1->3: -0.00354<br>Net 2->3: -0.00503
	S9	23.132	76.868	1 -> 2: 0.01572<br>1 -> 3: 0.00030<br>2 -> 3: 0.00138	1 <-2: 0.73324<br>1 <-3: 0.01642<br>2 <-3: 0.00163	Net 1->2: -0.71752<br>Net 1->3: -0.01613<br>Net 2->3: -0.00025
5C	S1	22.433	77.567	1 -> 2: 0.04353<br>1 -> 3: 0.00128<br>2 -> 3: 0.02261	1 <- 2: 0.11514<br>1 <- 3: 0.00545<br>2 <- 3: 0.03633	Net1-> 2: -0.07161<br>Net1-> 3: -0.00416<br>Net 2->3: -0.01371
	S7	14.330	85.670	1 -> 2: 0.02337<br>1 -> 3: 0.00054<br>2 -> 3: 0.01964	1 <- 2: 0.07974<br>1 <- 3: 0.00171<br>2 <- 3: 0.01829	Net 1->2: -0.05637<br>Net 1->3: -0.00117<br>Net 2->3: 0.00135
	S8	22.456	77.544	1 -> 2: 0.05601<br>1 -> 3: 0.00165<br>2 -> 3: 0.02259	1 <- 2: 0.11202<br>1 <- 3: 0.00412<br>2 <- 3: 0.02817	Net 1->2: -0.05601<br>Net 1->3: -0.00247<br>Net 2->3: -0.00558
6M	S1	39.330	60.670	1 -> 2: 0.15243<br>1 -> 3: 0.00406<br>2 -> 3: 0.01496	1 <- 2: 0.16019<br>1 <- 3: 0.01368<br>2 <- 3: 0.04799	Net 1->2: -0.00776<br>Net 1->3: -0.00962<br>Net 2->3: -0.03303
	S9	29.152	70.848	1 -> 2: 0.05762<br>1 -> 3: 0.00265<br>2 -> 3: 0.03205	1 <- 2: 0.11090<br>1 <- 3: 0.01214<br>2 <- 3: 0.07616	Net 1->2: -0.05328<br>Net 1->3: -0.00948<br>Net 2->3: -0.04410
	S11	8.942	91.058	1 -> 2: 0.04040<br>1 -> 3: 0.00042<br>2 -> 3: 0.00955	1<- 2: 0.03209<br>1 <- 3: 0.00024<br>2 <- 3: 0.00671	Net 1->2: 0.00831<br>Net 1->3: 0.00019<br>Net 2->3: 0.00284
	S21	39.121	60.879	1 -> 2: 0.11183<br>1 -> 3: 0.00592<br>2 -> 3: 0.03041	1 <- 2: 0.15959<br>1 <- 3: 0.01815<br>2 <- 3: 0.06531	Net 1->2: -0.04776<br>Net 1->3: -0.01223<br>Net 2->3: -0.03489
6H	S1	26.032	73.968	1 -> 2: 0.03348<br>1 -> 3: 0.00111<br>2 -> 3: 0.02429	1 <- 2: 0.09661<br>1 <- 3: 0.01222<br>2 <- 3: 0.09262	Net 1->2: -0.06313<br>Net 1->3: -0.01111<br>Net 2->3: -0.06833
	S8	18.469	81.531	1 -> 2: 0.04163<br>1 -> 3: 0.00067<br>2 -> 3: 0.01305	1 <- 2: 0.09561<br>1 <- 3: 0.00356<br>2 <- 3: 0.03017	Net 1->2: -0.05397<br>Net 1->3: -0.00289<br>Net 2->3: -0.01712
6C	S1	26.825	73.175	1 -> 2: 0.03571<br>1 -> 3: 0.00115<br>2 -> 3: 0.02324	1 <- 2: 0.10564<br>1 <- 3: 0.01306<br>2 <- 3: 0.08946	Net 1->2: -0.06993<br>Net 1->3: -0.01191<br>Net 2->3: -0.06622
	S9	26.320	73.680	1 -> 2: 0.05386<br>1 -> 3: 0.00076<br>2 -> 3: 0.01015	1 <- 2: 0.16969<br>1 <- 3: 0.00546<br>2 <- 3: 0.02329	Net 1->2: -0.11383<br>Net 1->3: -0.00470<br>Net 2->3: -0.01314
7M	S1	39.340	60.660	1->2: 0.16158	1 <- 2: 0.16689	Net 1->2: -0.00531

				1->3: 0.00348<br>2->3: 0.01200	1 <- 3: 0.01139<br>2 <- 3: 0.03806	Net 1->3: -0.00792<br>Net 2->3: -0.02606
	S11	9.220	90.780	1-> 2: 0.04458<br>1-> 3: 0.00029<br>2-> 3: 0.00588	1 <- 2: 0.03809<br>1 <- 3: 0.00014<br>2 <- 3: 0.00323	Net 1->2: 0.00649<br>Net 1->3: 0.00015<br>Net 2->3: 0.00265
	S22	48.064	51.936	1-> 2: 0.19659<br>1-> 3: 0.01221<br>2-> 3: 0.02757	1 <- 2: 0.16212<br>1 <- 3: 0.02198<br>2 <- 3: 0.06017	Net 1->2: 0.03447<br>Net 1->3: -0.00977<br>Net 2->3: -0.03260
7H	S1	23.819	76.181	1 -> 2: 0.03424<br>1 -> 3: 0.00091<br>2-> 3: 0.02005	1 <- 2: 0.10278<br>1 <- 3: 0.00961<br>2 <- 3: 0.07059	Net 1->2: -0.06854<br>Net 1->3: -0.00870<br>Net 2->3: -0.05054
7C	S1	25.106	74.894	1 -> 2: 0.03637<br>1 -> 3: 0.00097<br>2 -> 3: 0.01983	1 <- 2:0.11193<br>1 <- 3:0.01075<br>2 <- 3:0.07121	Net 1->2: -0.07556<br>Net 1->3: -0.00978<br>Net 2->3: -0.05138
	S9	29.020	70.980	1 -> 2: 0.05893<br>1 -> 3: 0.00076<br>2 -> 3: 0.00891	1 <- 2: 0.19207<br>1 <- 3: 0.00641<br>2 <- 3: 0.02313	Net 1->2: -0.13314<br>Net 1->3: -0.00565<br>Net 2->3: -0.01422
8M	S1	38.392	61.608	1 -> 2: 0.10948<br>1 -> 3: 0.00378<br>2 -> 3: 0.02023	1 <- 2: 0.14467<br>1 <- 3: 0.02094<br>2 <- 3: 0.08483	Net 1->2: -0.03519<br>Net 1->3: -0.01716<br>Net 2->3: -0.06461
	S12	8.684	91.316	1 -> 2: 0.03735<br>1 -> 3: 0.00038<br>2 -> 3: 0.00926	1 <- 2: 0.03499<br>1 <- 3: 0.00018<br>2 <- 3: 0.00472	Net 1->2: 0.00232<br>Net 1->3: 0.00020<br>Net 2->3: 0.00453
	S23	5.158	94.842	1 -> 2: 0.02004<br>1 -> 3: 0.00025<br>2 -> 3: 0.01165	1 <-2: 0.01662<br>1 <-3: 0.00005<br>2 <- 3: 0.00298	Net 1->2: 0.00342<br>Net 1->3: 0.00019<br>Net 2->3: 0.00867
8H	S1	29.286	70.714	1 -> 2: 0.02390<br>1 -> 3: 0.00096<br>2 -> 3: 0.02820	1 <- 2: 0.08565<br>1 <- 3: 0.01683<br>2 <- 3: 0.13732	Net 1->2: -0.06174<br>Net 1->3: -0.01587<br>Net 2->3: -0.10912
	S9	12.125	87.875	1 -> 2: 0.02799<br>1 -> 3: 0.00027<br>2 -> 3: 0.00831	1 <- 2: 0.07035<br>1 <- 3: 0.00107<br>2 <- 3: 0.01327	Net 1 ->2: -0.04235<br>Net 1 ->3: -0.00080<br>Net 2 ->3: -0.00496
8C	S1	30.954	69.046	1 -> 2: 0.02770<br>1 -> 3: 0.00113<br>2 -> 3: 0.02787	1 <- 2: 0.10142<br>1 <- 3: 0.01963<br>2 <- 3: 0.13179	Net 1-> 2: -0.07372<br>Net 1 ->3: -0.01849<br>Net 2 ->3: -0.10392
	S9	16.278	83.72	1 -> 2: 0.02685<br>1 -> 3: 0.00012<br>2 -> 3: 0.00395	1 <- 2: 0.07177<br>1<- 3: 0.00080<br>2 <- 3: 0.00983	Net 1->2: -0.04492<br>Net 1->3: -0.00068<br>Net 2->3: -0.00587
9M	S1	42.736	57.264	1 -> 2: 0.07737<br>1 -> 3: 0.00432<br>2 -> 3: 0.03051	1 <- 2: 0.12015<br>1 <- 3: 0.03514<br>2 <- 3: 0.15988	Net 1->2: -0.04278<br>Net 1->3: -0.03082<br>Net 2->3: -0.12937
9H	S1	37.551	62.449	1 -> 2: 0.01977<br>1 -> 3: 0.00133<br>2 -> 3: 0.04092	1 <- 2: 0.07333<br>1 <- 3: 0.02586<br>2 <- 3: 0.21431	Net 1->2: -0.05336<br>Net 1->3: -0.02453<br>Net 2 ->3: -0.17339
	S9	14.034	85.966	1 -> 2: 0.02365<br>1 -> 3: 0.00027<br>2 -> 3: 0.00988	1 <- 2: 0.09114<br>1 <- 3: 0.00148<br>2 <- 3: 0.01392	Net 1-> 2: -0.06749<br>Net 1->3: -0.00121<br>Net 2->3: -0.00404
9C	S1	38.502	61.498	1 -> 2: 0.02013<br>1 -> 3: 0.00128<br>2 -> 3: 0.03811	1 <- 2: 0.08288<br>1 <- 3: 0.02951<br>2 <- 3: 0.21311	Net 1->2: -0.06275<br>Net 1->3: -0.02822<br>Net 2->3: -0.17501
	S10	25.819	74.181	1 -> 2: 0.02566<br>1 -> 3: 0.00036<br>2 -> 3: 0.01039	1 <- 2: 0.17624<br>1 <- 3: 0.00881<br>2 <- 3: 0.03674	Net 1->2: -0.15058<br>Net 1->3: -0.00844<br>Net 2->3: -0.02635

Table S8. Calculated dynamic polarizability (α) and polarizability anisotropy (Δα)

	polarizability (α)				polarizability anisotropy (Δα)			
	1906	1340	1064	556	1906	1340	1064	556
1M	249	250	252	270	267	266	270	310
1H	290	292	293	306	216	219	222	246
IC	348	349	351	365	203	205	208	232
2M	235	236	238	254	249	252	256	292
2H	277	278	280	292	204	207	209	232
2C	334	336	338	351	190	192	195	218
3M	234	236	238	254	248	252	256	291
3H	276	278	279	291	204	206	209	231
3C	334	336	337	351	189	192	194	217
4M	251	253	255	273	270	274	278	319
4H	293	294	296	310	225	227	231	257
4C	351	352	354	370	211	214	217	244
5M	289	291	294	313	289	293	298	342
5H	331	333	334	349	244	246	250	279
5C	389	389	393	409	231	231	238	267
6M	258	260	262	282	284	288	293	338
6H	300	310	303	318	237	240	243	273
6C	358	360	361	378	227	229	233	264
7M	243	244	247	265	267	271	276	318
7H	284	286	288	302	220	223	226	253
7C	343	344	346	342	209	212	215	209
8M	253	255	257	279	291	295	302	352
8H	295	297	299	315	247	250	254	290
8C	353	355	357	376	234	237	241	278
9M	286	289	292	318	328	333	340	406
9H	327	329	332	353	279	284	289	337
9C	386	389	391	415	271	276	281	332

List of Figures

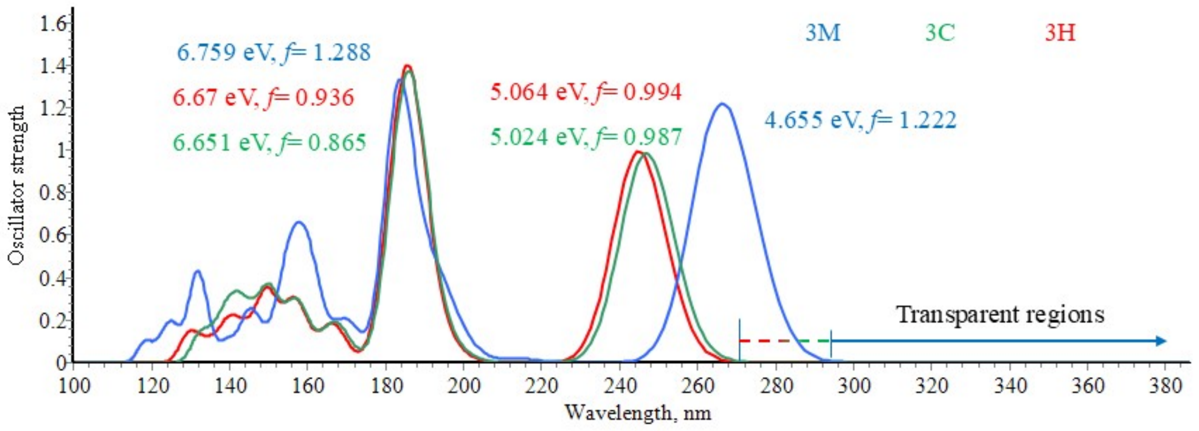
3M
3C
3H
6.759 eV, f= 1.288
6.67 eV, f= 0.936
6.651 eV, f= 0.865
5.064 eV, f= 0.994
5.024 eV, f= 0.987
4.655 eV, f= 1.222
Transparent regions
Oscillator strength
Wavelength, nm

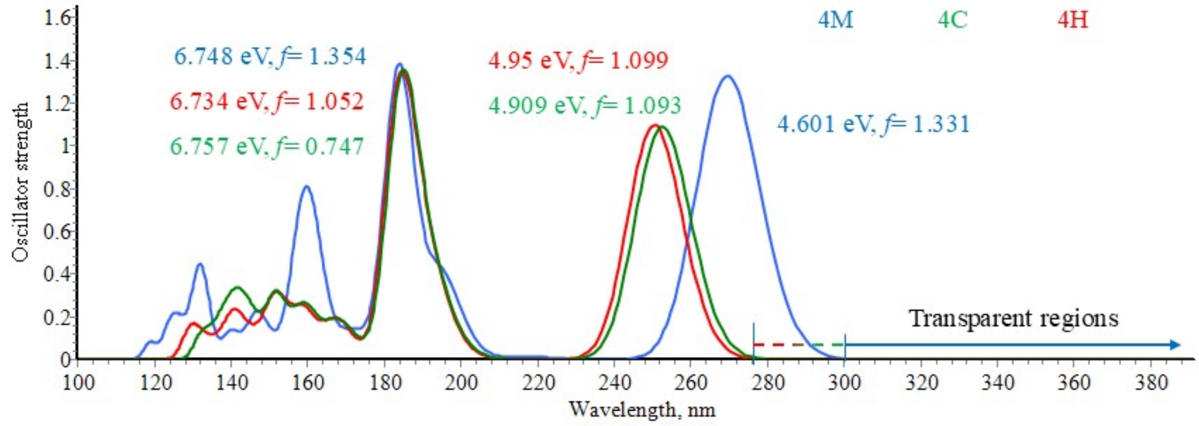
4M
4C
4H
6.748 eV, f= 1.354
6.734 eV, f= 1.052
6.757 eV, f= 0.747
4.95 eV, f= 1.099
4.909 eV, f= 1.093
4.601 eV, f= 1.331
Transparent regions
Oscillator strength
Wavelength, nm

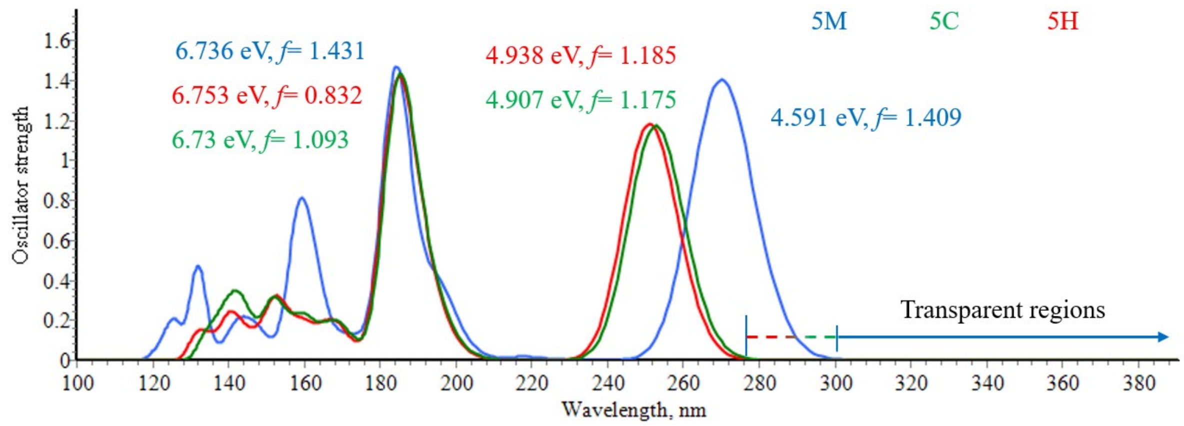
5M
5C
5H
6.736 eV, f= 1.431
6.753 eV, f= 0.832
6.73 eV, f= 1.093
4.938 eV, f= 1.185
4.907 eV, f= 1.175
4.591 eV, f= 1.409
Transparent regions
Oscillator strength
Wavelength, nm

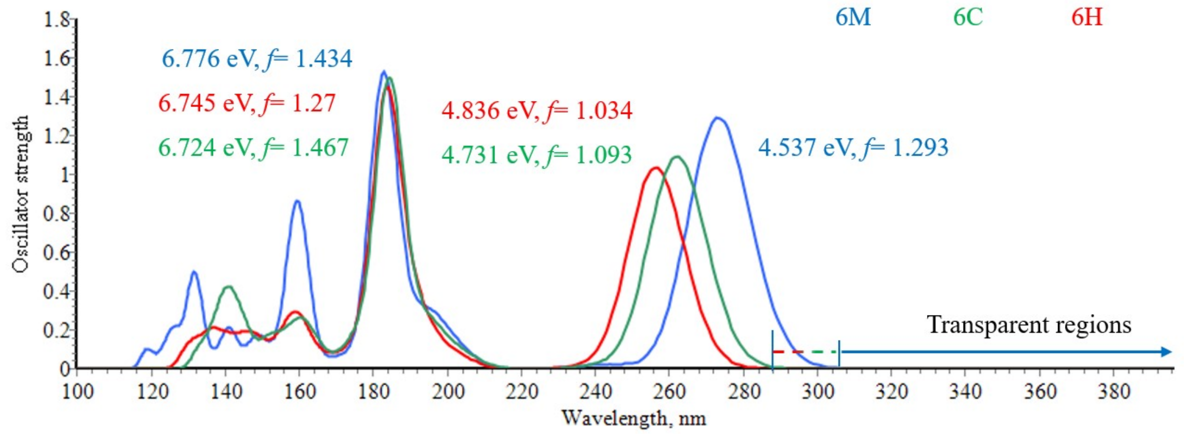
6M
6C
6H
6.776 eV, f= 1.434
6.745 eV, f= 1.27
6.724 eV, f= 1.467
4.836 eV, f= 1.034
4.731 eV, f= 1.093
4.537 eV, f= 1.293
Transparent regions
Oscillator strength
Wavelength, nm

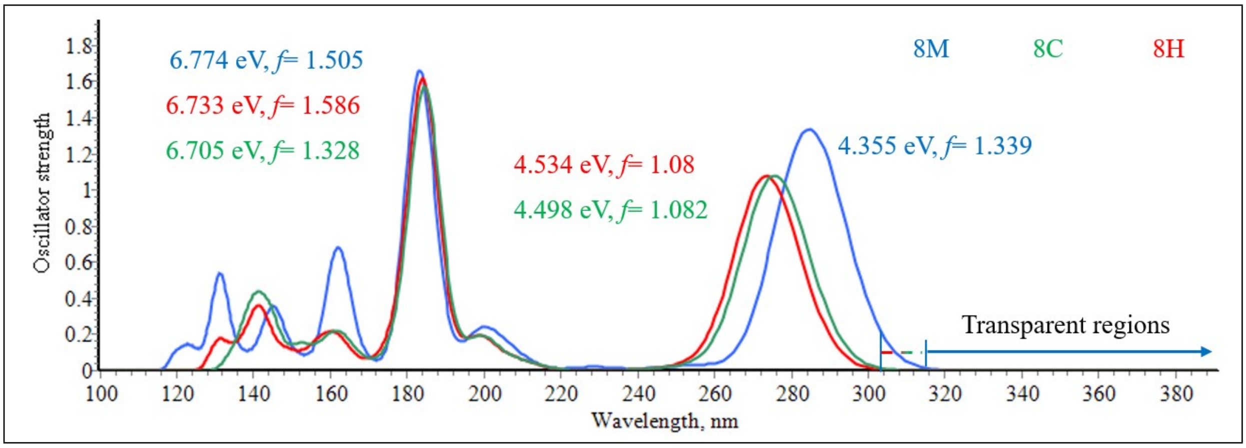

Figure S1. Simulated absorption spectrum of the title compounds

**1C**	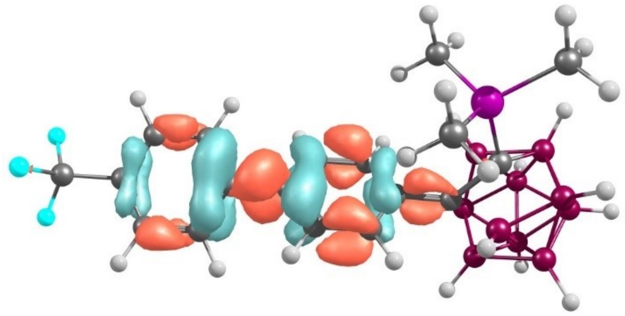 **S1**	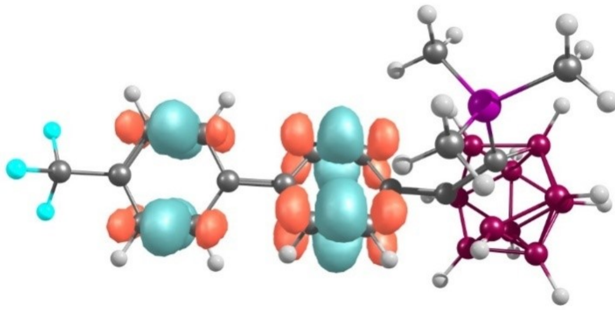 **S7**
**1M**	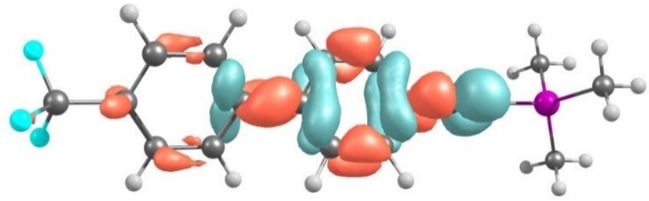 **S1**	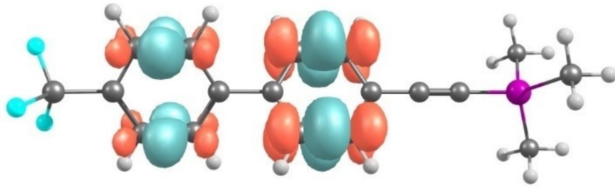 **S11**
**1H**	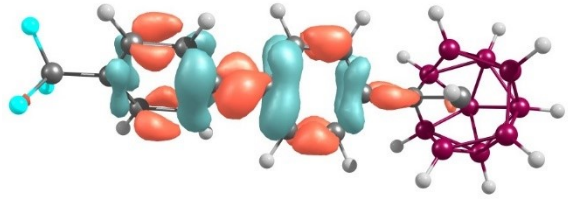 **S1**	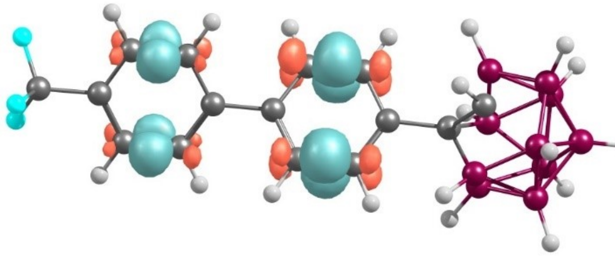 **S7**
**2C**	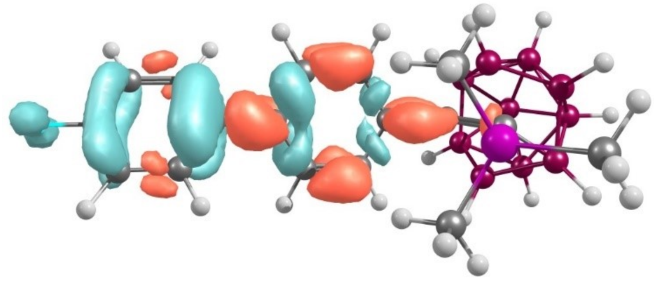 **S1**	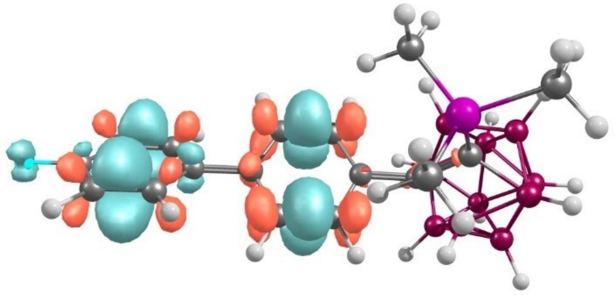

		S7
	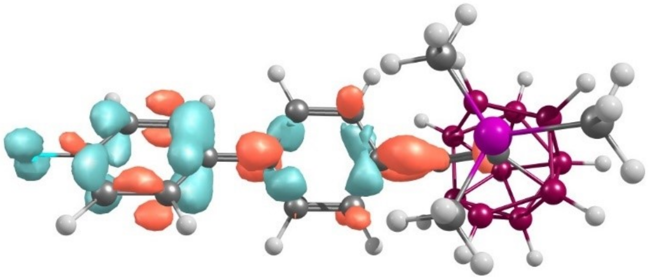 S8	
2M	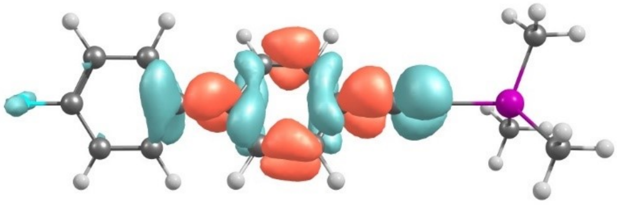 S1	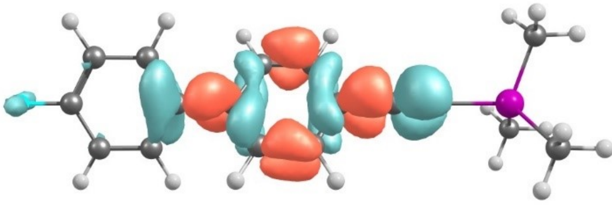 S11
2H	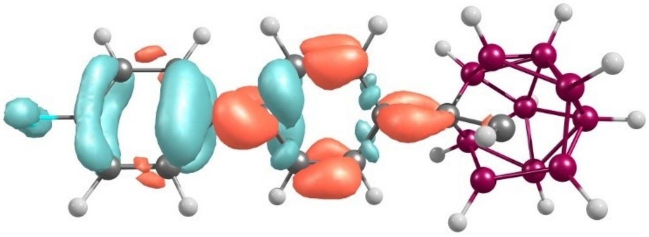 S1	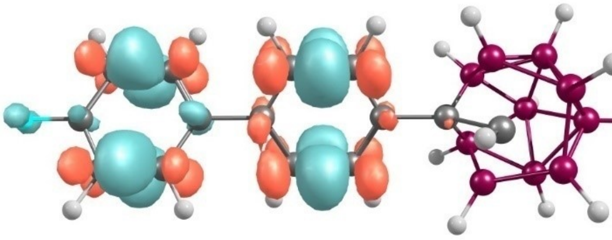 S7
	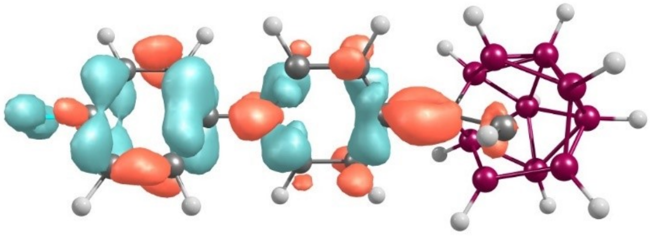 S8	
3H	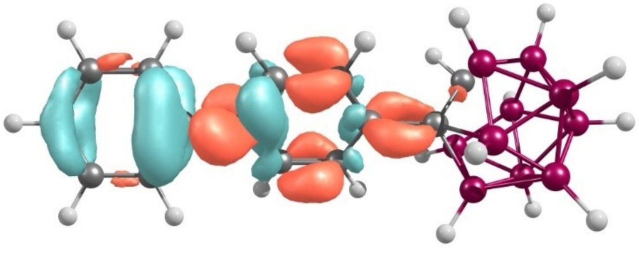 S1	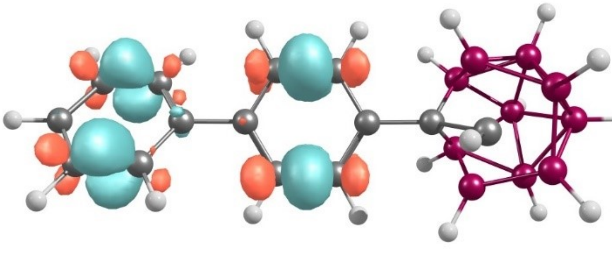 S7
3C	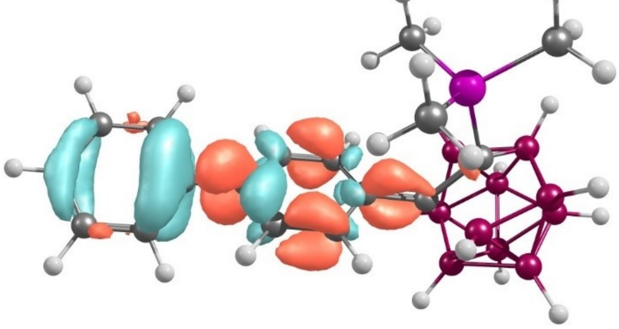	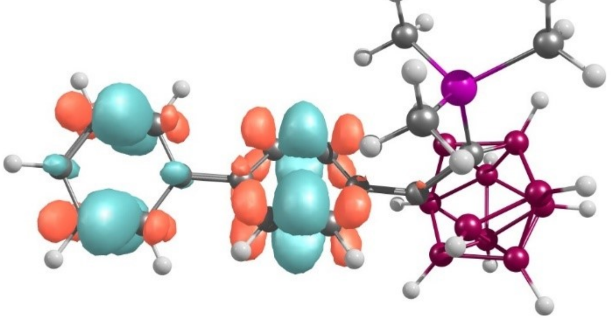

	S1	S7
	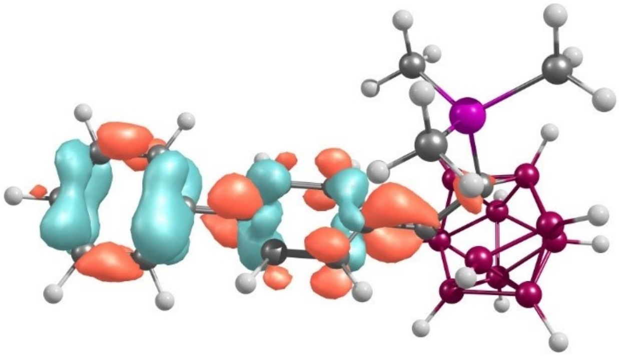 S8	
3M	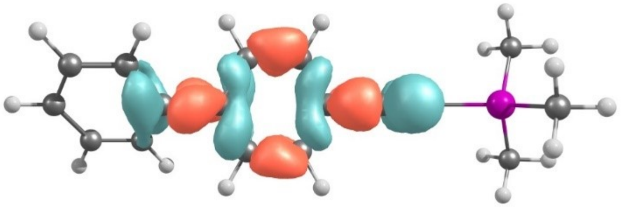 S1	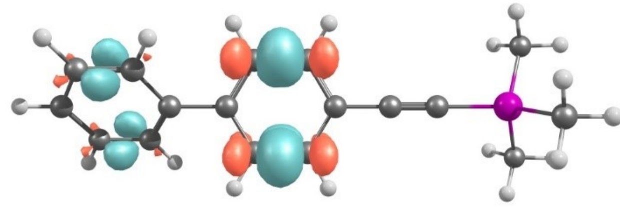 S11
	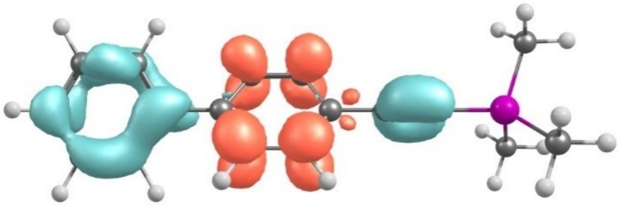 S14	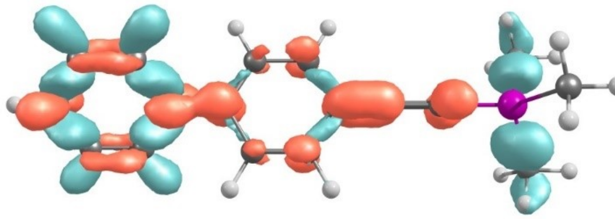 S23
4C	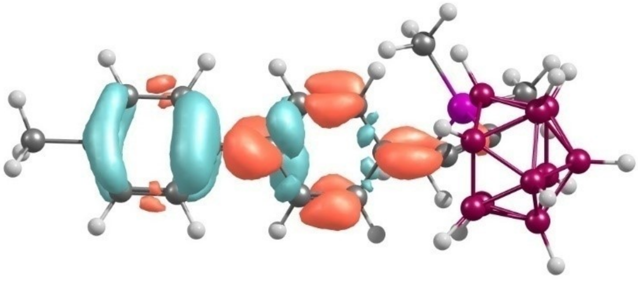 S1	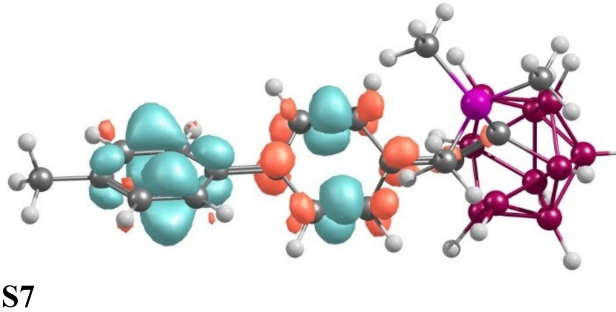 S7
	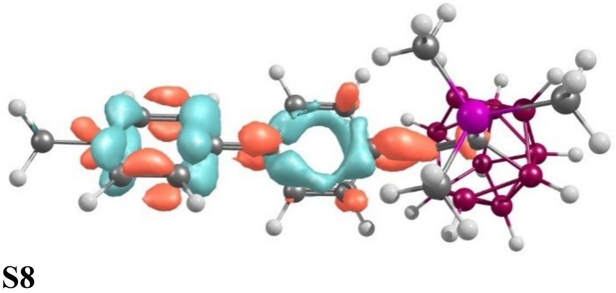 S8	
4M	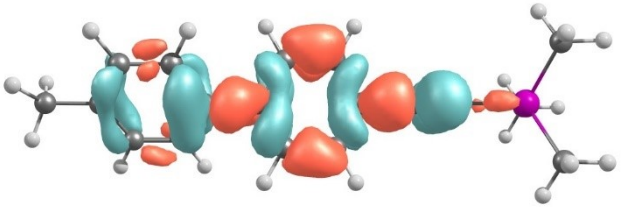 1S	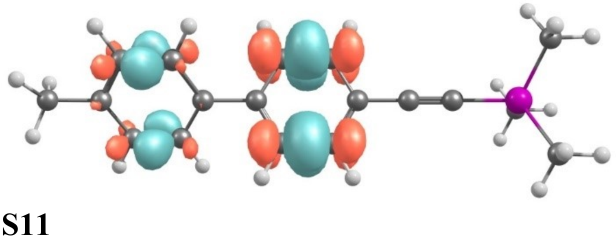 S11

**4MH**	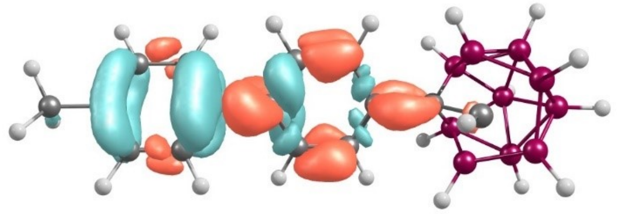 **S1**	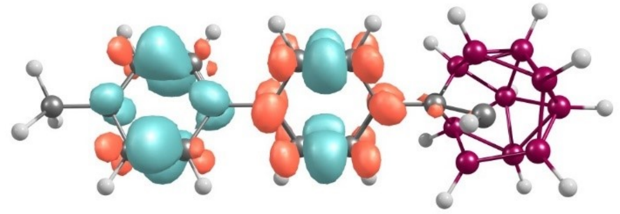 **S7**
	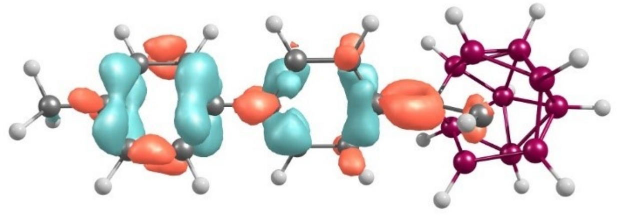 **S8**	
**5C**	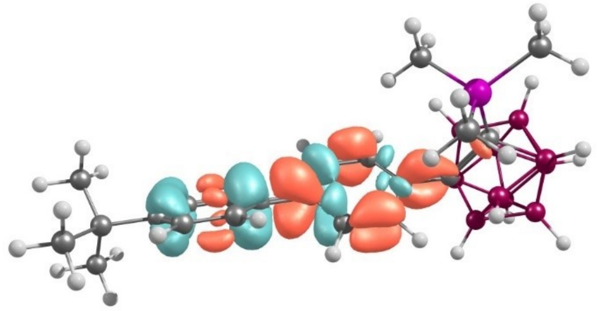 **S1**	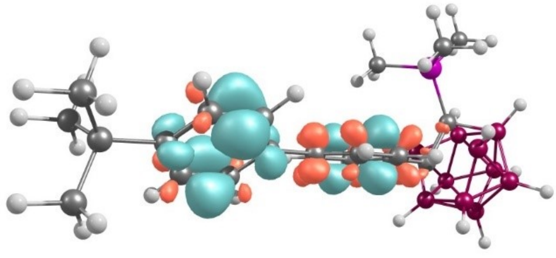 **S7**
**5M**	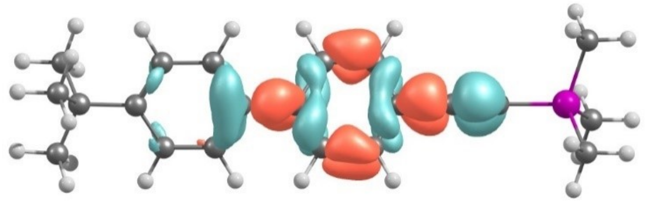 **S1**	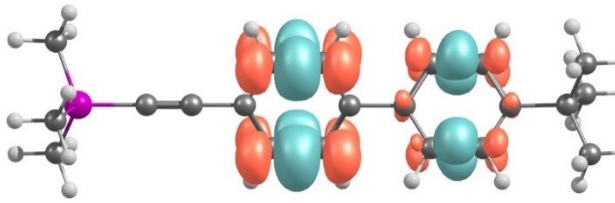 **S11**
	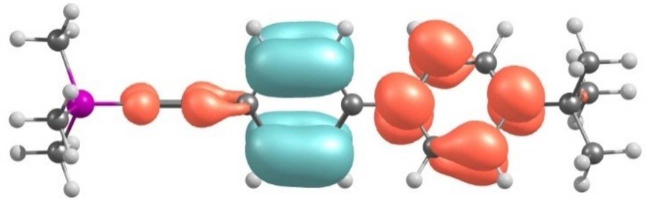 **S24**	
**5H**	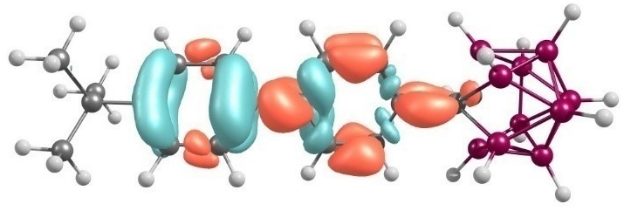 **S1**	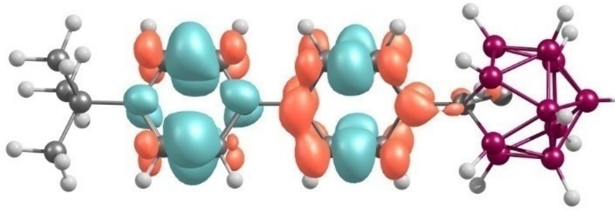 **S7**
	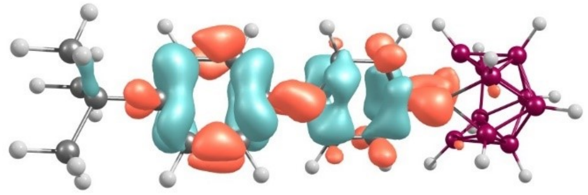	

	**S8**	
**6M**	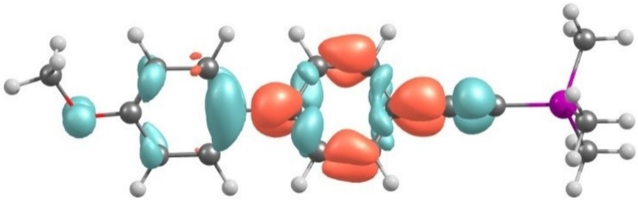 **S1**	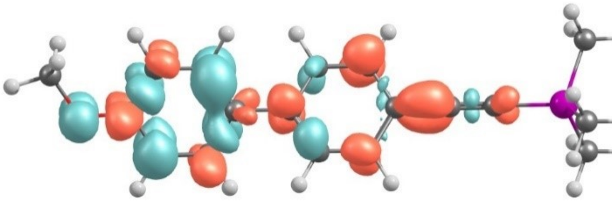 **S9**
	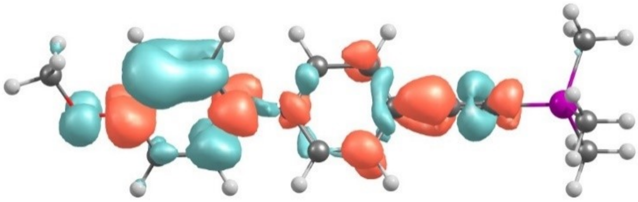 S21	
**6H**	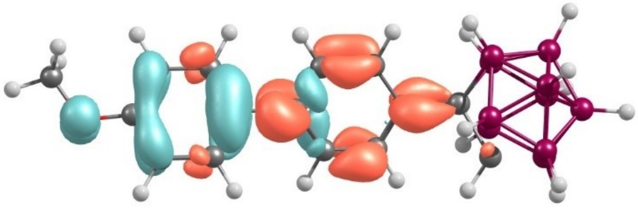 S1	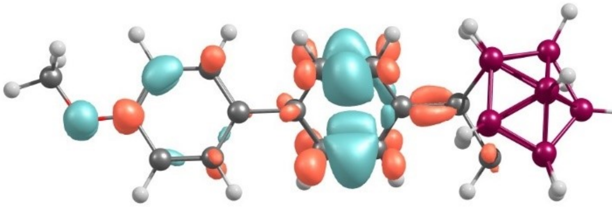 **S8**
**6C**	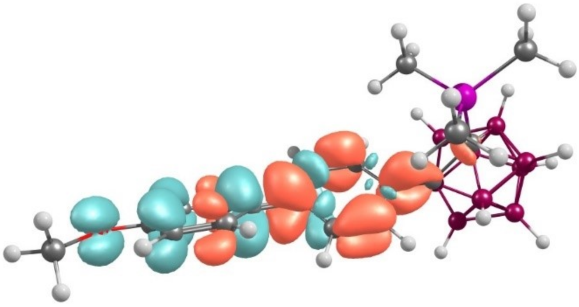 S1	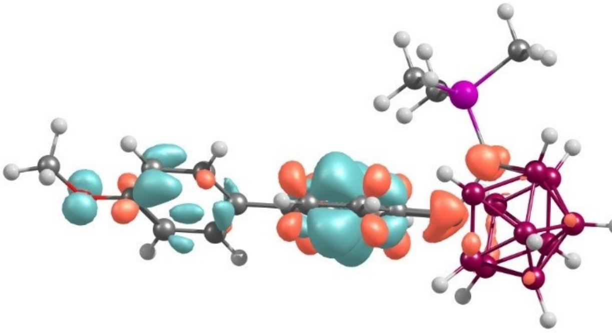 **S9**
**7C**	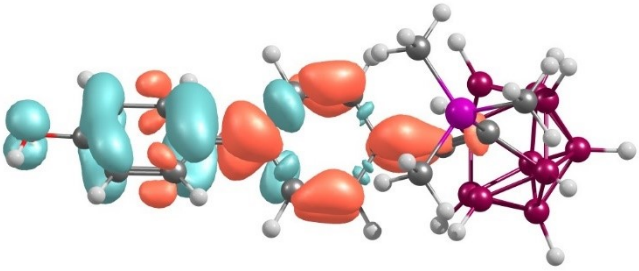 S1	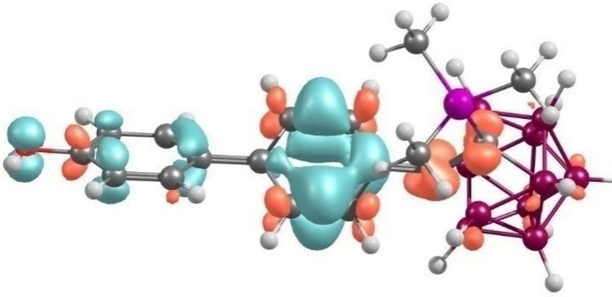 **S9**
**7M**	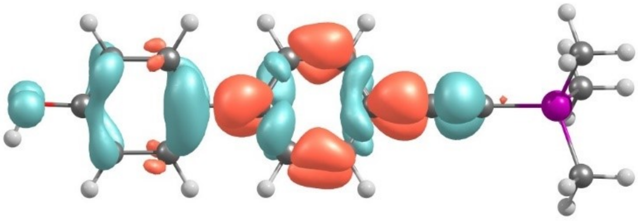 S1	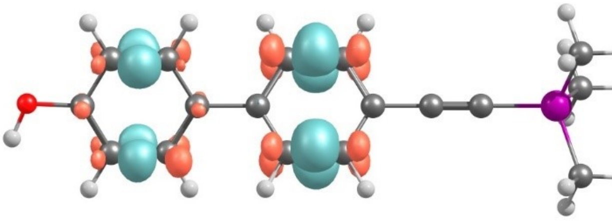 **S11**

	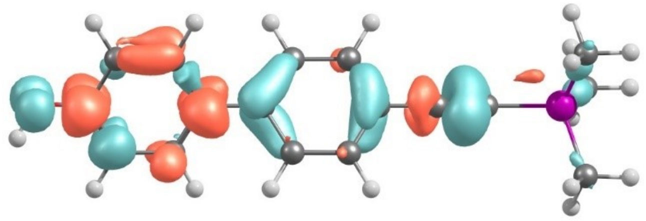 S22	
**7H**	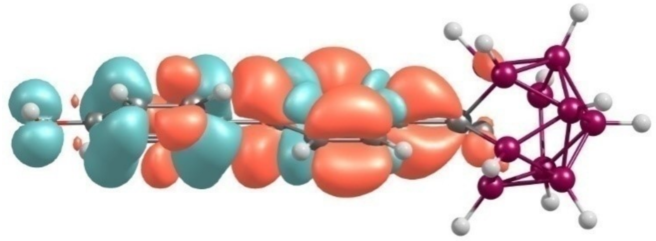 S1	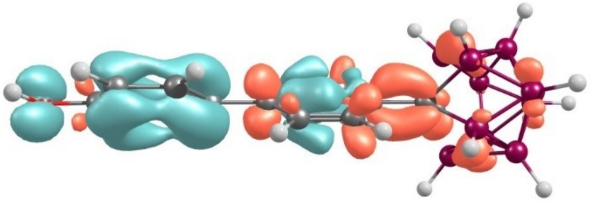 **S7**
	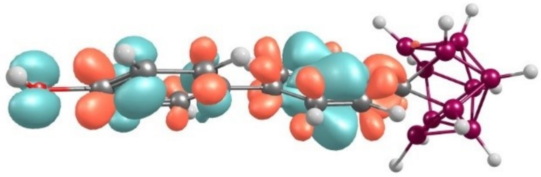 S8	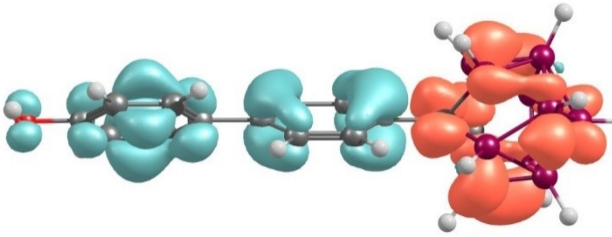 **S9**
**8C**	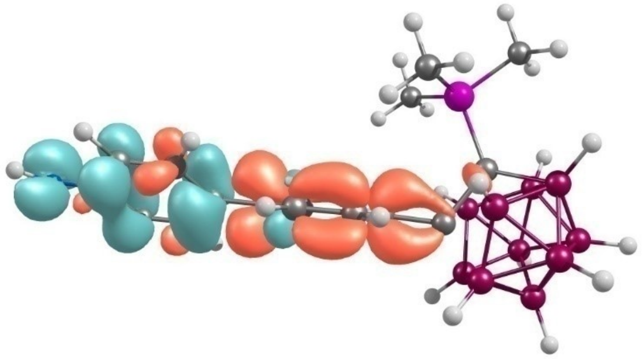 **S1**	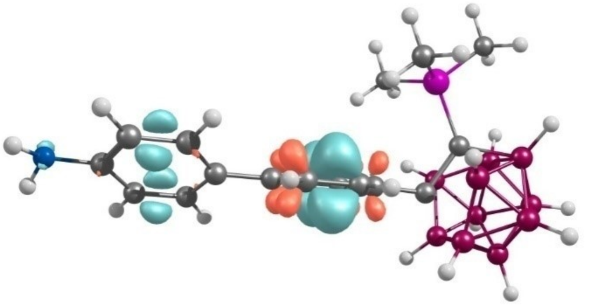 **S9**
**8M**	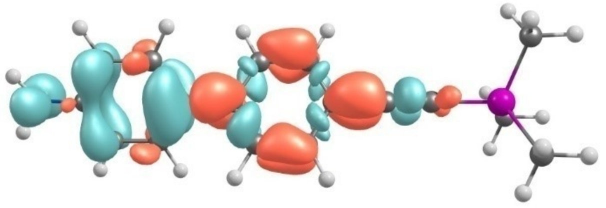 **S1**	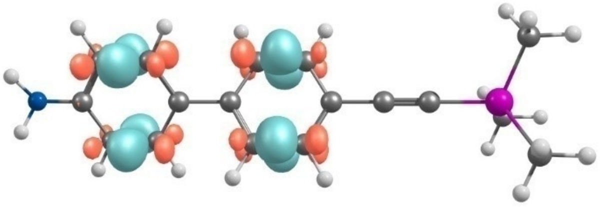 **S12**
	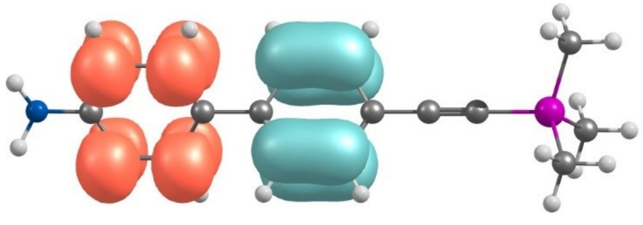 **S23**	

**8H**	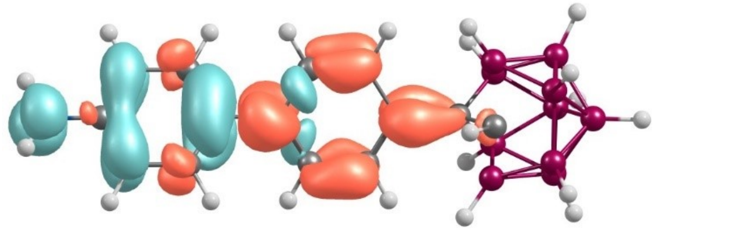 **S1**	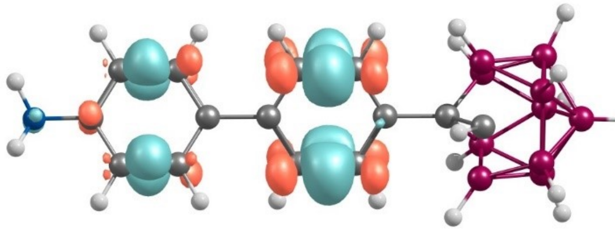 **S9**
**9M**	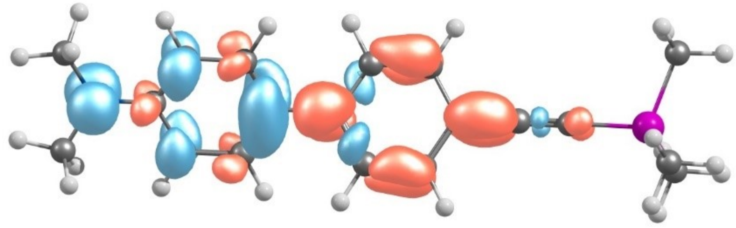 S1	
**9H**	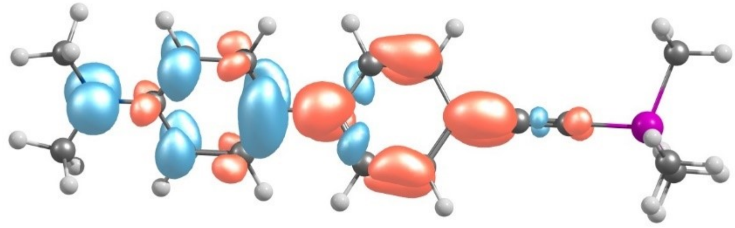 S1	
**9C**	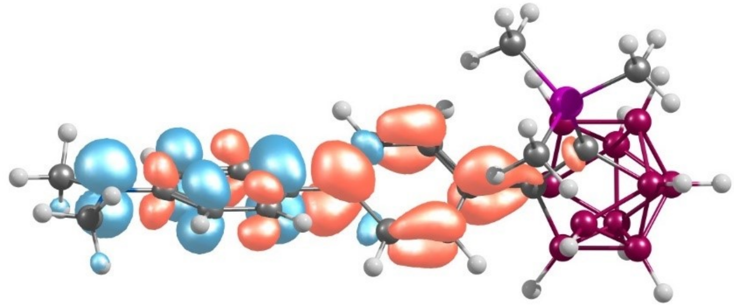 S1	

Figure S2.CDD for different excited states of the title compounds. CDD was calculated as a difference between the corresponding excited state and the ground state of the considered system using the CAM-B3LYP/6-31G(d,p)/IEFPCM level of theory. Blue regions indicate negative electron density, whereas orange regions correspond to positive electron density. The isosurface level is set to be 0.001807.

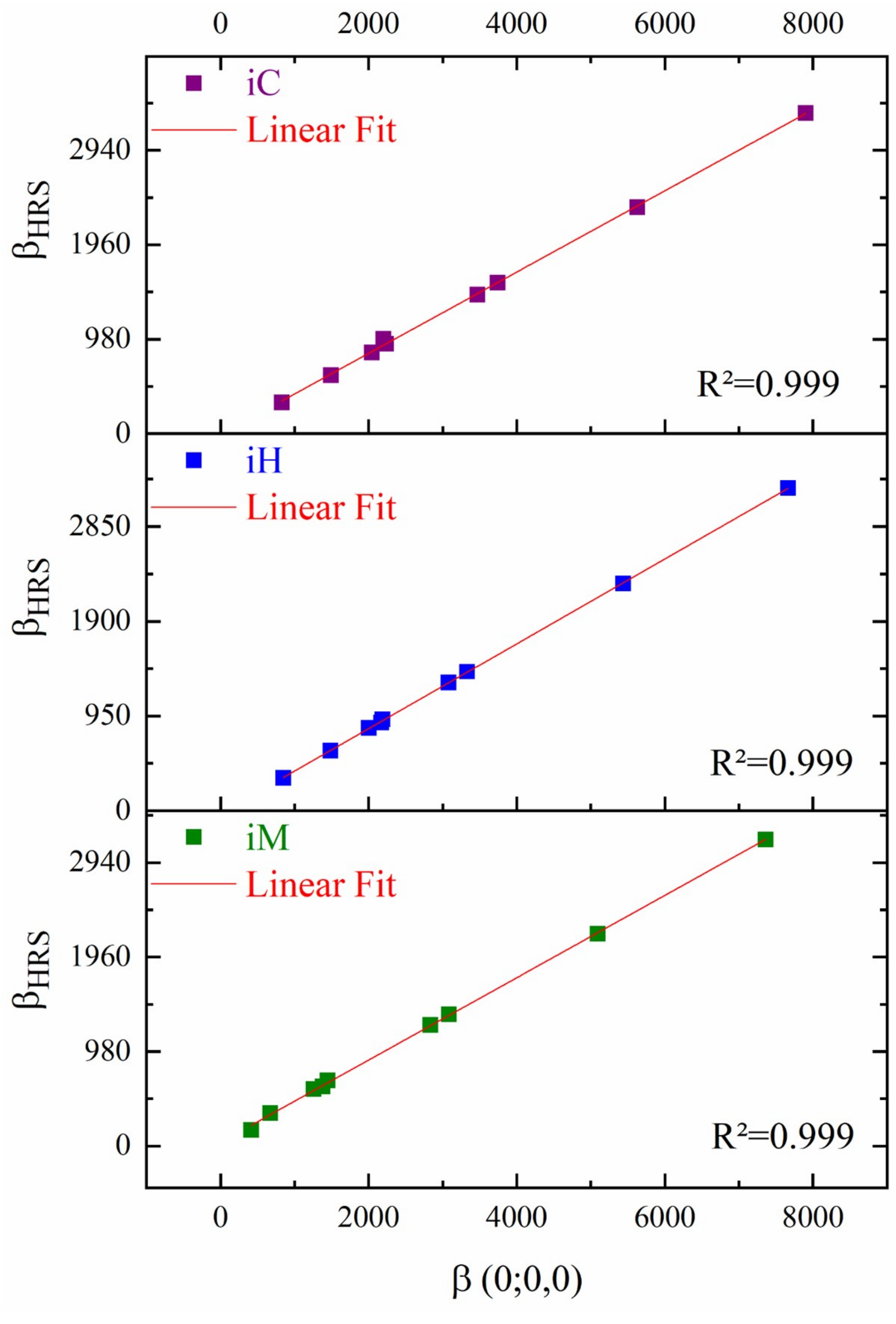

Figure S3. Correlation between static first hyperpolarizability β_{HRS} and β(0;0,0) for iM, iH and iC compounds (i=1 to 9), calculated at the CAM-B3LYP/6-31G(d,p)/IEFPCM level in THF.

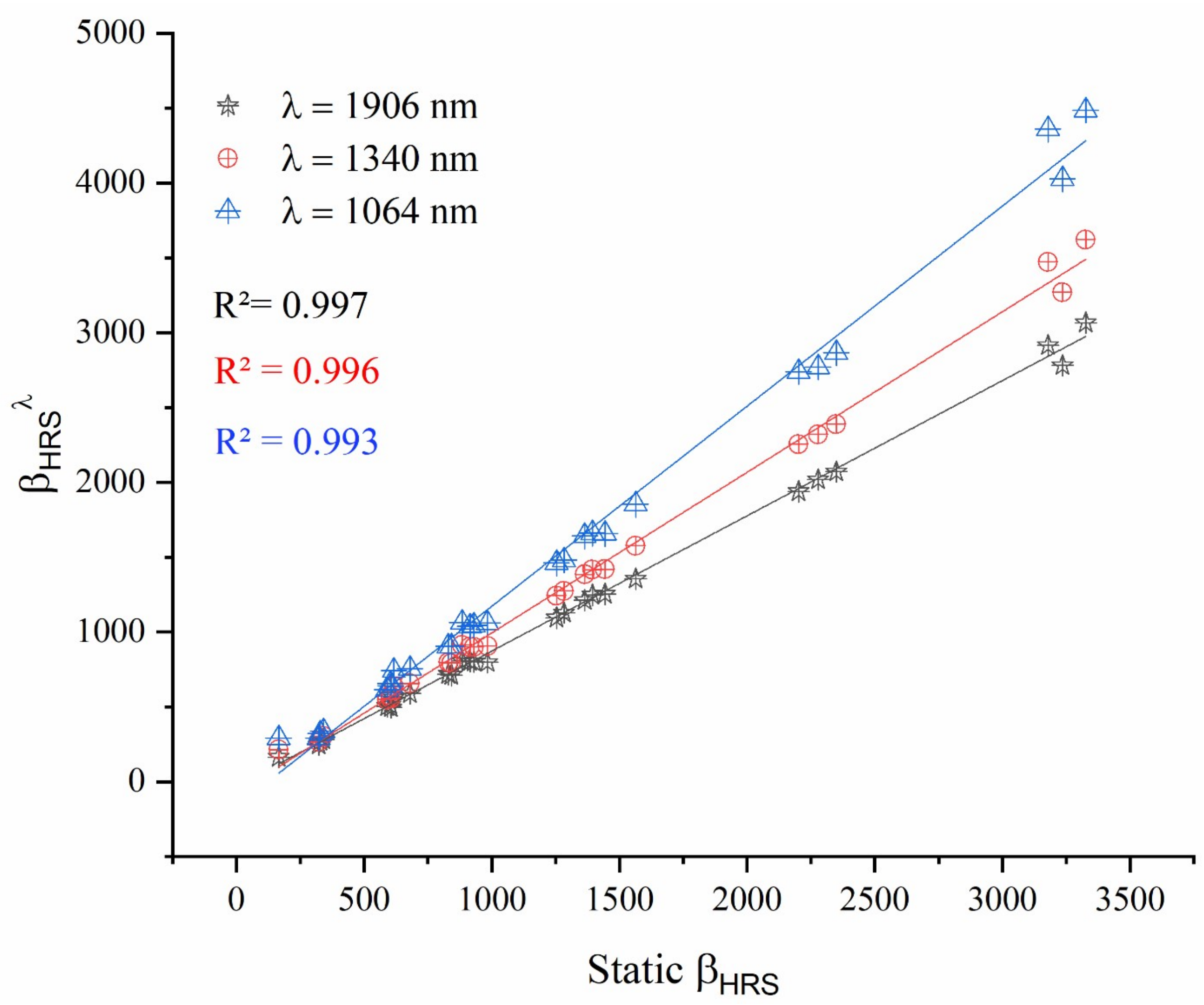

Figure S4. Correlation between dynamic and static First Hyperpolarizability β_{HRS} for iM, iH and iC compounds (i=1 to 9), calculated at the CAM-B3LYP/6-31G(d,p)/IEFPCM level in THF.

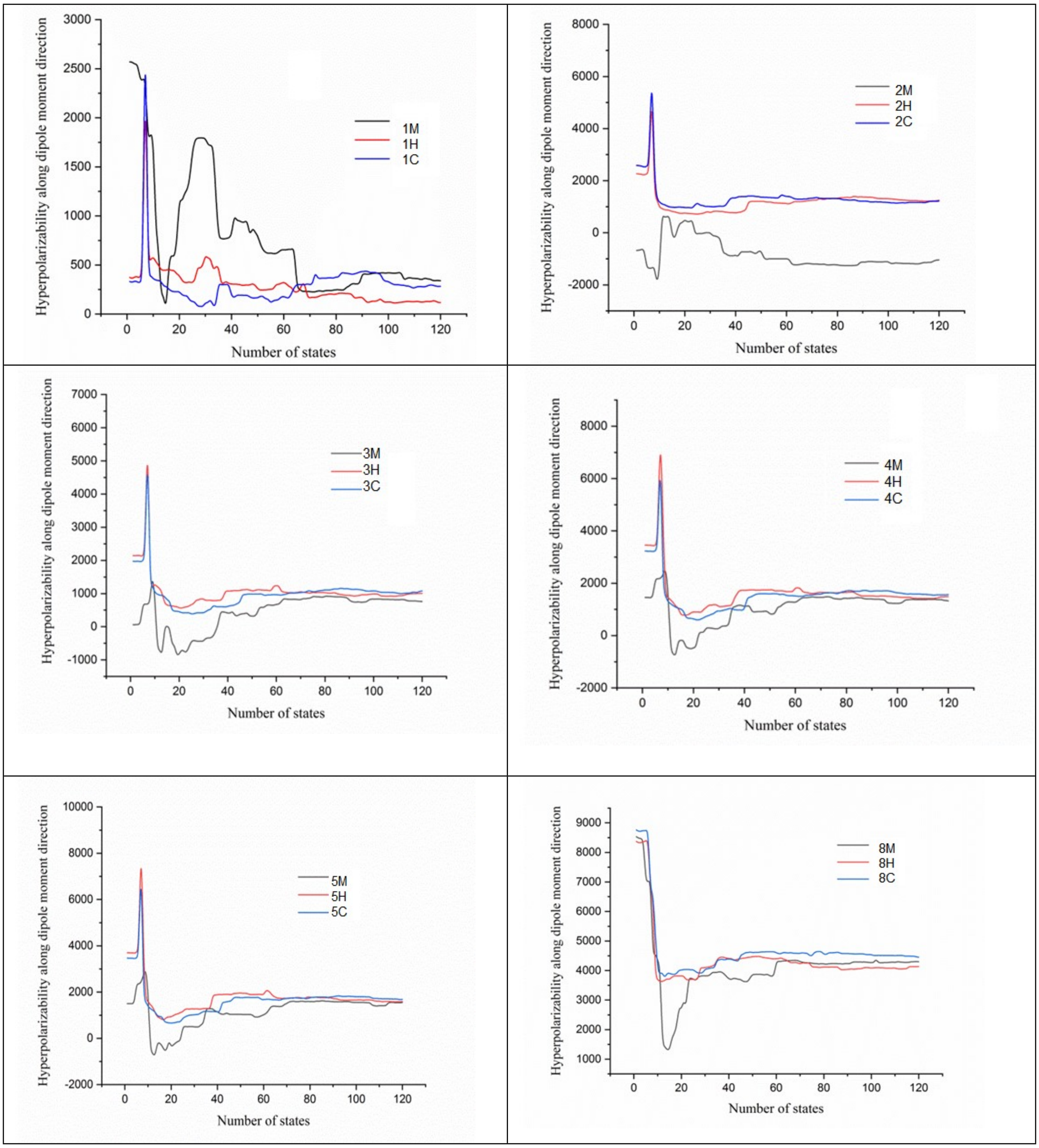

Figure S5. Plots of static first hyperpolarizability values as computed in the SOS formalism as a function of the number of excited states for foriM, iH and iC compounds (i=1-5,8), calculated at the CAM-B3LYP/6-31G(d,p)/IEFPCM level in THF.

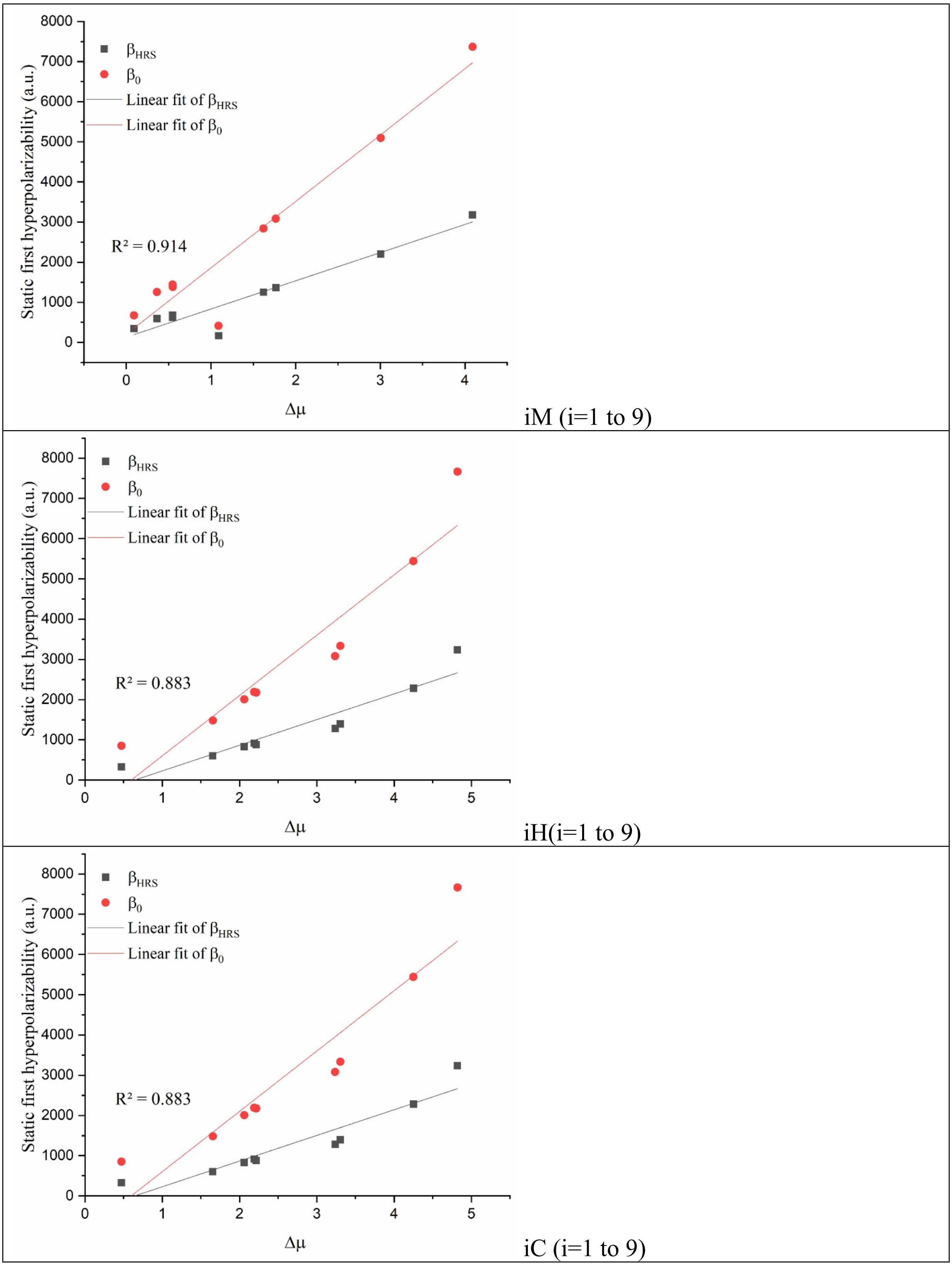

Figure S6. Linear Correlation Between First Hyperpolarizability and Dipole Moment Variation (Δμ) in the First Excited State for iM, iH and iC (i=1 to 9) compounds

Refences

(1) You, D. K.; Kim, M.; Kim, D.; Kim, N.; Lee, K. M. Improvement in Radiative Efficiency Via Intramolecular Charge Transfer in Ortho-Carboranyl Luminophores Modified with Functionalized Biphenyls. *Inorg. Chem.* **2023**, *62*, 10003–10013. https://doi.org/10.1021/acs.inorgchem.3c01242.
